# Pharmacogenetic Variants and Plasma Concentrations of Antiseizure Drugs: A Systematic Review and Meta-Analysis

**DOI:** 10.1001/jamanetworkopen.2024.25593

**Published:** 2024-08-08

**Authors:** Filip Milosavljević, Marina Manojlović, Lena Matković, Espen Molden, Magnus Ingelman-Sundberg, Stefan Leucht, Marin M. Jukić

**Affiliations:** 1 Department of Physiology, Faculty of Pharmacy, University of Belgrade, Belgrade, Serbia; 2 Department of Psychiatry and Psychotherapy, School of Medicine, Technische Universität München, München, Germany; 3 Institute for Mental Health, Belgrade, Serbia; 4 Section for Pharmacology and Pharmaceutical Biosciences, Department of Pharmacy, University of Oslo, Oslo, Norway; 5 Center for Psychopharmacology, Diakonhjemmet Hospital, Oslo, Norway; 6 Pharmacogenetics Section, Department of Physiology and Pharmacology, Karolinska Institutet, Stockholm, Sweden

## Abstract

**Importance:**

Precise estimation of a patient’s drug metabolism capacity is important for antiseizure dose personalization.

**Objective:**

To quantify the differences in plasma concentrations for antiseizure drugs associated with variants of genes encoding drug metabolizing enzymes.

**Data Sources:**

PubMed, Clinicaltrialsregister.eu, ClinicalTrials.gov, International Clinical Trials Registry Platform, and CENTRAL databases were screened for studies from January 1, 1990, to September 30, 2023, without language restrictions.

**Study Selection:**

Two reviewers performed independent study screening and assessed the following inclusion criteria: appropriate genotyping was performed, genotype-based categorization into subgroups was possible, and each subgroup contained at least 3 participants.

**Data Extraction and Synthesis:**

The Meta-analysis of Observational Studies in Epidemiology (MOOSE) guidelines were followed for data extraction and subsequent quality, validity, and risk-of-bias assessments. The results from the included studies were pooled with random-effect meta-analysis.

**Main Outcomes and Measures:**

Plasma concentrations of antiseizure drugs were quantified with the dose-normalized area under the concentration-time curve, the dose-normalized steady state concentration, or the concentrations after a single dose at standardized dose and sampling time. The ratio of the means was calculated by dividing the mean drug plasma concentrations of carriers and noncarriers of the pharmacogenetic variant.

**Results:**

Data from 98 studies involving 12 543 adult participants treated with phenytoin, valproate, lamotrigine, or carbamazepine were analyzed. Studies were mainly conducted within East Asian (69 studies) or White or European (15 studies) cohorts. Significant increases of plasma concentrations compared with the reference subgroup were observed for phenytoin, by 46% (95% CI, 33%-61%) in *CYP2C9* intermediate metabolizers, 20% (95% CI, 17%-30%) in *CYP2C19* intermediate metabolizers, and 39% (95% CI, 24%-56%) in *CYP2C19* poor metabolizers; for valproate, by 12% (95% CI, 4%-20%) in *CYP2C9* intermediate metabolizers, 12% (95% CI, 2%-24%) in *CYP2C19* intermediate metabolizers, and 20% (95% CI, 2%-41%) in *CYP2C19* poor metabolizers; and for carbamazepine, by 12% (95% CI, 3%-22%) in *CYP3A5* poor metabolizers.

**Conclusions and Relevance:**

This systematic review and meta-analysis found that *CYP2C9* and *CYP2C19* genotypes encoding low enzymatic capacity were associated with a clinically relevant increase in phenytoin plasma concentrations, several pharmacogenetic variants were associated with statistically significant but only marginally clinically relevant changes in valproate and carbamazepine plasma concentrations, and numerous pharmacogenetic variants were not associated with statistically significant differences in plasma concentrations of antiseizure drugs.

## Key Points

**Question:**

Are variants of genes encoding drug metabolizing enzymes associated with plasma concentration of antiseizure drugs?

**Findings:**

This systematic review and meta-analysis of 98 studies involving 12 543 participants provided quantification of differences in plasma concentrations of phenytoin, valproate, lamotrigine, and carbamazepine among cohorts defined by pharmacogenetic polymorphisms.

**Meaning:**

These results provide a scientific basis for *CYP2C9* and *CYP2C19* genotype-based dosing recommendations for phenytoin and suggest that numerous pharmacogenetic variants previously associated with the drug metabolism of valproate, carbamazepine, and lamotrigine exhibit only marginal, if any, clinical relevance.

## Teaser

This systematic review and meta-analysis quantifies differences in plasma concentrations for antiseizure drugs associated with variants of genes encoding drug metabolizing enzymes, particularly *CYP2C9* and *CYP2C19*.

## Introduction

The variability of the pharmacokinetics of antiseizure drugs is considerable, leading to significant interindividual variations in plasma concentrations. The metabolism and disposition of many antiseizure drugs is facilitated by polymorphic metabolizing enzymes whose activities are genetically determined.^1^ As a result, considerable research efforts have been made to identify and validate variations in genes encoding these enzymes that can be used to predict plasma concentrations and subsequently individualize the dose of antiseizure drugs. However, the results of these studies have often remained inconclusive, as many of them were not sufficiently powerful to accurately quantify the difference between subgroups determined by genotype and to assess their clinical relevance. Subsequently, several meta-analyses^2,3,4,5,6,7,8,9,10,11,12^ have attempted to address the problem of insufficient power by pooling data from published reports on the most promising associations between pharmacogenetic variants and variations in antiseizure drug concentrations. However, a critical review shows that many of these meta-analyses either used inappropriate methods or included only subsets of all available studies (Table 1).

**Table 1.** Comparison of the Previous and the Current Meta-Analyses

Meta-analysis	Drug-gene interaction	Comment	Trials included in meta-analysis, No.	
			Reference	This study
Kanjanasilp et al,^2^ 2021	Phenytoin-*CYP2C9*	Underpowered; results were highly influenced by 1 study; Michaelis-Menten constant was assessed and not C/D	4	20
	Phenytoin-*CYP2C19*		8	12
Liao et al,^4^ 2018	Phenytoin-CYP2C9	Underpowered; Michaelis-Menten constant was assessed and not C/D	6	20
	Phenytoin-*CYP2C19*		6	12
Fang et al,^3^ 2021	Valproate-*CYP2C9*	Analyzed C/D; several eligible trials were omitted	6	12
Yoon et al,^5^ 2020	Valproate-*CYP2C9*	Analyzed C/D; several eligible trials were omitted even after accounting for inclusion criteria	5	21
Kim et al,^6^ 2019	Valproate-*UGT1A6*	Analyzed C/D; several eligible trials were omitted	6	25
Wang et al,^7^ 2018	Valproate-*UGT2B7*	Analyzed C/D; many eligible trials were published after this manuscript; omitted few eligible trials even after accounting for search date	9	23
Li et al,^8^ 2018	Lamotrigine-*UGT1A4*	Limited scope: focused only on Chinese cohorts	6	10
Kim et al,^9^ 2018	Lamotrigine-*UGT1A4*	Analyzed C/D; several eligible trials were published after this study; omitted few eligible trials even after accounting for search date	5	12
	Lamotrigine-*UGT2B7*		3	7
Hu et al,^10^ 2021	Carbamazepine-*EPHX1*	Included the same set of studies as the current meta-analysis if the search date is taken into account; results did not account for the active metabolite	6	7
Zhang et al,^11^ 2021	Carbamazepine-*EPHX1*	Included the same set of studies as the current meta-analysis if the search date is taken into account; the metabolite and parent drug are analyzed separately	4	5
Zhao et al,^12^ 2021	Carbamazepine-*CYP3A5*	Few eligible trials were omitted even after accounting for strict inclusion criteria; the metabolite and parent drug are analyzed separately	8	13

Abbreviation: C/D, concentration-to-dose ratio.

Precise and accurate quantification of pharmacogenetic associations is critical to determine their relevance to clinical practice and subsequently implement genotype-guided dose recommendations tailored for specific subpopulations. Recently, for example, we and others have demonstrated the clinical utility^13^ and cost-effectiveness^14^ of personalizing the dose of psychiatric drugs using pharmacogenetic testing based on variations in the genes encoding drug-metabolizing enzymes *CYP2C19* (OMIM: 124020) and *CYP2D6* (OMIM: 608902). As using a similar approach could potentially be a way to improve treatment with antiseizure drugs, the aim of this systematic review and meta-analyses of prospective and retrospective cohort studies was to investigate whether variants in genes encoding drug-metabolizing enzymes were associated with significantly altered plasma concentrations of antiseizure drugs and to distinguish between marginal and clinically relevant differences caused by specific pharmacogenetic variants.

## Methods

The protocol for the systematic review and the statistical methods were pre-registered via the PROSPERO platform (identifier: CRD42023387703). The meta-analyses were conducted in accordance with the Meta-analysis of Observational Studies in Epidemiology (MOOSE) reporting guideline.

### Principle Parameters for the Analysis

Initially, all clinically relevant antiseizure drugs were considered for analysis.^1^ Gabapentin, topiramate, pregabalin, levetiracetam, and felbamate were then excluded because they are predominantly excreted unchanged via the kidneys.^15^ Tiagabine and clonazepam were not included because they are not metabolized by enzymes with a high frequency of functional allelic variants.^16^ Although phenobarbital and clobazam are metabolized by the polymorphic *CYP2C19* enzyme, they were not included because phenobarbital is mainly used acutely for alcohol withdrawal or agitation and clobazam is predominantly used as add-on therapy. Next, for practical reasons, the meta-analysis was only conducted if the total number of participants across all included studies for the given drug-gene interaction was greater than 500. As the data for zonisamide and oxcarbazepine did not fulfill this criterion, only carbamazepine, lamotrigine, phenytoin, and valproate were chosen for the meta-analysis. The enzymes involved in metabolism of these drugs are CYP3A4, CYP3A5, EPHX1, UGT2B7, and CYP2B6 for carbamazepine^17^; UGT1A4, UGT2B7, CYP2A6, and CYP2D6 for lamotrigine^18^; CYP2C9 and CYP2C19 for phenytoin^19^; and UGT1A4, UGT1A6, UGT1A8, UGT1A9, UGT1A10, UGT2B7, UGT2B15, CYP2C9, CYP2B6, and CYP2A6 for valproate.^20^ Each pharmacogenetic association was analyzed separately, and participants were divided into subgroups based on genotype according to previously established guidelines^15,21,22,23,24,25^ (Table 2). Finally, the mean plasma concentrations were compared between the genotype-defined control group and the variant subgroups associated with potentially different drug metabolism compared with the control group.^15,21,22,23,24,25^

**Table 2.** Genetic Polymorphism–Based Categorization of Participants Into Control Group and Groups With Potentially Altered Metabolism

Gene	Variant haplotypes	Control group	Group with potentially altered metabolism (variant)
*CYP2C9*	Decreased activity: CYP2C9*2: rs1799853; Abolished activity: CYP2C9*3: rs1057910	*CYP2C9* norm/norm	Intermediate metabolizers: norm/decreased, decreased/decreased, and norm/null; poor metabolizers: decreased/null and null/null
*CYP2C19*	Abolished activity: CYP2C19*2: rs1799853 or CYP2C19*3: rs1057910	*CYP2C19* norm/norm	Intermediate metabolizers: norm/null; poor metabolizers: null/null
*UGT1A6*	UGT1A6*2: rs6759892, rs2070959, or rs1105879	UGT1A6*2 noncarriers	UGT1A6*2 hemizygotes; UGT1A6*2 homozygotes
*UGT2B7*	UGT2B7*2: rs7439366 or; rs7668258	UGT2B7*2 noncarriers	UGT2B7*2 hemizygotes; UGT2B7*2 homozygotes
	UGT2A7*3: rs12233719	UGT2B7*3 noncarriers	UGT2B7*3 hemizygotes; UGT2B7*3 homozygotes
*UGT1A4*	UGT1A4*3: rs2011425	UGT1A4*3 noncarriers	UGT1A4*3 hemizygotes or homozygotes
*CYP3A5*	CYP3A5*3: rs776746	CYP3A5*3 noncarriers and CYP3A5*3 hemizygotes	CYP3A5*3 homozygotes
*EPHX1*	rs1051740	rs1051740 noncarriers	hemizygotes; homozygotes
	rs2234922	rs2234922 noncarriers	hemizygotes; homozygotes

Abbreviations: decreased, allele associated with the activity substantially lower than that seen in carriers of norm alleles; norm, allele associated with usual enzyme activity as seen in the carriers of the wildtype genotype; null, loss-of-function alleles associated with nonexistent or very low activity of the given enzyme.

### Search Strategy, Selection Criteria, and Data Extraction

The search was conducted in the PubMed, ClinicalTrials.gov, Clinicaltrialsregister.eu, International Clinical Trials Registry Platform and CENTRAL databases for reports published between January 1, 1990, and September 30, 2023. A separate literature search was conducted for each drug, and the search terms are listed in eAppendix 1 in Supplement 1. The references of the included trials and prominent reviews were manually searched. Studies lacking plasma concentrations of drugs were excluded at the first screening; only studies that presented the sum of free and protein-bound drug fractions were included, and the remaining studies were considered for inclusion if they met the following criteria: the gene of interest was genotyped for all of its known functional variants with a minor allele frequency greater than 1%, participants were appropriately assigned to metabolizer categories based on genotyping or the authors presented drug plasma concentration data for individual genotypes in a manner that reclassification into categories was possible, the study included at least 3 participants per experimental group, and plasma concentrations of the drug were presented as dose-normalized plasma concentrations or dose-normalized area under the plasma concentration-time curve after single or multiple dosing, provided that the dose and time between drug intake and plasma concentration measurement were standardized.

Screening and selection of studies were performed independently by 2 investigators (M.M. and L.M.). The decision on inclusion in the analysis was made by consensus with a third investigator (F.M.), with final review by consensus between 2 investigators (F.M. and M.M.J.). Risk of bias (ROB) was assessed in 6 domains using the standardized Risk Of Bias In Non-Randomised Studies of Interventions tool for nonrandomized studies,^26^ and studies with critical ROB grade were excluded. There were no restrictions on study design, participant characteristics (eg, race and ethnicity, sex, age, patients in treatment vs healthy volunteers, smoking status, treatment duration, drug interactions), published vs unpublished studies, or language. Studies written in languages other than English were translated by unbiased researchers who were native speakers of respective languages. For carbamazepine, plasma concentration was presented as active moiety, ie, the sum of plasma concentrations of carbamazepine and its active metabolite carbamazepine-10,11-epoxide. Where available, the means and SDs for the available parameter for the plasma concentration of the drug and the number of patients per genotype-defined metabolizer subgroup were taken directly from the report. Otherwise, established procedures for data transformation or graph extraction were performed.^27^ If this was not possible, the authors were contacted to provide the required data, as described in eTable 1 in Supplement 1.

### Statistical Data Analyses

The effect size was quantified as the ratio of means (ROM), ie, the mean drug plasma concentrations of the variant group divided by the mean drug plasma concentrations of the control group.^28^ The standard mean differences (Hedges *g*) were also calculated. Between-study heterogeneity was assessed using the Cochran Q test (threshold *P* < .10), while the percentage of total variability attributable to heterogeneity was quantified by the *I*^2^ value. Due to the expected heterogeneity between studies, the weighted ROM between groups was used to calculate the pooling effect between studies using a random-effects meta-analysis model.

Small-study effects and potential publication bias were assessed using the Egger test^29^ and contour-enhanced funnel plot asymmetry.^30^ *P* < .10 was considered significant, and the funnel plots are presented in eFigures 27 through 30 in Supplement 1. Statistical analyses were performed using RevMan software, version 5.4 (Cochrane). ROMs for each study were calculated using Excel 2016 (Microsoft) according to the previously published formula^13,28^ and then entered into the RevMan software using the generic inverse variance option. Two-sided α < .05 was interpreted as a statistically significant difference. The effects of race and ethnicity, age, study design, and degree of ROB on the results of the meta-analysis and the overall robustness of the results are investigated in detail in sensitivity analyses that were performed by comparing original analysis and the alternative analysis or by comparing 2 alternative analyses where appropriate, with the test of subgroup differences function in RevMan 5.4. The sensitivity analyses of populations of different racial ethnic backgrounds and the sensitivity analysis of studies with different risk-of-bias grades were prespecified, while other sensitivity analyses were performed post-hoc. Race and ethnicity were presented as reported in the original studies. For the purpose of sensitivity analysis, we used 3 categories: White (if a study reported the cohort as being predominantly Caucasian, European, or White); East Asian (if a study reported the cohort as being predominantly Chinese, Japanese, Korean, or Taiwanese); South Asian (if a study reported the cohort as being predominantly Bangladeshi, Indian, or Sri Lankan). Due to scarcity of studies, all other races and ethnicities were presented as a separate category in the sensitivity analysis.

### Interpretation of Clinical Relevance of Pharmacogenetic Associations

The quantitative cutoff for clinical relevance was based on the US Food and Drug Administration bioequivalence cutoffs (ROM: 0.80-1.25),^31^ ie, if the entire 95% CI for the difference in drug plasma concentration between variant and control group was more than 1.25-fold or less than 0.8-fold, such an effect was considered clinically relevant. Statistically significant results not fulfilling this criterion or showing poor robustness in the sensitivity test were considered ambiguous regarding their clinical relevance. Statistically significant results with their 95% CIs completely within the 0.8 to 1.25 ROM range were considered to be of minor clinical relevance.

## Results

**Figure 1.**
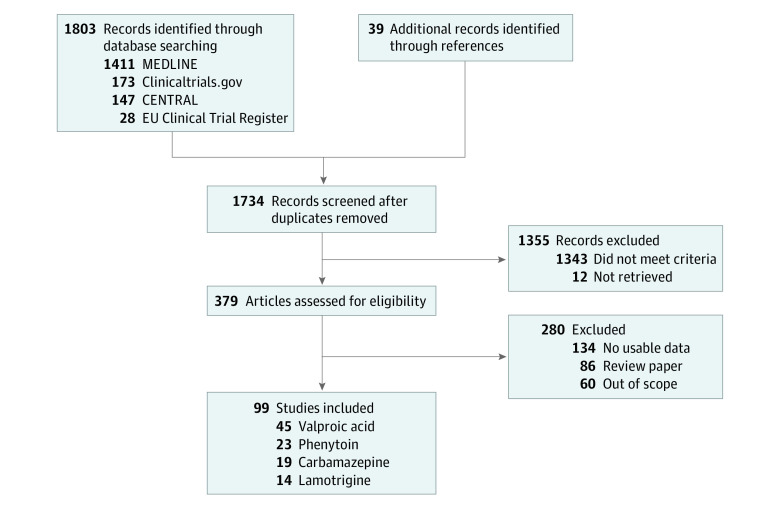
Flowchart of Systematic Review

Of the 1736 references initially reviewed, 98 unique studies^22,32,33,34,35,36,37,38,39,40,41,42,43,44,45,46,47,48,49,50,51,52,53,54,55,56,57,58,59,60,61,62,63,64,65,66,67,68,69,70,71,72,73,74,75,76,77,78,79,80,81,82,83,84,85,86,87,88,89,90,91,92,93,94,95,96,97,98,99,100,101,102,103,104,105,106,107,108,109,110,111,112,113,114,115,116,117,118,119,120,121,122,123,124,125,126,127,128^ with 12 543 unique participants met the inclusion criteria. A summary of the screening results and the reasons for exclusion are shown in Figure 1, while the flow diagrams for the individual drugs and the detailed lists of included studies can be found in eFigures 1 to 4 in Supplement 1. Most of the included studies were prospectively conducted in neurological patients who had taken multiple doses of medication and reached steady state. Of 98 included studies, 12 studies^34,38,48,57,63,64,77,80,103,104,120,121^ had a retrospective design, and 6 studies^32,33,45,50,52,90^ included healthy volunteers who had taken a single dose of medication under standardized conditions. The included studies were mainly conducted with East Asian (69 studies) and White or European (15 studies) cohorts, while the age of the included participants varied considerably in the available studies; the demographic cohort characteristics and study design of the included studies are detailed in eTables 2 through 9 in Supplement 1. ROB analysis revealed that 45 studies had moderate ROB and 45 studies[ref numbers] had serious ROB, while 9 studies had insufficient data to assess ROB. No study received a low ROB rating; even the studies with a very robust design received a moderate ROB rating because all included studies were naturalistic and therefore it was not possible to completely eliminate the risk of confounding. Besides confounding, the most common issues that led to a disadvantageous ROB rating were inconsistent drug concentration measuring times, and reporting data for only a subset of the tested cohort.

**Figure 2.**
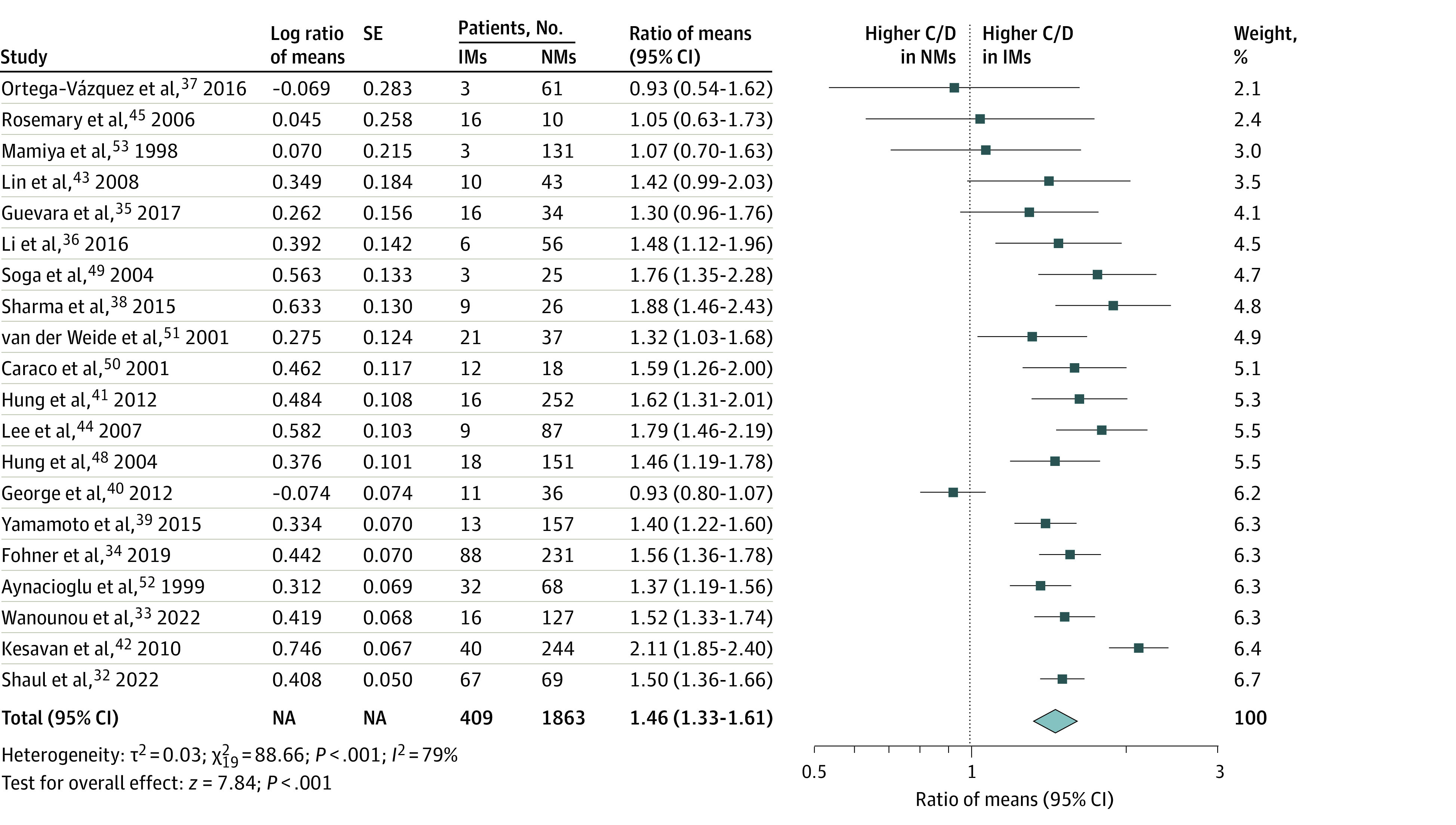
Clinically Relevant Association Between *CYP2C9* Genotype and Phenytoin Plasma Concentration C/D indicates concentration-to-dose ratio; IM, CYP2C9 intermediate metabolizer (carrier of CYP2C9*1/*2, CYP2C9*1/*3, and CYP2C9*2/*2 diplotypes); NA, not applicable; NM, CYP2C9 normal metabolizer (carrier of CYP2C9*1/*1 diplotype).

Sufficient data were available to meaningfully quantify the difference in phenytoin plasma concentrations between the different CYP2C9 and CYP2C19 metabolizer phenotypes. The CYP2C9 intermediate metabolizers had 46% (95% CI, 33%-61%) higher phenytoin plasma concentrations compared with the CYP2C9 normal metabolizers (Figure 2 and Table 3). Insufficient data were available for a meaningful analysis of the association between the very rare CYP2C9 poor metabolizers phenotype and differences in phenytoin plasma concentrations. However, the only study suitable for inclusion, which included 5 CYP2C9 poor metabolizers and 41 CYP2C9 normal metabolizers, showed a very profound increase in phenytoin plasma concentration of 134% in poor metabolizers compared with normal metabolizers.^129^ We observed 23% (95% CI, 17%-30%) higher phenytoin plasma concentration in CYP2C19 intermediate metabolizers and 39% (95% CI, 24%-56%) higher phenytoin plasma concentration in CYP2C19 poor (Table 3). Funnel plots and sensitivity analyses considering only large studies, only studies with adults, studies with different ROB grades, studies with different designs and other variables show a high robustness of the observed differences in phenytoin plasma concentrations (eFigure 27 and eTables 14-16 in Supplement 1). A significant asymmetry was only observed in the funnel plot with respect to the comparison of CYP2C9 intermediate metabolizers and normal metabolizers, suggesting that the results may even be slightly underestimated. In summary, genotypic variants encoding slow CYP2C9 and CYP2C19 metabolism were associated with statistically significant and clinically relevant increases in phenytoin plasma concentrations.

**Table 3.** Quantification of Associations of Genetic Polymorphism in Genes Encoding Drug Metabolizing Enzymes With Antiseizure Drug Plasma Concentration

Meta-analysis	Trials, No.	Participants, No.		ROM (95% CI)	*P* value	*I*^2^, %
		Control	Variant			
Phenytoin						
CYP2C9 IMs vs control	20	1863	409	1.46 (1.33-1.61)	<.001	79
CYP2C19 IMs vs control	12	508	607	1.23 (1.17-1.30)	<.001	0
CYP2C19 PMs vs control	8	359	162	1.39 (1.24-1.56)	<.001	48
Valproic acid						
CYP2C9 IMs vs control	15	1960	327	1.12 (1.04-1.20)	.003	59
CYP2C19 IMs vs control	12	768	826	1.12 (1.02-1.24)	.02	83
CYP2C19 PMs vs control	12	768	236	1.20 (1.02-1.41)	.03	89
UGT1A6*2 He vs noncarriers	25	1639	1200	0.91 (0.85-0.97)	.004	84
UGT1A6*2 Ho vs noncarriers	24	1570	220	0.90 (0.80-1.02)	.11	71
UGT2B7*2 He vs noncarriers	23	1216	1291	0.99 (0.91-1.06)	.72	75
UGT2B7*2 Ho vs noncarriers	22	1142	365	1.01 (0.92-1.11)	.84	66
UGT2B7*3 He vs noncarriers	13	1360	506	0.97 (0.93-1.01)	.19	0
UGT2B7*3 Ho vs noncarriers	7	937	47	0.81 (0.62-1.08)	.15	70
Lamotrigine						
UGT1A4*3 He or Ho vs noncarriers	12	1654	499	0.99 (0.82-1.20)	.95	92
UGT2B7*2 He vs noncarriers	7	390	569	1.03 (0.96-1.11)	.36	10
UGT2B7*2 Ho vs noncarriers	5	311	199	1.09 (0.90-1.32)	.36	64
Carbamazepine						
CYP3A5 PMs vs non-PMs	13	572	580	1.12 (1.03-1.22)	.007	66
*EPHX1* 337C He vs noncarriers	7	372	497	0.91 (0.78-1.06)	.23	83
*EPHX1* 337C Ho vs noncarriers	7	372	202	0.93 (0.67-1.29)	.66	95
*EPHX1* 416G He vs noncarriers	5	590	176	1.03 (0.92-1.15)	.65	54
UGT2B7*2 He vs noncarriers	5	318	272	0.95 (0.86-1.05)	.34	46

Abbreviations: He, hemizygous carrier; Ho, homozygous carrier; IM, intermediate metabolizer; PM, poor metabolizer; ROM, ratio of means.

Sufficient data were available to quantify the difference in valproate plasma concentrations between CYP2C9 and CYP2C19 metabolizer phenotypes and between *UGT1A6* (OMIM: 606431) and *UGT2B7* (OMIM: 600068) genotype-defined subgroups, while insufficient data were available for meaningful analyses of valproate plasma concentrations in relation to *UGT1A4* (OMIM: 606429)*, UGT1A8* (OMIM: 606433)*, UGT1A9* (OMIM: 606434)*, UGT1A10* (OMIM: 606435)*, UGT2B15* (OMIM: 600069)*, CYP2B6* (OMIM: 123930), and *CYP2A6* (OMIM: 122720) genotypes. Compared with the respective normal metabolizers, we observed increased valproate plasma concentrations in CYP2C9 intermediate metabolizers (12% [95% CI, 4%-20%]), CYP2C19 intermediate metabolizers (12% [95% CI, 2%-24%]) and CYP2C19 poor metabolizers (20% [95% CI, 2%-41%]) (Table 3). Compared with homozygous carriers of the major *UGT1A6* allele, heterozygous carriers of the UGT1A6*2 allele exhibited a 9% (95% CI, 3%-15%) reduction in valproate plasma concentrations, while the reduction in homozygous UGT1A6*2 carriers did not reach statistical significance (Table 3). Compared with the homozygous carriers of *UGT2B7* wild-type haplotype, valproate plasma concentrations did not differ significantly in heterozygous or homozygous carriers of UGT2B7*2 haplotype or in heterozygous or homozygous carriers of UGT2B7*3 haplotype (Table 3). Funnel plots suggested no publication bias related to the observed statistically significant differences; however, sensitivity analyses suggest questionable robustness of the associations. Altogether, the associations of valproate plasma concentrations with *CYP2C19*, *CYP2C9*, *UGT1A6*, and *UGT2B7* genotypic variants were either absent or minor.

Sufficient data were available to quantify the difference in lamotrigine plasma concentrations between *UGT1A4* and *UGT2B7* genotype-defined subgroups, while insufficient data were available for a meaningful analyses of lamotrigine plasma concentrations in relation to *CYP2A6* and *CYP2D6* genotypes. Lamotrigine plasma concentrations were not significantly different heterozygous or homozygous carriers of UGT2B7*2 haplotypes or in heterozygous carriers of UGT1A4*3 compared with noncarriers of the respective alleles (Table 3). Altogether, the *UGT1A4* and *UGT2B7* genotypes are not associated with significant differences in lamotrigine plasma concentrations.

Regarding carbamazepine, sufficient data were available to quantify the difference in plasma concentrations between the CYP3A5 metabolizer phenotypes and between the phenotypes defined by the *EPHX1* (OMIM: 132810) and *UGT2B7* genotypes, while there were not enough data regarding *CYP3A4* or *CYP2B6* genotypes. CYP3A5 poor metabolizers exhibited a 12% (95% CI, 3%-22%) plasma concentration increase compared with carriers of functional *CYP3A5* haplotypes (Table 3). Compared with respective control groups, carbamazepine plasma concentrations were not significantly different in heterozygous UGT2B7*2 carriers, heterozygous *EPHX1* rs2234922 carriers, heterozygous *EPHX1* rs1051740 carriers, or homozygous *EPHX1* rs1051740 carriers (Table 3). In summary, carbamazepine plasma concentration was subtly increased among CYP3A5 poor metabolizers and there were no associations with *EPHX1* and *UGT2B7* genotypes. Standard mean differences for all results are presnted in eFigures 31 through 52 in Supplement 1

## Discussion

This systematic review and meta-analysis comprehensively quantified the magnitudes of pharmacokinetic drug-gene interactions related to phenytoin, lamotrigine, valproic acid, and carbamazepine. The interindividual variability of plasma concentration of antiseizure drugs poses a challenge for dose personalization. Therapeutic drug monitoring (TDM) is commonly used for dose titration, which is of particular importance when the therapeutic window of plasma concentration is narrow. While TDM directly measures the plasma concentration of the drug and incorporates all sources of variability in drug exposure, TDM testing only becomes applicable when the drug level reaches a steady state.^130^ Therefore, preemptive genotyping has the potential to assist clinicians to choose the initial dose with the best likelihood of achieving therapeutic blood concentration before TDM data are available. This could provide immense clinical benefits, as the rapid control of symptoms and the avoidance of unnecessary adverse drug reactions facilitates patient belief in and adherence to treatment.

Phenytoin has a narrow therapeutic concentration window and is still widely used worldwide for the treatment of epilepsy, with a market share of 9% in the US^131^ and 5% in Japan.^132^ Genetically determined CYP2C9 poor and intermediate metabolizer phenotypes are listed by the US Food and Drug Administration (FDA) as clinically relevant polymorphisms for treatment with phenytoin,^133^ while the FDA drug label advises caution for CYP2C19 and CYP2C9 poor and intermediate metabolizers.^134^ However, there is limited information on the magnitude of plasma concentration increases in the different CYP phenotypes, on guidelines for the dose optimization of CYP2C9 and CYP2C19 intermediate and poor metabolizers, and on the utility of preemptive *CYP2C9* and *CYP2C19* genotyping. Our results suggest that the increase in phenytoin plasma levels in patients carrying multiple *CYP2C19* and *CYP2C9* deleterious alleles may be up to 2-fold compared with noncarriers of these alleles. Preventive *CYP2C9* and *CYP2C19* genotyping may therefore hold the potential to improve the safety of phenytoin treatment, as incoordination, confusion, and motor dysfunction are highly dependent on phenytoin plasma concentrations.^135^ Moreover, even idiosyncratic adverse effects, such as Stevens-Johnson syndrome, appear to be related to *CYP2C9* genotype, phenytoin dose, and plasma concentration.^136,137^ Feasibility and cost-effectiveness analyses of preemptive genotyping in phenytoin pharmacotherapy are needed to appropriately evaluate the clinical utility of such an intervention.

Statistically significant associations were also observed for valproate plasma concentration and *CYP2C9*, *CYP2C19*, and *UGT1A6* genotypes and for carbamazepine plasma concentration and *CYP3A5* genotype. However, these drug-gene interactions were marginal and not sufficient to justify their inclusion in official recommendations or drug labeling. In addition, numerous other polymorphisms annotated in the literature^18,63^ and in FDA drug labels for valproate^138^, lamotrigine^139^ and carbamazepine^140^ as potentially relevant to drug metabolism did not show statistically significant associations with changes in plasma concentrations of the respective drugs. Given the extensive number of studies and participants included in our meta-analysis, it can be assumed that additional studies specifically targeting these associations are not necessary.

### Limitations

This study has some limitations. The main limitation is the possible presence of confounding factors arising from the nature of the studies included in the meta-analysis, which were mainly nonrandomized, open-label, observational studies conducted in a naturalistic setting. Therefore, factors known to influence drug metabolism, such as anthropometric parameters, liver function, kidney function, drug-drug interactions, and metabolism autoinduction or inhibition, could not be fully controlled. Consequently, high *I^2^* values indicated that the heterogeneity between individual study results was substantial, the ROB was substantial in more than half of the included studies, and asymmetry of the funnel plot was sometimes observed, suggesting that the small studies may be biased. However, given the sample size, it is unlikely that any of these circumstances would lead to substantial changes in the effect size of the meta-analyses and subsequent systematic misinterpretation of the results. Next, this analysis included only the total plasma level concentration, a parameter that can be affected by conditions that influence protein binding of the drug, such as hypoalbuminemia and uremia, which is important for treatment with phenytoin and valproate.^141^

Importantly, poor CYP2C9 metabolizer status likely has a very profound effect on plasma concentrations of phenytoin and valproate,^129,142,143^ but due to the low frequency of this phenotype,^21^ the available data were insufficient for meaningful analysis. Furthermore, since most of the studies, especially for valproic acid, are from East Asian cohorts, the generalizability of the obtained results to patients in other areas may be questionable.

## Conclusions

This systematic review and meta-analyses quantifies the associations of *CYP2C9* and *CYP2C19* genotypes and the elevation of phenytoin plasma concentrations, which may serve as a scientific basis for establishing genotype-guided dosing recommendations and indicate the potential need for preemptive *CYP2C9* and *CYP2C19* genotyping in phenytoin treatment. On the contrary, although certain pharmacogenetic polymorphisms previously associated with the metabolism of lamotrigine, valproate, and carbamazepine may retain academic relevance as, for example, components for advanced dosing algorithms, their stand-alone clinical relevance is likely marginal.

## Notes

Supplement 1. **eAppendix.** Systematic Literature Search Strategy **eFigure 1.** Flowchart of Systematic Literature Search for Phenytoin **eFigure 2.** Flowchart of Systematic Literature Search for Valproic Acid **eFigure 3.** Flowchart of Systematic Literature Search for Lamotrigine **eFigure 4.** Flowchart of Systematic Literature Search for Carbamazepine **eTable 1.** Contact With the Authors of Trials With the Missing Data **eTable 2.** Cohort Characteristics of Included Trials on Phenytoin **eTable 3.** Cohort Characteristics of Included Trials on Valproic Acid **eTable 4.** Cohort Characteristics of Included Trials on Lamotrigine **eTable 5.** Cohort Characteristics of Included Trials on Carbamazepine **eTable 6.** Study Design of Included Trials on Phenytoin **eTable 7.** Study Design of Included Trials on Valproic Acid **eTable 8.** Study Design of Included Trials on Lamotrigine **eTable 9.** Study Design of Included Trials on Carbamazepine **eFigure 5.** Ratios of Means for Phenytoin C/D in CYP2C9 IMs Compared With CYP2C9 NMs **eFigure 6.** Ratios of Means for Phenytoin C/D in CYP2C19 IMs Compared With CYP2C19 NMs **eFigure 7.** Ratios of Means for Phenytoin C/D in CYP2C19 PMs Compared With CYP2C19 NMs **eFigure 8.** Ratios of Means for Phenytoin C/D in Combined CYP2C19 IMs and PMs Compared With CYP2C19 NMs **eFigure 9.** Ratios of Means for Valproic Acid C/D in CYP2C9 IMs Compared With CYP2C9 NMs **eFigure 10.** Ratios of Means for Valproic Acid C/D in CYP2C19 IMs Compared With CYP2C19 NMs **eFigure 11.** Ratios of Means for Valproic Acid C/D in CYP2C19 PMs Compared With CYP2C19 NMs **eFigure 12.** Ratios of Means for Valproic Acid C/D in *UGT1A6*2* Heterozygous Carriers and *2 Noncarriers **eFigure 13.** Ratios of Means for Valproic Acid C/D in *UGT1A6*2* Homozygous Carriers and *2 Noncarriers **eFigure 14.** Ratios of Means for Valproic Acid C/D in *UGT2B7*2* Heterozygous Carriers and *2 Noncarriers **eFigure 15.** Ratios of Means for Valproic Acid C/D in *UGT2B7*2* Homozygous Carriers and *2 Noncarriers **eFigure 16.** Ratios of Means for Valproic Acid C/D in *UGT2B7*3* Heterozygous Carriers and *3 Noncarriers **eFigure 17.** Ratios of Means for Valproic Acid C/D in *UGT2B7*3* Homozygous Carriers and *3 Noncarriers **eFigure 18.** Ratios of Means for Lamotrigine C/D in *UGT1A4*3* Noncarriers Compared With *UGT1A4*3* Carriers **eFigure 19.** Ratios of Means for Lamotrigine C/D in *UGT2B7*2* Heterozygous Carriers and *2 Noncarriers **eFigure 20.** Ratios of Means for Lamotrigine C/D in *UGT2B7*2* Homozygous Carriers and *2 Noncarriers **eFigure 21.** Ratios of Means for Carbamazepine Active Moiety C/D in CYP3A5 Nonexpressors and Expressors **eFigure 22.** Ratios of Means for Carbamazepine Active Moiety C/D in *EPHX1* 337CT and 337TT Carriers **eFigure 23.** Ratios of Means for Carbamazepine Active Moiety C/D in *EPHX1* 337CC and 337TT Carriers **eFigure 24.** Ratios of Means for Carbamazepine Active Moiety C/D in *EPHX1* 416AA and 416AG Carriers **eFigure 25.** Ratios of Means for Carbamazepine Active Moiety C/D in *UGT2B7*2* Heterozygous Carriers and *2 Noncarriers **eFigure 26.** Ratios of Means for Carbamazepine Active Moiety C/D in *UGT2B7*2* Homozygous Carriers and *2 Noncarriers **eFigure 27.** Funnel Plot: Small-Trial Effect for Phenytoin Meta-Analyses **eFigure 28.** Funnel Plot for Valproic Acid Meta-Analyses **eFigure 29.** Funnel Plot for Lamotrigine Meta-Analyses **eFigure 30.** Funnel Plot for Carbamazepine Meta-Analyses **eTable 10.** RoB Analysis: Phenytoin **eTable 11.** RoB Analysis: Valproic Acid **eTable 12.** RoB Analysis: Lamotrigine **eTable 13.** RoB Analysis: Carbamazepine **eTable 14.** Sensitivity Analyses: Phenytoin C/D in CYP2C9 IM vs NM **eTable 15.** Sensitivity Analyses: Phenytoin C/D in CYP2C19 IM vs NM **eTable 16.** Sensitivity Analyses: Phenytoin C/D in CYP2C19 PM vs NM **eTable 17.** Sensitivity Analyses: Valproic Acid C/D in CYP2C9 IM vs NM **eTable 18.** Sensitivity Analyses: Valproic Acid C/D in CYP2C19 IM vs NM **eTable 19.** Sensitivity Analyses: Valproic Acid C/D in CYP2C19 PM vs NM **eTable 20.** Sensitivity Analyses: Valproic Acid C/D in *UGT1A6*2* Heterozygous Carriers and Noncarriers **eTable 21.** Sensitivity Analyses: Valproic Acid C/D in *UGT1A6*2* Homozygous Carriers and Noncarriers **eTable 22.** Sensitivity Analyses: Valproic Acid C/D in *UGT2B7*3* Heterozygous Carriers and Noncarriers **eTable 23.** Sensitivity Analyses: Valproic Acid C/D in *UGT2B7*2* Heterozygous Carriers and Noncarriers **eTable 24.** Sensitivity Analyses: Valproic Acid C/D in *UGT2B7*2* Homozygous Carriers and Noncarriers **eTable 25.** Sensitivity Analyses: Lamotrigine C/D in *UGT1A4*3* Carriers and Noncarriers **eTable 26.** Sensitivity Analyses: Lamotrigine C/D in *UGT2B7*2* Heterozygous Carriers vs *2 Noncarriers **eTable 27.** Sensitivity Analyses: Carbamazepine C/D in CYP3A5 Expressors and Nonexpressors **eTable 28.** Sensitivity Analyses: Carbamazepine C/D in *EPHX1* 337TT and 337CT Carriers **eTable 29.** Sensitivity Analyses: Carbamazepine C/D in *EPHX1* 337TT and 337CC Carriers **eFigure 31.** SMD Meta-Analyses: Phenytoin C/D in CYP2C9 IMs Compared With CYP2C9 NMs **eFigure 32.** SMD Meta-Analyses: Phenytoin C/D in CYP2C19 IMs Compared With CYP2C19 NMs **eFigure 33.** SMD Meta-Analyses: Phenytoin C/D in CYP2C19 PMs Compared With CYP2C19 NMs **eFigure 34.** SMD Meta-Analyses: Phenytoin C/D in Combined CYP2C19 IMs and PMs Compared With CYP2C19 NMs **eFigure 35.** SMD Meta-Analyses: Valproic Acid C/D in CYP2C9 IMs Compared With CYP2C9 NMs **eFigure 36.** SMD Meta-Analyses: Valproic Acid C/D in CYP2C19 IMs Compared With CYP2C19 NMs **eFigure 37.** SMD Meta-Analyses: Valproic Acid C/D in CYP2C19 PMs Compared With CYP2C19 NMs **eFigure 38.** SMD Meta-Analyses: Valproic Acid C/D in *UGT1A6*2* Heterozygous Carriers and *2 Noncarriers **eFigure 39.** SMD Meta-Analyses: Valproic Acid C/D in *UGT1A6*2* Homozygous Carriers and *2 Noncarriers **eFigure 40.** SMD Meta-Analyses: Valproic Acid C/D in *UGT2B7*2* Heterozygous Carriers and *2 Noncarriers **eFigure 41.** SMD Meta-Analyses: Valproic Acid C/D in *UGT2B7*2* Homozygous Carriers and *2 Noncarriers **eFigure 42.** SMD Meta-Analyses: Valproic Acid C/D in *UGT2B7*3* Heterozygous (*3 He) Carriers and *3 Noncarriers (*3 None) **eFigure 43.** SMD Meta-Analyses: Valproic Acid C/D in *UGT2B7*3* Homozygous Carriers and *3 Noncarriers **eFigure 44.** SMD Meta-Analyses: Lamotrigine C/D in *UGT1A4*3* Noncarriers Compared With *UGT1A4*3* Carriers **eFigure 45.** SMD Meta-Analyses: Lamotrigine C/D in *UGT2B7*2* Heterozygous Carriers and *2 Noncarriers **eFigure 46.** SMD Meta-Analyses: Lamotrigine C/D in *UGT2B7*2* Homozygous Carriers and *2 Noncarriers **eFigure 47.** SMD Meta-Analyses: Carbamazepine active moiety C/D in CYP3A5 Nonexpressors and Expressors **eFigure 48.** SMD Meta-Analyses: Carbamazepine Active Moiety C/D in *EPHX1* 337CT and 337TT Carriers **eFigure 49.** SMD Meta-Analyses: Carbamazepine Active Moiety C/D in *EPHX1* 337CC and 337TT Carriers **eFigure 50.** SMD Meta-Analyses: Carbamazepine Active Moiety C/D in *EPHX1* 416AA and 416AG Carriers **eFigure 51.** SMD Meta-Analyses: Carbamazepine Active Moiety C/D in *UGT2B7*2* Heterozygous Carriers and *2 Noncarriers **eFigure 52.** SMD Meta-Analyses: Carbamazepine Active Moiety C/D in *UGT2B7*2* Homozygous Carriers and *2 Noncarriers

Supplement 2.
Data Sharing Statement
