# Supplementary material for: Pharmacogenetic Variants and Plasma Concentrations of Antiseizure Drugs: A Systematic Review and Meta-Analysis
Source: JAMA Netw Open. 2024 Aug 8;7(8):e2425593. doi: 10.1001/jamanetworkopen.2024.25593 (PMC11310823; doi:10.1001/jamanetworkopen.2024.25593)
Supplement: Supplement 1. — eAppendix. Systematic Literature Search Strategy eFigure 1. Flowchart of Systematic Literature Search for Phenytoin eFigure 2. Flowchart of Systematic Literature Search for Valproic Acid eFigure 3. Flowchart of Systematic Literature Search for Lamotrigine eFigure 4. Flowchart of Systematic Literature Search for Carbamazepine eTable 1. Contact With the Authors of Trials With the Missing Data eTable 2. Cohort Characteristics of Included Trials on Phenytoin eTable 3. Cohort Characteristics of Included Trials on Valproic Acid eTable 4. Cohort Characteristics of Included Trials on Lamotrigine eTable 5. Cohort Characteristics of Included Trials on Carbamazepine eTable 6. Study Design of Included Trials on Phenytoin eTable 7. Study Design of Included Trials on Valproic Acid eTable 8. Study Design of Included Trials on Lamotrigine eTable 9. Study Design of Included Trials on Carbamazepine eFigure 5. Ratios of Means for Phenytoin C/D in CYP2C9 IMs Compared With CYP2C9 NMs eFigure 6. Ratios of Means for Phenytoin C/D in CYP2C19 IMs Compared With CYP2C19 NMs eFigure 7. Ratios of Means for Phenytoin C/D in CYP2C19 PMs Compared With CYP2C19 NMs eFigure 8. Ratios of Means for Phenytoin C/D in Combined CYP2C19 IMs and PMs Compared With CYP2C19 NMs eFigure 9. Ratios of Means for Valproic Acid C/D in CYP2C9 IMs Compared With CYP2C9 NMs eFigure 10. Ratios of Means for Valproic Acid C/D in CYP2C19 IMs Compared With CYP2C19 NMs eFigure 11. Ratios of Means for Valproic Acid C/D in CYP2C19 PMs Compared With CYP2C19 NMs eFigure 12. Ratios of Means for Valproic Acid C/D in UGT1A6*2 Heterozygous Carriers and *2 Noncarriers eFigure 13. Ratios of Means for Valproic Acid C/D in UGT1A6*2 Homozygous Carriers and *2 Noncarriers eFigure 14. Ratios of Means for Valproic Acid C/D in UGT2B7*2 Heterozygous Carriers and *2 Noncarriers eFigure 15. Ratios of Means for Valproic Acid C/D in UGT2B7*2 Homozygous Carriers and *2 Noncarriers eFigure 16. Ratios of Means for Valproic Acid C/D in UGT2B7*3 Heteroz [file jamanetwopen-e2425593-s001.pdf]

Supplemental Online Content

Milosavljević F, Manojlović M, Matković L, et al. Pharmacogenetic variants and plasma concentrations of antiseizure drugs: a systematic review and meta-analysis. *JAMA Netw Open.* 2024;7(8):e2425593. doi:10.1001/jamanetworkopen.2024.25593

eAppendix. Systematic Literature Search Strategy
eFigure 1. Flowchart of Systematic Literature Search for Phenytoin
eFigure 2. Flowchart of Systematic Literature Search for Valproic acid
eFigure 3. Flowchart of Systematic Literature Search for Lamotrigine
eFigure 4. Flowchart of Systematic Literature Search for Carbamazepine
eTable 1. Contact With the Authors of Trials With the Missing Data
eTable 2. Cohort Characteristics of Included Trials on Phenytoin
eTable 3. Cohort Characteristics of Included Trials on Valproic Acid
eTable 4. Cohort Characteristics of Included Trials on Lamotrigine
eTable 5. Cohort Characteristics of Included Trials on Carbamazepine
eTable 6. Study Design of Included Trials on Phenytoin
eTable 7. Study Design of Included Trials on Valproic Acid
eTable 8. Study Design of Included Trials on Lamotrigine
eTable 9. Study Design of Included Trials on Carbamazepine
eFigure 5. Ratios of Means for Phenytoin C/D in CYP2C9 IMs Compared With CYP2C9 NMs
eFigure 6. Ratios of Means for Phenytoin C/D in CYP2C19 IMs Compared With CYP2C19 NMs
eFigure 7. Ratios of Means for Phenytoin C/D in CYP2C19 PMs Compared With CYP2C19 NMs
eFigure 8. Ratios of Means for Phenytoin C/D in Combined CYP2C19 IMs and PMs Compared With CYP2C19 NMs
eFigure 9. Ratios of Means for Valproic Acid C/D in CYP2C9 IMs Compared With CYP2C9 NMs
eFigure 10. Ratios of Means for Valproic Acid C/D in CYP2C19 IMs Compared With CYP2C19 NMs
eFigure 11. Ratios of Means for Valproic Acid C/D in CYP2C19 PMs Compared With CYP2C19 NMs
eFigure 12. Ratios of Means for Valproic Acid C/D in *UGT1A6*2* Heterozygous Carriers and *2 Noncarriers
eFigure 13. Ratios of Means for Valproic Acid C/D in *UGT1A6*2* Homozygous Carriers and *2 Noncarriers
eFigure 14. Ratios of Means for Valproic Acid C/D in *UGT2B7*2* Heterozygous Carriers and *2 Noncarriers
eFigure 15. Ratios of Means for Valproic Acid C/D in *UGT2B7*2* Homozygous Carriers and *2 Noncarriers

© 2024 Milosavljević F et al. *JAMA Network Open.*

eFigure 16. Ratios of Means for Valproic Acid C/D in *UGT2B7*3* Heterozygous Carriers and *3 Noncarriers
eFigure 17. Ratios of Means for Valproic Acid C/D in *UGT2B7*3* Homozygous Carriers and *3 Noncarriers
eFigure 18. Ratios of Means for Lamotrigine C/D in *UGT1A4*3* Noncarriers Compared With *UGT1A4*3* Carriers
eFigure 19. Ratios of Means for Lamotrigine C/D in *UGT2B7*2* Heterozygous Carriers and *2 Noncarriers
eFigure 20. Ratios of Means for Lamotrigine C/D in *UGT2B7*2* Homozygous Carriers and *2 Noncarriers
eFigure 21. Ratios of Means for Carbamazepine Active Moiety C/D in CYP3A5 Nonexpressors and Expressors
eFigure 22. Ratios of Means for Carbamazepine Active Moiety C/D in *EPHX1* 337CT and 337TT Carriers
eFigure 23. Ratios of Means for Carbamazepine Active Moiety C/D in *EPHX1* 337CC and 337TT Carriers
eFigure 24. Ratios of Means for Carbamazepine Active Moiety C/D in *EPHX1* 416AA and 416AG Carriers
eFigure 25. Ratios of Means for Carbamazepine Active Moiety C/D in *UGT2B7*2* Heterozygous Carriers and *2 Noncarriers
eFigure 26. Ratios of Means for Carbamazepine Active Moiety C/D in *UGT2B7*2* Homozygous Carriers and *2 Noncarriers
eFigure 27. Funnel Plot: Small-Trial Effect for Phenytoin Meta-Analyses
eFigure 28. Funnel Plot for Valproic Acid Meta-Analyses
eFigure 29. Funnel Plot for Lamotrigine Meta-Analyses
eFigure 30. Funnel Plot for Carbamazepine Meta-Analyses
eTable 10. RoB Analysis: Phenytoin
eTable 11. RoB Analysis: Valproic Acid
eTable 12. RoB Analysis: Lamotrigine
eTable 13. RoB Analysis: Carbamazepine
eTable 16. Sensitivity Analyses: Phenytoin C/D in CYP2C9 IM vs NM
eTable 15. Sensitivity Analyses: Phenytoin C/D in CYP2C19 IM vs NM
eTable 16. Sensitivity Analyses: Phenytoin C/D in CYP2C19 PM vs NM
eTable 17. Sensitivity Analyses: Valproic Acid C/D in CYP2C9 IM vs NM
eTable 18. Sensitivity Analyses: Valproic Acid C/D in CYP2C19 IM vs NM
eTable 19. Sensitivity Analyses: Valproic Acid C/D in CYP2C19 PM vs NM
eTable 20. Sensitivity Analyses: Valproic Acid C/D in *UGT1A6*2* Heterozygous Carriers and Noncarriers
eTable 21. Sensitivity Analyses: Valproic Acid C/D in *UGT1A6*2* Homozygous Carriers and Noncarriers
eTable 22. Sensitivity Analyses: Valproic Acid C/D in *UGT2B7*3* Heterozygous Carriers and Noncarriers
eTable 23. Sensitivity Analyses: Valproic Acid C/D in *UGT2B7*2* Heterozygous Carriers and Noncarriers
eTable 24. Sensitivity Analyses: Valproic Acid C/D in *UGT2B7*2* Homozygous Carriers and Noncarriers

© 2024 Milosavljević F et al. *JAMA Network Open.*

eTable 25. Sensitivity Analyses: Lamotrigine C/D in *UGT1A4*3* Carriers and Noncarriers
eTable 26. Sensitivity Analyses: Lamotrigine C/D in *UGT2B7*2* Heterozygous Carriers vs *2 Noncarriers
eTable 27. Sensitivity Analyses: Carbamazepine C/D in CYP3A5 Expressors and Nonexpressors
eTable 28. Sensitivity Analyses: Carbamazepine C/D in *EPHX1* 337TT and 337CT Carriers
eTable 29. Sensitivity Analyses: Carbamazepine C/D in *EPHX1* 337TT and 337CC Carriers
eFigure 31. SMD Meta-Analyses: Phenytoin C/D in CYP2C9 IMs Compared With CYP2C9 NMs
eFigure 32. SMD Meta-Analyses: Phenytoin C/D in CYP2C19 IMs Compared With CYP2C19 NMs
eFigure 33. SMD Meta-Analyses: Phenytoin C/D in CYP2C19 PMs Compared With CYP2C19 NMs
eFigure 34. SMD Meta-Analyses: Phenytoin C/D in Combined CYP2C19 IMs and PMs Compared With CYP2C19 NMs
eFigure 35. SMD Meta-Analyses: Valproic Acid C/D in CYP2C9 IMs Compared With CYP2C9 NMs
eFigure 36. SMD Meta-Analyses: Valproic Acid C/D in CYP2C19 IMs Compared With CYP2C19 NMs
eFigure 37. SMD Meta-Analyses: Valproic Acid C/D in CYP2C19 PMs Compared With CYP2C19 NMs
eFigure 38. SMD Meta-Analyses: Valproic Acid C/D in *UGT1A6*2* Heterozygous Carriers and *2 Noncarriers
eFigure 39. SMD Meta-Analyses: Valproic Acid C/D in *UGT1A6*2* Homozygous Carriers and *2 Noncarriers
eFigure 40. SMD Meta-Analyses: Valproic Acid C/D in *UGT2B7*2* Heterozygous Carriers and *2 Noncarriers
eFigure 41. SMD Meta-Analyses: Valproic Acid C/D in *UGT2B7*2* Homozygous Carriers and *2 Noncarriers
eFigure 42. SMD Meta-Analyses: Valproic Acid C/D in *UGT2B7*3* Heterozygous (*3 He) Carriers and *3 Noncarriers (*3 None)
eFigure 43. SMD Meta-Analyses: Valproic Acid C/D in *UGT2B7*3* Homozygous Carriers and *3 Noncarriers
eFigure 44. SMD Meta-Analyses: Lamotrigine C/D in *UGT1A4*3* Noncarriers Compared With *UGT1A4*3* Carriers
eFigure 45. SMD Meta-Analyses: Lamotrigine C/D in *UGT2B7*2* Heterozygous Carriers and *2 Noncarriers
eFigure 46. SMD Meta-Analyses: Lamotrigine C/D in *UGT2B7*2* Homozygous Carriers and *2 Noncarriers
eFigure 47. SMD Meta-Analyses: Carbamazepine active moiety C/D in CYP3A5 Nonexpressors and Expressors
eFigure 48. SMD Meta-Analyses: Carbamazepine Active Moiety C/D in *EPHX1* 337CT and 337TT Carriers

© 2024 Milosavljević F et al. *JAMA Network Open.*

eFigure 49. SMD Meta-Analyses: Carbamazepine Active Moiety C/D in *EPHX1* 337CC and 337TT Carriers
eFigure 50. SMD Meta-Analyses: Carbamazepine Active Moiety C/D in *EPHX1* 416AA and 416AG Carriers
eFigure 51. SMD Meta-Analyses: Carbamazepine Active Moiety C/D in *UGT2B7*2* Heterozygous Carriers and *2 Noncarriers
eFigure 52. SMD Meta-Analyses: Carbamazepine Active Moiety C/D in *UGT2B7*2* Homozygous Carriers and *2 Noncarriers

This supplemental material has been provided by the authors to give readers additional information about their work.

© 2024 Milosavljević F et al. *JAMA Network Open.*

1) Systematic Literature Search

1.1) Search strategy

PHENYTOIN:

MEDLINE:

> “(Diphenylhydantoin OR Dilantin OR Phenytoin OR phenytoine OR PHT) AND (CYP2C19 OR CYP2C9 OR 2c19 OR 2c9 OR pharmacogen* OR polymorph*)”

clinicaltrials.gov:

> “(Phenytoin) AND (2c19 OR 2C9 OR CYP2C19 OR CYP2C9 OR Pharmacogenomics OR Pharmacogenetics OR mutant OR variant OR gene or Genetic OR polymorphism)”

CENTRAL:

> “(Phenytoin OR phenytoine OR PHT) AND (CYP2C19 OR CYP2C9 OR 2c19 OR 2c9 OR pharmacogenomics OR polymorphism OR Variant OR gene OR genetic)”

ICTRP, EU clinical trial Register:

> “(Phenytoin OR phenytoine OR PHT OR Diphenylhydantoin OR Dilantin) AND (CYP2C19 OR CYP2C9 OR 2c19 OR 2c9 OR pharmacogenomics OR polymorphism OR Variant OR gene OR genetic)”

VALPROIC ACID:

MEDLINE, clinicaltrials.gov, CENTRAL, EU clinical trial register, ICTRP:

> “("Valproic acid" OR Valproate OR VPA OR "Propylpentanoic Acid" OR "Propylisopropylacetic Acid" OR Depakene OR Depakine OR Depakote OR "Dipropyl Acetate") AND (CYP2C19 OR CYP2C9 OR 2c9 OR 2c19 OR CYP2B6 OR 2b6 OR CYP2A6 OR 2A6 OR UGT1A6 OR UGT2B7 OR UGT1A3 OR UGT1* OR UGT2* OR "uridine diphosphate glucuronosyltransferase" OR Polymorph* OR Pharmacogen*)”

LAMOTRIGINE:

MEDLINE:

> “(Lamotrigine OR LTG) AND (UGT1a4 OR "UGT 1a4" OR UGT2b7 OR "UGT 2b7" OR UGT1a1 OR "UGT 1a1" OR "glucuronyl transferase" OR glucuronyltransferase OR UGT OR ABCB1 OR ABCG2)”

clinicaltrials.gov, EU Clinical Trial Register, ICTRP:

© 2024 Milosavljević F et al. *JAMA Network Open.*

(Lamotrigine OR LTG) AND (UGT1a4 OR "UGT 1a4" OR UGT2b7 OR "UGT 2b7" OR UGT1a1 OR "UGT 1a1" OR "glucuronyl transferase" OR glucuronyltransferase OR UGT OR Pharmacogenomics OR Pharmacogenetics OR Polymorphism OR Polymorhpic OR allele OR variant OR mutant OR SNP)

CENTRAL:

(Lamotrigine OR LTG) AND (UGT1a4 OR "UGT 1a4" OR UGT2b7 OR "UGT 2b7" OR UGT1a1 OR "UGT 1a1" OR "glucuronyl transferase" OR glucuronyltransferase OR UGT OR polymorph* OR Pharmacogen* OR variant OR allele OR SNP)

CARMBAMAZEPINE:

MEDLINE, CENTRAL, clinicaltrials.gov:

“(Carbamazepine OR CBZ) AND (CYP3a4 OR "p450 3a4" OR CYP3A5 OR "p450 3a5" OR "UGT 2b7" OR "glucuronyl transferase" OR glucuronyltransferase OR EPHX1 OR CYP2B6 OR "P450 2b6" OR CYp2C19 OR "P450 2c19") AND (polymorphism of pharmacogen* OR variant OR allele OR *1)”

ICTRP, EU Clinical Trial Register:

(Carbamazepine OR CBZ) AND (CYP3a4 OR "p450 3a4" OR CYP3A5 OR "p450 3a5" OR "UGT 2b7" OR "glucuronyl transferase" OR glucuronyltransferase OR EPHX1 OR CYP2B6 OR "P450 2b6" OR CYp2C19 OR "P450 2c19" OR Pharmacogen* OR polymorph* OR variant)

1.2) PRISMA flowcharts of systematic literature search

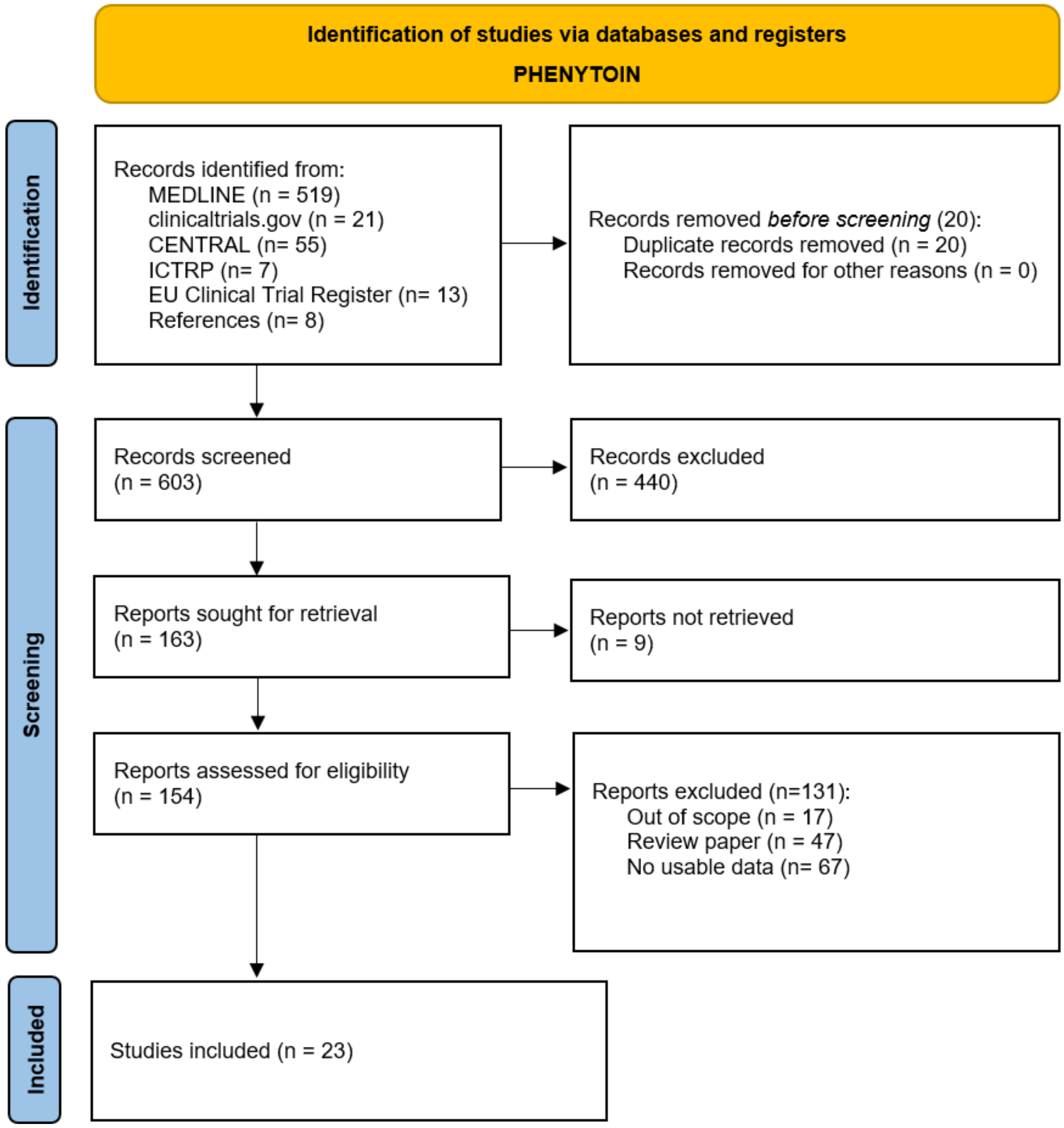

Figure S1. PRISMA flowchart: Phenytoin

© 2024 Milosavljević F et al. *JAMA Network Open.*

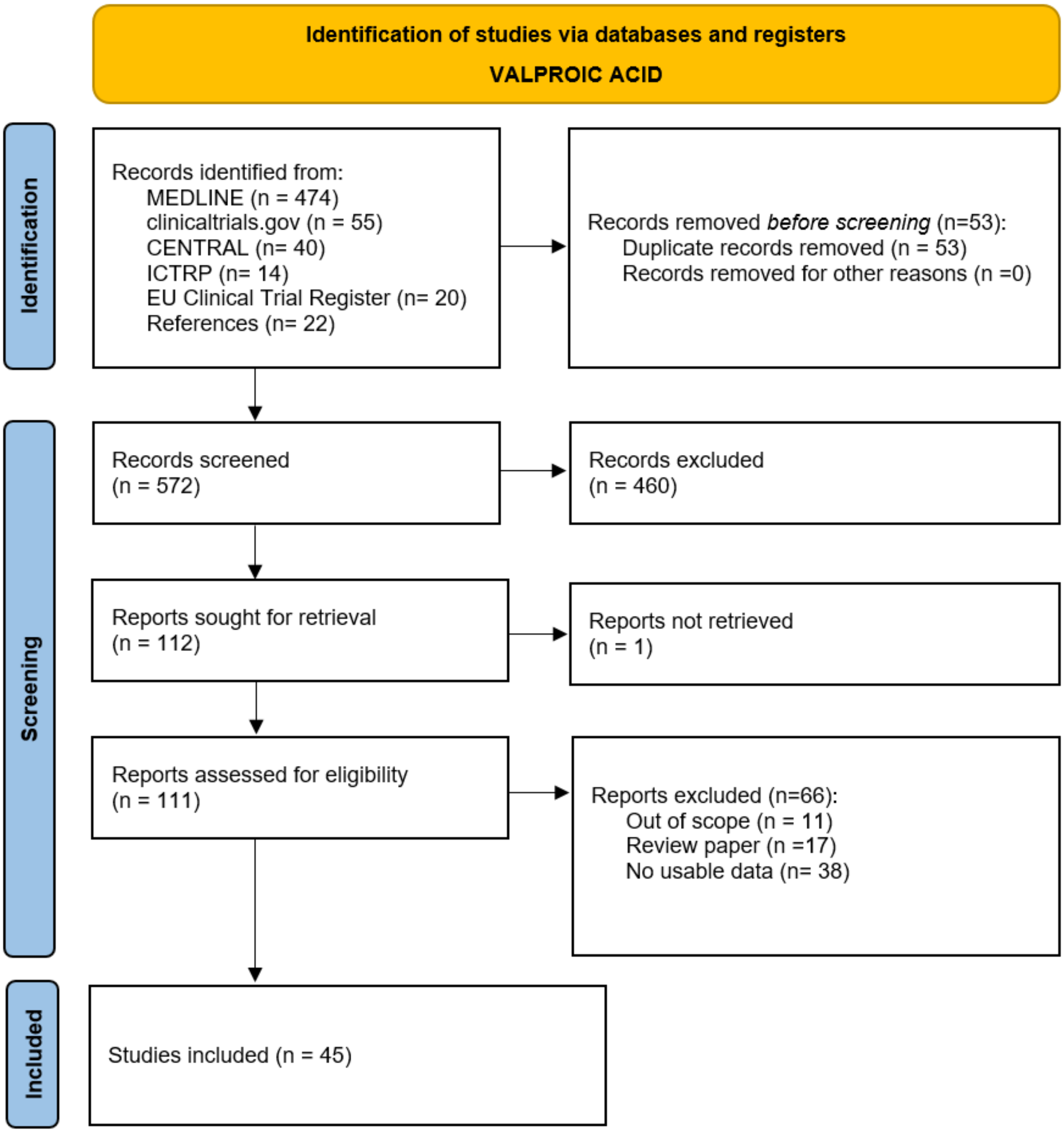

Figure S2. PRISMA flowchart: Valproic acid

© 2024 Milosavljević F et al. *JAMA Network Open.*

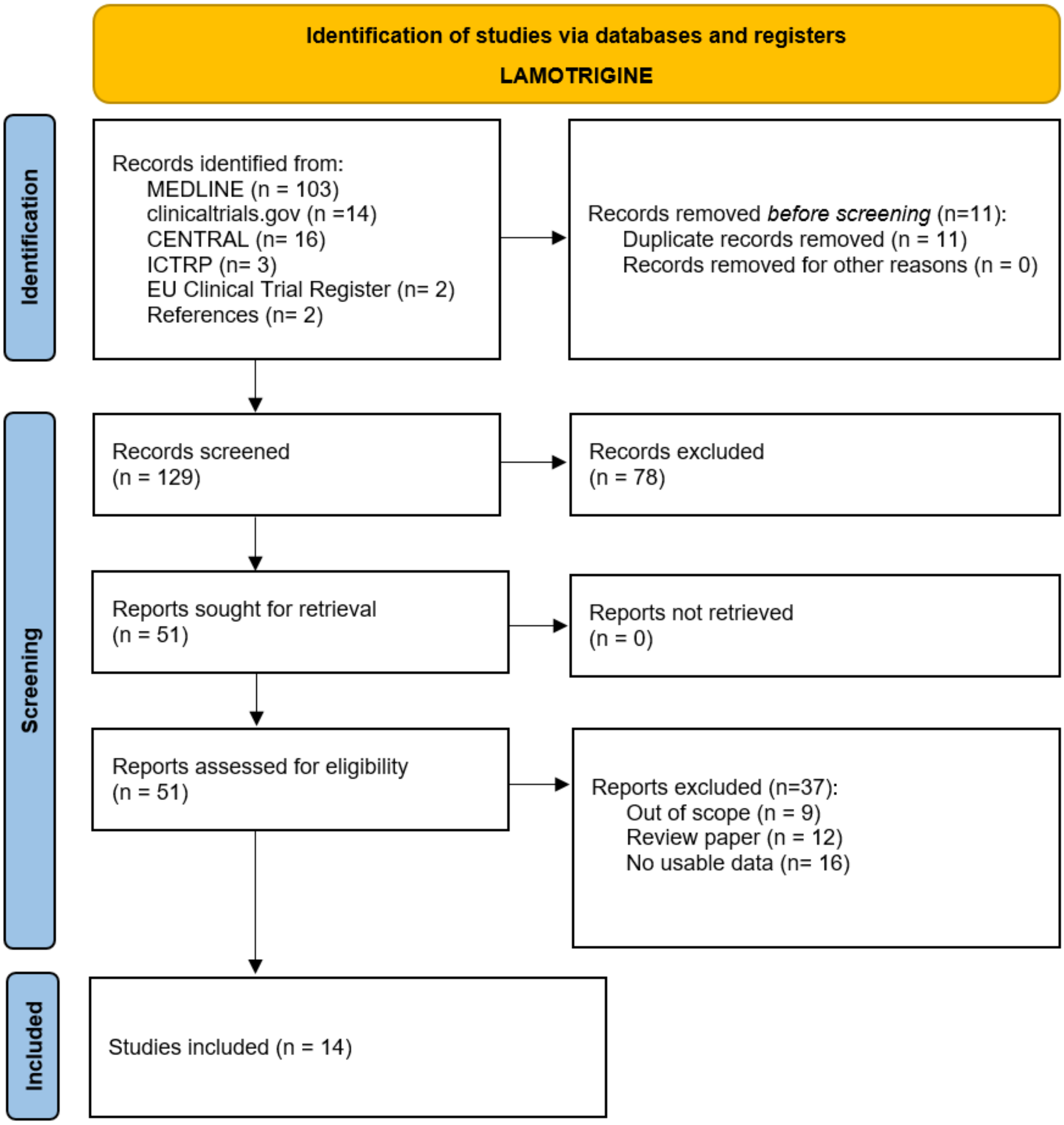

Figure S3. PRISMA flowchart: Lamotrigine

© 2024 Milosavljević F et al. *JAMA Network Open.*

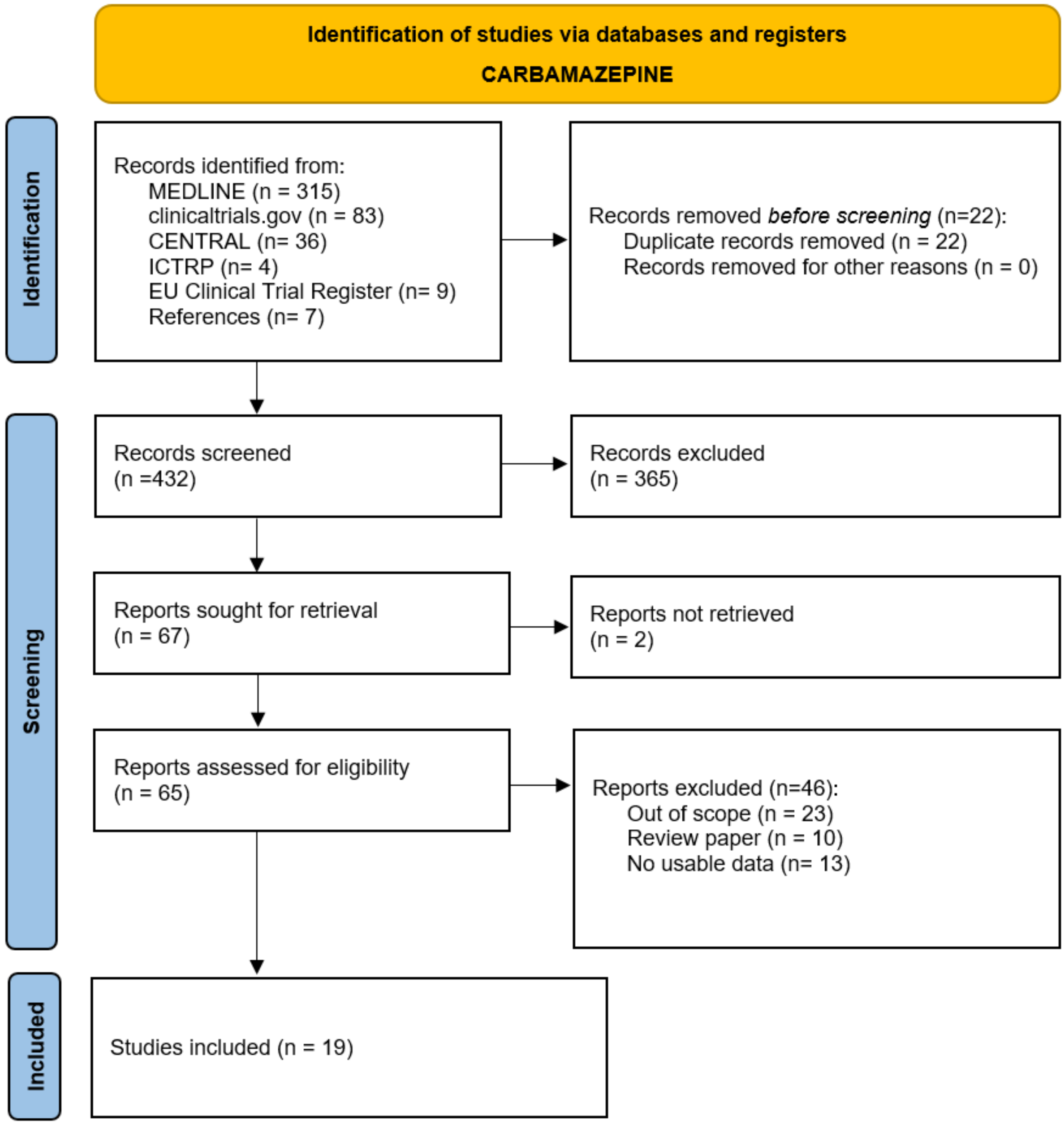

Figure S4. PRISMA flowchart: Carbamazepine

© 2024 Milosavljević F et al. *JAMA Network Open.*

1.3) Contact with the authors of trials with the missing data

Table S1: Journal of author correspondence

The paper in regard to which the correspondence was made	Date of first contact	Date of the reminder	Outcome
Ghosh 2020 (PMID: 33071782)	26. Jan 2024.	07. Feb 2024.	No response
Yampayon 2017 (PMID: 28391407)	26. Jan 2024.	07. Feb 2024.	No response
Thaker 2017 (PMID: 28302415)	26. Jan 2024.	07. Feb 2024.	No response
Chaudhary 2016 (PMID: 27179628)	26. Jan 2024.	07. Feb 2024.	No response
Ozkaynakci 2015 (PMID: 25311916)	26. Jan 2024.	07. Feb 2024.	No response
Taur 2014 (PMID: 25121365)	26. Jan 2024.	07. Feb 2024.	No response
Phabphal 2012 (PMID: 23849849)	29. Jan 2024.	07. Feb 2024.	No response
Thakkar 2012 (PMID: 23287317)	26. Jan 2024.	07. Feb 2024.	No response
Phabphal 2013 (PMID: 23159358)	29. Jan 2024.	07. Feb 2024.	No response
Chhun 2012 (PMID: 22561479)	26. Jan 2024.	07. Feb 2024.	No response
Yamamoto 2011 (PMID: 21532277)	26. Jan 2024.	07. Feb 2024.	No response
Kesavan 2010 (PMID: 20390258)	29. Jan 2024.	07. Feb 2024.	Response on 7. Feb 2024. Authors provided needed clarification.
Allabi 2005 (PMID: 16220110)	26. Jan 2024.	N/A	Unable to contact (inactive email address)
Lim 2004 (PMID: 15247556)	26. Jan 2024.	07. Feb 2024.	No response
NCT00162461	26. Jan 2024.	07. Feb 2024.	No response
Odani 1997 (PMID: 9333104)	26. Jan 2024.	07. Feb 2024.	No response
Yukawa 2006 (PMID: 16789993)	26. Jan 2024.	N/A	Unable to contact (inactive email address)
Božina 2023 (PMID: 37340142)	26. Jan 2024.	07. Feb 2024.	Response on: 29. Jan 2024. No new data could be included.
Zhu 2022 (PMID: 36324679)	26. Jan 2024.	07. Feb 2024.	No response
Adiga 2022 (PMID: 36160414)	26. Jan 2024.	07. Feb 2024.	No response
Sanguesa 2022 (PMID: 35853541)	26. Jan 2024.	07. Feb 2024.	Response on 07. Feb 2024. Provided data did not fulfil inclusion criteria
Mani 2022 (PMID: 34656038)	26. Jan 2024.	07. Feb 2024.	No response
Xu 2018 (PMID: 29981400)	26. Jan 2024.	07. Feb 2024.	No response
Kumar 2011 (PMID: 21747585)	26. Jan 2024.	07. Feb 2024.	No response
Li 2016 (PMID: 27918244)	26. Jan 2024.	07. Feb 2024.	No response
Amini-Shirazi 2010 (PMID: 20602621)	26. Jan 2024.	07. Feb 2024.	No response
Liu 2015 (PMID: 26303110)	26. Jan 2024.	07. Feb 2024.	No response
Inoue 2016 (PMID: 26790665)	26. Jan 2024.	07. Feb 2024.	No response
Nakajima 2005 (PMID: 15692831)	26. Jan 2024.	07. Feb 2024.	No response
Puranik 2013 (PMID: 23252947)	26. Jan 2024.	07. Feb 2024.	No response
Caruso 2014 (PMID: 24817818)	26. Jan 2024.	07. Feb 2024.	No response

© 2024 Milosavljević F et al. *JAMA Network Open.*

2) Characteristics of the included trials

Table S2: Cohort characteristics of included trials on Phenytoin: Table summarizes the demographic data, diagnosis and inclusion/exclusion criteria for clinical trials included in any phenytoin related meta-analysis performed. In case that only a subset of the cohort could be included in meta-analysis, only the characteristics of that specific subset are presented. Cohort sizes are presented in Mean ± Sd and/or (Range). Ethnicity was reported as specified in the given manuscript. **: Estimated; NI: No information; ASD: Anti-seizure drug;*

Trial	Sample size	% of females	Age category	Average age	Ethnicity	Diagnosis	Inclusion criteria	Exclusion criteria
Shaul 2022	n= 150	40%	Adults	25 ± 5	Caucasian	Healthy	Healthy subjects	Smokers; Taking any medication regularly
Wanounou 2022	n= 143	57%	Adults	25 ± 5	Ethiopian Jewish	Healthy	Aged 18 - 50 years; In good health	Smokers; Chronic diseases; Taking any other drug, oral contraceptives or alcohol regularly
Fohner 2019	n= 319	52%*	Adults	62 ± 15*	5% Black 8% White Hispanic, 82% White non-Hispanic,	Seizures, brain tumors etc.	Filling at least one prescription for phenytoin between 1996 and 2017	Not alive at the time of data synthesis; Left program within 30 days of initiating therapy; Taking other ASDs within 180 days
Guevara 2017	n= 50	50%	Adults	(18 – 76)	Caucasian (Uruguayan)	Epilepsy	Epileptic patients; Aged 18 - 76 years; Chronic treatment with oral phenytoin	Hepatic or renal impairment
Li Z 2016	n= 62	44%	Adults	(23 – 76)	Chinese	Intracranial space lesions (Various)	Received oral phenytoin to prevent post-craniotomy epileptic seizures for over 7 days	Serious hepatic or renal impairment; Low compliance; Oral phenytoin therapy before operation
Ortega-Vázquez 2015	n= 64	31%	Adults and Adolescents	(16 – 65)	Mexican Mestizo	Epilepsy	Adults; Epilepsy currently treated with 100 - 600 mg of phenytoin; Over 16 years old	NI
Sharma 2015	n= 35	22%*	Adults and Adolescents	22 ± 9*	Northern Indian	Epilepsy	Epilepsy diagnosis; Aged 12 - 40 years old; Receiving monotherapy of first-line ASDs	Gross neurological deficits; Severe hepatic or renal disorders, or diabetes mellitus; History of smoking or drug abuse; Pregnancy
Yamamoto 2015	n= 170	38%	Children	8 ± 4	Japanese	Epilepsy	Pediatric epilepsy patients	Malnutrition; Hepatic dysfunction

© 2024 Milosavljević F et al. *JAMA Network Open.*

Table S2. Continued

Trial	Sample size	% of females	Age category	Average age	Ethnicity	Diagnosis	Inclusion criteria	Exclusion criteria
George 2012	n= 48	NI	Adults	35 ± 3	Tamilian (India)	Tuberculosis	Unrelated; Newly diagnosed tuberculosis; Aged >18 years; Tamil Nadu and Pondicherry heritage; Tamil as mother tongue	Severe illnesses which warrant hospitalization; Pregnancy; Taking medication that induce or inhibit CYP2C9 enzyme
Hung 2012	n= 269	38%	Adults	42 ± 1	Han Chinese	Epilepsy	Epilepsy patients; Phenytoin monotherapy; Achieved maintenance dose ≥1 year ago; Good seizure control	Taking other ASDs; Taking non-ASDs that interacts with phenytoin
Kesavan 2010	n= 292	32%	Adults	30 ± 11	Tamilian (India)	Epilepsy	Taking oral phenytoin >2 month; On stable drug regimen	Hepatic or renal failure
Lin 2008	n= 53	48%	Adults	38 ± 10	Taiwanese*	Epilepsy	Aged ≥18 years; Taking oral phenytoin >1 year; Not changing drugs or doses 3 months before the study	Allergic to galactose; Pregnant; History of severe hepatic or renal failure
Lee 2007	n= 97	53%	Adults and Adolescents	(17 – 79)	Korean	Epilepsy	Oral phenytoin therapy	No hepatic or renal dysfunction
Rosemary 2006	n= 27	37%	Adults	26 ± 2	Tamilian (Indian)	Healthy	Individuals known from a previous study; Available in Pondicherry; Unrelated	NI
Yamanaka 2005	n= 15	33%	Adults and Adolescents	38 ± 26	Japanese	Post-surgery prophylaxis	NI	Abnormal renal and hepatic functions
Taguchi 2005	n= 20	NI	Children, Adolescents and Adults	12 ± 5	Japanese	Epilepsy	Routinely taking oral Phenytoin	Renal or liver failure
Huang 2004	n= 32	NI	Children, Adolescents and Adults	(2 – 68)	Chinese	Epilepsy	Phenytoin monotherapy; Idiopathic epilepsy diagnosis	Renal of hepatic dysfunction; Taking co-medications

© 2024 Milosavljević F et al. *JAMA Network Open.*

Table S2. Continued

Trial	Sample size	% of females	Age category	Average age	Ethnicity	Diagnosis	Inclusion criteria	Exclusion criteria
Hung 2004	n= 169	43%	Adults	38 ± 13	Taiwanese	Epilepsy	Taking oral phenytoin; Same dose of phenytoin and co-medications >1 month before the study	Abnormal liver and kidney function
Soga 2004	n= 28	29%	Adults and Adolescents	(15 – 75)	Japanese	Epilepsy	Age 15 - 75 years; Taking almost stable dose of phenytoin ≥5 years	NI
Caraco 2001	n= 31	29%	Adults	30 ± 2	Caucasian	Healthy	Healthy volunteers	Taking any medication or alcohol 1 week before and 1 week after the study; Smokers
van der Weide 2001	n= 60	37%	Adults and Adolescents	(16 – 74)	Dutch	Epilepsy in intellectually disabled	On phenytoin ≥6 months; Stable drug regimen for 6 months	NI
Aynacioglu 1999	n= 101	NI	Adults*	NI	Turkish	Healthy	Healthy volunteers	NI
Mamiya 1998	n= 134	40%	Adults	47 ± 13	Japanese	Epilepsy	Oral administration of tablet or granule of phenytoin; Japanese adults	Hepatic or renal failure; Changes in phenytoin doses or co-medications 1 month before the trial

© 2024 Milosavljević F et al. *JAMA Network Open.*

Table S3. Cohort characteristics of included trials on Valproic acid: Table summarizes the demographic data, diagnosis and inclusion/exclusion criteria for clinical trials included in any valproic acid related meta-analysis performed. If only a subset of the cohort could be included in meta-analysis, only characteristic of that specific subset are presented. Cohort sizes are presented in Mean ± Sd and/or (Range). Ethnicity was reported as specified in the given manuscript. *[a]: Only unique patients are presented and analyzed, even though the manuscript describes some number of patients that overlap with other manuscripts; NI: No information; ASD: Anti-seizure drug; *: Estimated; ER: Extended release; VPA: Valproic acid; TDM: Therapeutic drug monitoring; EEG: Electroencephalography; ILAE: International League Against Epilepsy diagnosis criteria; ECG: Electrocardiography; CYP: CYP450 enzymes; UGT: Uridine 5'diphospho-glucuronosyltransferase enzymes.*

**Trial**	**Sample**	**% of females**	**Age category**	**Average age**	**Ethnicity**	**Diagnosis**	**Inclusion criteria**	**Exclusion criteria**
Zheng 2023	n= 46	41%	Adults	66 ± 17	Han Chinese	Epilepsy	Generalized or partial seizures; Taking injections, tablets and ER tablets VPA; Treated for a long time	Severe renal or liver impairment; Serious adverse drug reactions; Missed follow-up
Wang 2021	n= 296	74%	Adults	35 ± 12	Han Chinese	Schizophrenia	18 – 60 years old; Schizophrenia (ICD-10) diagnosis; Han Chinese origin; Inpatients	Liver or renal impairment; Substance abuse; Treatment-resistant schizophrenia; Pregnant or lactating women; Severe physical disease or brain organic diseases; Taking oral contraceptives
Du 2021	n= 229	35%	Children	5 ± 4	Wuhan Chinese	Epilepsy	Epilepsy (ILAE) diagnosis; Regularly taking VPA for ≥4 weeks	Hepatic or Renal impairment; Poor compliance; Severe adverse drug reactions; Taking drugs than may impact VPA pharmacokinetics
Wu 2021	n= 112	30%	Children	6 ± 4	Han Chinese	Various diagnoses	Diagnosis of epilepsy, infectious diseases, neurodevelopmental disorders, or other; Age ≤ 14 years; Single or combined treatment with oral VPA; TDM recommended	Continuous treatment with VPA lasted <72h; Drug non-adherence within 72h before TDM

© 2024 Milosavljević F et al. *JAMA Network Open.*

Table S3. Continued

Trial	Sample	% of females	Age category	Average age	Ethnicity	Diagnosis	Inclusion criteria	Exclusion criteria
Xu 2021	n= 194	34%	Children	(1 – 14)	Chinese	Epilepsy	Pediatric epilepsy patients; VPA plasma levels were 20–50 µg/ml or 100–150 µg/ml	Liver or kidney disfunction; VPA plasma levels <20µg/ml or >150µg/ml due to the risk of non-compliance or laboratory error
Nandith 2021	n= 99	43%	Children	8 ± 4	South Indian	Epilepsy	Aged 2–18 years; Either sex; Diagnosed by EEG; On steady stable dose of 20 mg/kg/d VPA monotherapy twice or thrice a day for past one month	History of hepatitis renal impairment; Taking any other ASD or a drug which induce or inhibit enzymes involved in VPA metabolism
Song 2020	n= 83	30%	Adults	50 ± 19	Yunnan province, China (diverse)	Seizures	Diagnosis of seizures; Age 18 or older; Regularly taking sustained-release valproate twice daily for at least 1 month	Taking other ASDs; Serious liver, renal or cardiac diseases; Poor adherence; Serious adverse drug reactions to VPA
Zhao 2020	n= 122	49%	Children	5 ± 4	Chinese	Epilepsy	Children; Taking VPA monotherapy for ≥3 months; Achieved steady state	Hepatic or renal dysfunction
Wang 2019	n= 157	37%	Children	6 ± 4	Chinese	Epilepsy	Epilepsy or an epileptic syndrome diagnosis; Taking VPA for ≥2 years	Hepatic or renal dysfunction
Feng 2019	n= 29[a]	34%	Children and Adolescents	10 ± 3	Chinese	Epilepsy	Focal seizure diagnosis; Aged 2 - 18 years; Taking VPA; Regularly taking VPA >30 days;	Other serious diseases; Serious renal and liver impairment; Failure to obtain efficacy data
Feng 2018	n= 174	39%	Children	9 ± 4	Chinese	Generalized seizures	Generalized seizure diagnosis; Age 2 -18 years; Taking VPA	Other serious disease; Severe renal, liver, or cardiac impairment; Serious adverse drug reactions; Loss to follow-up

© 2024 Milosavljević F et al. *JAMA Network Open.*

Table S3. Continued

Trial	Sample	% of females	Age category	Average age	Ethnicity	Diagnosis	Inclusion criteria	Exclusion criteria
Zhang 2017	n= 177	39%	Children, Adolescent and Adults	16 ± 11	Chinese	Epilepsy	Epilepsy diagnosis; Taking VPA for >1 month; Good compliance	Serious liver and kidney dysfunction; Significant side effects; No comedications
Sun 2017	n= 395	32%	Adults	47 ± 18	Chinese	Severe brain traumatic injury	Adult patients (age ≥ 16); Severe blunt and isolated Traumatic brain injury	Body weight > 90kg or <40kg; Pregnant; Preexisting seizures; History of Cirrhosis or renal failure; VPA allergy; Patients unlikely to survive 3 days; Taking drugs likely to interact with VPA for 7 days
Mei 2017	n= 47[a]	32%	Children	10 ± 3	Chinese	Epilepsy	Epilepsy diagnosis; Aged 2 - 18 years; Regularly taking sustained-release tablets of VPA twice a day for ≥ 30 days	Severe liver, renal or cardiac impairment; Serious adverse drug reaction to VPA; Taking other ASDs; Other serious diseases
Li Z 2017	n= 84	44%	Adults	Mostly >65	Chinese	Craniotomy	Aged 16 - 80 years old; Taking VPA immediately after the surgery; Undergone the treatment with a lumbar puncture	Agonal state; Deep coma; Absence of spontaneous breathing; Severe dysfunction of the heart, lung, liver, kidney, or other organs; Pregnant
Wang 2017	n= 102	37%	Children	5 ± 2	Han Chinese	Epilepsy	Han Chinese; Taking oral solution of VPA for ≥1 month as monotherapy; Completed the registration of clinical data	Abnormal liver or kidney function; Encephalitis or other nervous system diseases
Zhao 2017	n= 200	NI	Adolescents and Adults	15 ± 18	Chinese	Epilepsy	Epilepsy; Taking VPA monotherapy for ≥3 months	Abnormal liver function

© 2024 Milosavljević F et al. *JAMA Network Open.*

Table S3. Continued

Trial	Sample	% of females	Age category	Average age	Ethnicity	Diagnosis	Inclusion criteria	Exclusion criteria
Jogamoto 2017	n= 28	64%	Children, Adolescents and Adults	9 ± 10	Japanese	Dravet syndrome	Dravet syndrome; Taking Stiripentol and VPA combination therapy	NI
Algharably 2016	n= 48	58%	Children and Adolescents	9 ± 4	Egyptian	Epilepsy	Aged 2-18 years; Idiopathic epilepsy diagnosis (ILAE); Taking VPA monotherapy	Hepatitis; Unreliable record of seizure Frequency; History of noncompliance; Taking drugs that interfere with enzymes important for VPA; Neurodegenerative, Neurometabolic or neurocutaneous syndromes
Kang 2016	n= 129	36%	Children, Adolescents and Adults	18 ± 26	Chinese	Epilepsy	VPA monotherapy for at least 5 half-lives	Liver or kidney abnormalities
Feng 2016	n= 58[a]	45%	Children	4 ± 3	Chinese	Epilepsy	Aged 3 months to 18 years; Epilepsy diagnosis; Taking VPA oral solution regularly for ≥30 days; No blood transfusion for the 3 months before blood samples were taken	Severe cardiac, liver, or renal dysfunction; Adverse drug reactions to VPA; Any other circumstance considered unsuitable for enrollment
Du 2016	n= 102	67%	Children and Adolescents	12 ± 8	Chinese	Epilepsy	Pediatric patients; Epilepsy diagnosis based on the seizure history and bio-chemical laboratory and EEG tests	Abnormal hepatic or renal function
Chatzistefanidis 2016 [Adults]	n= 76	42%	Adolescents and Adults	37 ± 16	Greek	Epilepsy	Outpatients; Taking VPA either as mono- or a polytherapy	Liver or renal disease or biochemical markers suggesting it; Any disease affecting drug absorption; Noncompliance; Taking medication possibly interacting with VPA metabolism
Chatzistefanidis 2016 [Children]	n= 58	43%	Children	8 ± 4				

© 2024 Milosavljević F et al. *JAMA Network Open.*

Table S3. Continued

Trial	Sample	% of females	Age category	Average age	Ethnicity	Diagnosis	Inclusion criteria	Exclusion criteria
Shen 2016	n= 97	38%	Adolescent and Adults	32 ± 19	South Chinese	Epilepsy	Partial or generalized seizures diagnosis; Taking VPA as monotherapy for >1 month	Liver or kidney disfunction
Wang 2016	n= 166	40%	Children and Adolescents	9 ± 4	Chinese	Epilepsy	Different types of epilepsy; Taking VPA and lamotrigine combination for ≥6 months	Taking drugs known to influence the pharmacokinetics of VPA and/or Lamotrigine; Liver or renal disorders
Wen 2016	n= 149	NI	Children	13 ± 16	Chinese	Epilepsy	Definitive diagnosis of epilepsy by neurologist; Taking VPA as monotherapy twice daily for ≥2 weeks	Taking any CYP or UGT inducers or inhibitors; Abnormal liver or renal function; Irregular use of VPA
Smith 2016	n= 252	55%	Adults	Mostly >65	Norwegian	Mostly Psychiatric patients	Gave blood samples 10–26 h after the last dose; Requisition forms provided data on dosage, and serum concentrations; Likely achieved steady-state; Serum concentrations above the lower limit of quantification	Taking carbamazepine, phenobarbital or phenytoin
Sun 2015	n= 102	48%	Adults	31 ± 10	Chinese	Epilepsy	Outpatients, Epilepsy diagnosis based on their seizure history and the results of EEG and biochemical laboratory tests	Liver and kidney disfunction
Han 2015	n= 177	NI	NI	NI	Chinese	Epilepsy	Inpatients	NI
Tóth 2015	n= 50	60%	Children and Adolescents	7 ± 3	Caucasian	Epilepsy	Novel epileptic patients; Younger than 15 years; Were CYP2C9 tested at the beginning of antiseizure therapy	Taking polytherapy; Having VPA therapy interrupted

© 2024 Milosavljević F et al. *JAMA Network Open.*

Table S3. Continued

Trial	Sample	% of females	Age category	Average age	Ethnicity	Diagnosis	Inclusion criteria	Exclusion criteria
Jain 2015	n= 80	32%	Children	8 ± 3	North Indian	Epilepsy	Aged 3-12 years; Epilepsy diagnosis; Taking VPA monotherapy (plain tablets) in a stable dose for ≥1 month	Taking drugs which may interfere with VPA metabolism; Taking herbal or alternative medicine; Inadequate compliance; Chronic hepatic, renal, or cardiac disease
Aphichartphunkawee 2014	n= 84	44%	Adolescents and Adults	41 ± 14	Thai	Epilepsy	Epilepsy diagnosis; Taking VPA alone or with other ASDs to control epilepsy; Taking VPA for ≥1 month to ensure steady-state is achieved	Liver or kidney dysfunction; History of non-compliance; Unclear medical history; Drug or alcohol abuse; Psychological disorders; Pregnant or breast feeding
Inoue 2014	n= 78	51%	Children and Adolescents	6 ± 4	Japanese	Epilepsy	Pediatric epilepsy patients; Both inpatients and outpatients; Carrying the CYP2C9*1/*1 genotype	Carrying the CYP2C9*3 allele; Taking CYP and UGT enzyme inducers
Liao 2013	n=115	44%	Children	5 ± 4	Chinese	Epilepsy	Blood VPA level within 50-100 mg/ml; Free of seizures and no dose change for ≥6 months; Taking VPA monotherapy for ≥5 half- lives; Outpatients	Abnormalities of liver or renal function; Other serious central nervous system disorders
Ma 2013	n= 248	33%	Children, Adolescents and Adults	(0 – 59)	Chinese	Epilepsy	Taking VPA for at least 1 month	Abnormal liver function
Rutigliano 2013	n= 69	38%	Adult	NI	Caucasian*	Bipolar disorder	Both in- and outpatients	NI
Jin 2013	n= 97	41%	Children, Adolescents and Adults	23 ± 20	Chinese	Epilepsy	TDM performed; Taking VPA as monotherapy	Abnormal liver or kidney function
Guo 2012	n= 98	43%	Children and Adolescents	8 ± 8	Chinese	Epilepsy	Diagnosis of partial or generalized seizures; Taking VPA as monotherapy for ≥1 month	Taking drugs known to modulate activities of UGT1A6, UGT2B7 and CYP2C9; Renal or liver disorders

© 2024 Milosavljević F et al. *JAMA Network Open.*

Table S3. Continued

Trial	Sample	% of females	Age category	Average age	Ethnicity	Diagnosis	Inclusion criteria	Exclusion criteria
Chu 2012	n= 242	33%	Adults	30 ± 16	Chinese	Epilepsy	Outpatients; Epilepsy diagnosis based on their seizure history and the result of EEG and biochemical laboratory tests	Abnormal liver and kidney function
Sterjev 2012	n= 153	NI	NI	NI	Caucasian*	Epilepsy	NI	NI
Hung 2011	n= 162	44%	Adults	36 ± 1	Chinese	Epilepsy	Taking VPA monotherapy; Reached a maintenance dose for ≥1 year; Good compliance and seizure control; Have had EEG and MRI brain scans	Taking drugs that may interact with VPA
Wang 2010	n= 147	NI	Children and Adolescents	(3 – 14)	Han Chinese	Epilepsy	Taking VPA as monotherapy; Epilepsy diagnosis (ILAE)	Hepatic or renal impairment; Encephalopathy; Other central nervous system disorders
Tan 2010	n= 179	47%	Children, Adolescents and Adults	24 ± 3	Han Chinese	Epilepsy	Outpatients; Taking VPA monotherapy for ≥6 months; On a stable dosage regimen for ≥5 half-life periods	Abnormal findings for hepatic and renal function tests
Chung 2008	n= 14	57%	Adults	23 ± 2	Korean	Healthy	Volunteers; Carriers of UGT2B15 *2/*2 genotype; Health ascertained by medical history, a physical examination, vital signs, 12-lead ECG, and routine clinical laboratory tests performed within a period of 3 weeks before the study start	Heavy drinkers; Smokers of more than 10 cigarettes per day; Body weight more than 20% of their ideal weight; Pregnancy; Drug abuse; Taking caffeine, medications, herbal drugs
Sun 2007	n= 69	48%	Children, Adolescents and Adults	(5 – 51)	Chinese	Epilepsy	Taking VPA between 5 days - 6 years as monotherapy	Liver of kidney abnormalities

© 2024 Milosavljević F et al. *JAMA Network Open.*

Table S4. Cohort characteristics of included trials on Lamotrigine: Table summarizes the demographic data, diagnosis and inclusion/exclusion criteria for clinical trials included in any Lamotrigine related meta-analysis performed. If only a subset of the cohort could be included in meta-analysis, only characteristic of that specific subset are presented. Cohort sizes are presented in Mean ± Sd and/or (Range). Ethnicity was reported as specified in the given manuscript. **: Estimated; NI: No information; ASD: Anti-sizure drug; LTG: Lamotrigine; VPA: Valproic acid; EEG: Electroencephalogram; ECG: Electrocardiogram; TDM: therapeutic drug monitoring*

**Trial**	**Sample**	**% of females**	**Age category**	**Average age**	**Ethnicity**	**Diagnosis**	**Inclusion criteria**	**Exclusion criteria**
Božina 2023	n= 471	60%	Adults and Adolescents	39 ± 15	Croatian (Caucasian)	Epilepsy	Epilepsy diagnosis; Taking LTG monotherapy or LTG + VPA therapy; Aged ≥ 16 years	Active or ex-smokers; Taking other ASDs or drugs known to affect LTG or VPA within the previous month; Cardiac, renal, thyroid or liver disfunction; Diabetes mellitus; Previous or ongoing malignant disease; Any acute illness; Pregnant women; HIV infection
Petrenaite 2022	n= 198	58%	Adults	(18 – 67)	Danish (Caucasian)	Epilepsy	Caucasian; Epilepsy diagnosis; Taking LTG, Aged 18-65 years	Non-adherence
Ortega-Vázquez 2020	n= 97	51%	Adults and Adolescents	32 ± 13	Mexican Mestizo	Epilepsy	Epilepsy diagnosis; Aged 15–73 years; Taking standard LTG daily dose (50–400 mg)	NI
Suzuki 2019 (Monotherapy)	n= 67	NI	Adults	42 ± 16	Japanese	Major depressive disorder or bipolar disorder	Japanese; Inpatients or outpatients; Major depressive disorder or Bipolar disorder I and II diagnosis; Physically healthy	History of substance abuse including alcoholism; Neurological disorder, delirium, or dementia; Clinically significant findings via laboratory examinations, ECG or EEG; Females taking oral contraceptives
Suzuki 2019 (+Valproic acid)	n= 36							
Petrenaite 2018	n= 33	100%	Adults	(21 – 43)	Danish	Epilepsy	Danish women; Epilepsy diagnosis; Outpatients	Smokers; Taking co-medication that could potentially influence LTG metabolism

© 2024 Milosavljević F et al. *JAMA Network Open.*

Table S4. Continued

Trial	Sample	% of females	Age category	Average age	Ethnicity	Diagnosis	Inclusion criteria	Exclusion criteria
Smith 2018	n= 534	69%	Adults	48 ± 13	Norwegian*	Epilepsy and Bipolar disorder	Available UGT1A4 genotype and LTG TDM information; Serum samples drawn 10–30h after the last dose intake; Valid information on LTG dosing provided	LTG levels under the lower limit of quantification; Patient aged <18 years; Written requisition reports pregnancy or the use of the enzyme inducers (carbamazepine, phenytoin, and phenobarbital)
He 2017	n= 72	33%	Children	9 ± 2	Southern Han Chinese	Epilepsy	Epilepsy diagnosis (Based on Zhufutang Practical Pediatrics); Aged 0-18; Complete medical history available	Abnormal hepatic and renal function; Serious brain lesions; Irregular treatment; Serious gastrointestinal diseases; Other serious illnesses; Taking blood transfusion; Poor adherence
Du 2016	n= 102	44%	Children and Adolescents	12 ± 6	Qingzhou district, Chinese	Epilepsy	Children; Epilepsy diagnosis based on the seizure history and biochemical laboratory and EEG results	Abnormal hepatic and renal function
Reimers 2016	n= 178	63%	Children, Adolescents and Adults	(6 – 78)	Caucasian	NI	Gave blood for the preceding clinical study; Taking LTG	Non-adherence
Wang 2016	n= 166	40%	Children and Adolescents	9 ± 4	Chinese	Epilepsy	Chinese children; Diagnosis of different types of epilepsy; Taking VPA + LTG for ≥6 months	Taking drugs known to influence the pharmacokinetics of VPA and/or LTG; Liver or renal disorders
Liu 2015 (Monotherapy)	n= 56	45%	Children and Adolescents	8 ± 3	Chinese	Epilepsy	Epilepsy diagnosis; Aged 2 - 18 years; Taking LTG + VPA for ≥1 month to ensure a steady-state	Hepatic or renal disfunction
Liu 2015 (+Valproic acid)	n= 158	39%		9 ± 4				
Wang 2015	n= 148	41%	Children and Adolescents	9 ± 4	Chinese	Epilepsy	Chinese children; Diagnosis of different types of epilepsy; Taking VPA + LTG for ≥6 months	Liver or renal disorders

© 2024 Milosavljević F et al. *JAMA Network Open.*

Table S4. Continued

Trial	Sample	% of females	Age category	Average age	Ethnicity	Diagnosis	Inclusion criteria	Exclusion criteria
Zhou 2015	n= 140	54%	Adults and Adolescents	30 ± 13	Han Chinese	Epilepsy	Aged 12 - 65 years; Epilepsy diagnosis; Taking LTG monotherapy without dose interruption for ≥1 month; Complete medical records;	Abnormal liver or kidney function; Poor compliance; Taking oral contraceptives; Pregnant
Chang 2014	n= 106	59%	Adults and Adolescents	45 ± 12	Han Chinese	Epilepsy	Outpatients and inpatients; Aged ≥14 years; Diagnosis of epilepsy; Taking LTG therapy for >1 month; Treatment compliant; Seizure frequency of ≥1 per month for 3 months before the therapy	Taking drugs that influence the liver enzyme activity; Kidney or liver disease; Abnormal laboratory results; Pregnant or breast feeding; Severe organ disease; Diseases known to influence the evaluation of efficacy and safety of LTG; Allergy to the study drug

© 2024 Milosavljević F et al. *JAMA Network Open.*

Table S5. Cohort characteristics of included trials on Carbamazepine: Table summarizes the demographic data, diagnosis and inclusion/exclusion criteria for clinical trials included in any valproic acid related meta-analysis performed. If only a subset of the cohort could be included in meta-analysis, only characteristic of that specific subset are presented. Cohort sizes are presented in Mean ± Sd and/or (Range). Ethnicity was reported as specified in the given manuscript. *NI: No information; ASD: Anti-seizure drug; CBZ: Carbamazepine; ADR: Adverse drug reaction;*

**Trial**	**Sample**	**% of females**	**Age category**	**Average age**	**Ethnicity**	**Diagnosis**	**Inclusion criteria**	**Exclusion criteria**
Venkatraman 2023	n= 115	44%	Adults	30 ± 11	South Indian	Epilepsy	Epilepsy diagnosis; Seizure free for 1 year; Dose was not changed for ≥1 year	Liver or kidney dysfunction; Pregnant or lactating women
Pham 2020	n= 38	42%	Adults	51 ± 13	Vietnamese	Epilepsy	Epilepsy diagnosis; Taking the same dose of CBZ for >1 month	Pregnant or breastfeeding women; Alcohol use; Co-medication known to affect liver enzyme activity; Liver or renal disease; Poor compliance
Ganesapandian 2019	n= 100	52%	Adults	26 ± 22	South Indian	Epilepsy	Epilepsy diagnosis; CBZ monotherapy for ≥1 month to achieve steady state; Aged 18-65 years	Pregnant or lactating women; Receiving drugs that are substrates, inducers or inhibitors of CYP3A5; Alcoholics; Hepatic or renal dysfunction; Poor compliance
Lu 2018	n= 62	48%	Children, Adolescents and Adults	24 ± 16	Han Chinese	Epilepsy	Taking CBZ monotherapy for >3 months; Data for CBZ concentration and clinical data was available	Taking drug known to influence CYP450 and UGT2B7; Substance abuse history; Serious ADRs; Poor compliance; Progressive and degenerative neurological or systematic disease; Liver or kidney dysfunction
Zhang 2018	n= 78	45%	Children, Adolescents and Adults	36 ± 18	Chinese	Epilepsy	Outpatients or Inpatients; Taking CBZ for >1 month	Serious liver or kidney dysfunction; Significant side-effects; Taking other ASDs
Liu 2016	n= 67	NI	NI	NI	Chinese	NI	NI	NI

© 2024 Milosavljević F et al. *JAMA Network Open.*

Table S5. Continued

Trial	Sample	% of females	Age category	Average age	Ethnicity	Diagnosis	Inclusion criteria	Exclusion criteria
Chbili 2016	n= 118	56%	Adults	38 ± 17	Tunisian	Epilepsy	Epilepsy diagnosis; Taking CBZ monotherapy	Other brain disorder; Liver or renal disease; Taking other ASDs or food known to influence CBZ metabolism
Daci 2015	n= 145	43%	Adults	33 ± 16	Kosovar Albanian	Epilepsy	Epilepsy diagnosis; Aged 18-70 years; taking CBZ monotherapy ≥1 year; Demographic and clinical data was available	Taking drugs for other pathologies; Abnormal renal or hepatic functions; Dose change 1 month prior to sample collection
Wang 2015	n= 66	44%	Adults	33 ± 13	Chinese	Epilepsy	Epilepsy diagnosis; Taking CBZ monotherapy for >1 month on the same dose; Demographic and clinical data was available	Liver or renal disease; Taking drugs or traditional Chinese medicine known to influence CBZ metabolism; Poor compliance
Ma 2015	n= 166	45%	Adults	35 ± 16	Han Chinese	Epilepsy	Epilepsy diagnosis; Taking CBZ monotherapy; Good seizure control	CBZ allergy; Severe ADRs; Unreliable record of seizure frequency; Substance abuse; Hepatic or renal failure; Progressive and degenerative neurological or systematic disease
Liu 2014	n= 40	NI	NI	NI	Han Chinese	Epilepsy	Epilepsy diagnosis; Taking CBZ monotherapy	NI
Zhu 2014	n= 210	43%	Children, Adolescents and Adults	19 ± 15	Chinese	Epilepsy	Epilepsy diagnosis; Aged 2-86 years; Taking CBZ monotherapy for ≥6 months; Taking CBZ >1 year; Same CBZ dose for ≥1 month	Liver or renal disease
Saruwatari 2014	n= 104	47%	Children, Adolescents and Adults	12 ± 7	Japanese	Epilepsy	Taking immediate release CBZ for ≥4 week; Had detailed medical data	Taking other ASDs known to induce CYP3A activity; Taking other drugs known to influence disposition of CBZ; Abnormal Kidney or liver function

© 2024 Milosavljević F et al. *JAMA Network Open.*

Table S5. Continued

Trial	Sample	% of females	Age category	Average age	Ethnicity	Diagnosis	Inclusion criteria	Exclusion criteria
Panomvana 2013	n= 36	58%	Adults and Adolescents	43 ± 15	Thai	Epilepsy	Epilepsy diagnosis; >13 years old; Outpatients; Taking CBZ monotherapy; stable drug regime for 6 weeks; TDM data was available	Pregnant women; Hepatic or renal disease; Co-medication known to interfere with CBZ pharmacokinetics; Consuming grapefruit
Yun 2013	n= 83	36%	Adults	36 ± 17	Chinese	Epilepsy	Epilepsy diagnosis; Taking CBZ for ≥1 year	Liver or renal disorder
Hung 2012	n= 234	47%	Adults	39 ± 12	Han Chinese	Epilepsy	Epilepsy diagnosis; Same dose for ≥1 year; Good compliance and seizure control	Taking other ASDs; Taking drugs that may interact with CBZ
Meng 2011	n= 84	37%	Adults and Adolescents	36 ± 16	Chinese	Epilepsy	Epilepsy diagnosis; Taking CBZ for >1 week;	Taking other drugs that could affect liver enzyme activities; Serious liver or kidney disease; Poor compliance;
Park 2009	n= 35	23%	Adults	35 ± 13	Korean	Epilepsy	Taking CBZ for ≥3 month; Aged 20 - 60 years; At least one CBZ steady state level was known; Demographic and clinical data was known	Taking drugs known to influence CYP450 activity; Liver or renal disease; Poor compliance defined as >25% deviation in CBZ levels on the same dose regimen
Seo 2006	n= 144	45%	Children, Adolescents and Adults	16 ± 8	Japanese	Epilepsy	Epilepsy diagnosis; Taking CBZ for ≥2 weeks	Abnormal liver and kidney function

© 2024 Milosavljević F et al. *JAMA Network Open.*

Table S6. Study design of included trials on Phenytoin: Table summarizes the design, intervention data and genetic data for clinical trials included in any phenytoin related meta-analysis performed. If only a subset of the cohort could be included in meta-analysis, only characteristic of that specific subset are presented. Doses are presented in Mean ± Sd and/or (Range). **: Estimated; NI: No information; ASD: Anti-seizure drug; P: Prospective; R: Retrospective.*

**Trial**	**Study design**	**Dosing regimen**	**Dose**	**Time of blood sampling**	**Co-medications (% of cohort taking the given drug/class)**	**CYP2C9 Alleles searched**	**CYP2C19 Alleles searched**
Shaul 2022	P	Single dose	300 mg (Same dose)	12h after the last dose	No co-medications	*2, *3	None
Wanounou 2022	P	Single dose	300 mg (Same dose)	24h after single dose	No co-medications	*2, *3, *5, *6, *8, *11	None
Fohner 2019	R	Steady state*	300 mg/day (Same dose)	NI	NI	*2, *3, *5, *8, *11, *12	None
Guevara 2017	P	Steady state	(1.9 - 8.9) mg/kg/day	Before the next scheduled morning dose	NI	*2, *3	*2, *3
Li Z 2016	P	Steady state	(4.0 – 7.0) mg/kg/day	Either 2h before of 3h after the morning next dose	56% Cimetidine; 31% Metronidazole; 24% Phenobarbital etc.	*2, *3	*2, *3 (not included)
Ortega-Vázquez 2015	P	Steady state	(100 - 600) mg/day	Before the next scheduled morning dose	29% CYP2C9 inducers; 14% CYP2C9 inhibitors; 6% No CYP2C interfering drugs; 30% other ASDs	*2, *3, IVS8-109 A>T	*2, *3, *17
Sharma 2015	R	Steady state	5.00 ± 0.88 mg/kg	Before the next scheduled morning dose	No other ASDs; NI for other drugs	*2, *3	None
Yamamoto 2015	P	Steady state	7.1 ± 2.1 mg/kg/day	Between 2-5h after the scheduled dose	40% Valproate; 31% Carbamazepine; 29% Zonisamide;10% Phenobarbital; 14% Clobazam; etc.	*3	*2, *3
George 2012	P	Single dose	300 mg (Same dose)	3h after the single dose	100% Antituberculotic drugs; No other CYP2C9 inducers or inhibitors; NI for other drugs	NI	None
Hung 2012	P	Steady state	315 ± 86 mg/day	NI	No Phenytoin interacting drug; No other ASDs; NI for other drugs	*3	*2, *3

© 2024 Milosavljević F et al. *JAMA Network Open.*

Table S6. Continued

Trial	Study design	Dosing regimen	Dose	Time of blood sampling	Co-medications (% of cohort taking the given drug/class)	CYP2C9 Alleles searched	CYP2C19 Alleles searched
Kesavan 2010	P	Steady state	5.16 ± 1.36 mg/kg/day	Between 4-14h after scheduled dose	11% Phenobarbitone	*2, *3	*2, *3
Lin 2008	P	Steady state	302 ± 91 mg/day	Between 5-15h after scheduled dose	NI	*2, *3	*2, *3 (not included)
Lee 2007	P	Steady state	5.42 ± 1.3 mg/kg/day	NI	31% Carbamazepine; 20% Valproate; 13% Phenobarbital etc.	*3	*2, *3
Rosemary 2006	P	Single dose	300 mg (Same dose)	4h after the single dose	NI	*2, *3	*2, *3 (not included)
Yamanaka 2005	P	Steady state	217 ± 39 mg/kg	NI	27% Phenobarbital or Valproate or betamethasone or amiodarone	None	*2, *3
Taguchi 2005	P	Steady state	5.2 ± NI mg/kg/day	NI	80% other ASDs; NI for other drugs	None	*2, *3
Huang 2004	P	Steady state	(1 – 18) mg/kg/day	Before the next dose	No other ASDs; NI for other drugs	None	*2, *3
Hung 2004	P + R	Steady state	294 ± 75 mg/day	Variable	55% other ASDs; 37% CYP2C9 inducers (phenobarbital, primidone, carbamazepine); 18% CYP2C9 inhibitors (Valproate)	*2, *3	*2, *3
Soga 2004	P	Steady state	4.17 ± 2.01 mg/kg/day	At least 2h after the scheduled dose	NI	*2, *3	*2, *3
Caraco 2001	P	Single dose	300 mg (Same dose)	Multiple times during 96h after the single dose	No co-medications	*2, *3	*2, *3 (Not included)
van der Weide 2001	P	Steady state	(75 – 425) mg/day	2h before the next dose	27% Phenobarbital; 18% Carbamazepine; 7% Valproate etc.	*2, *3	*2 (Not included)
Aynacioglu 1999	P	Single dose	300 mg (Same dose)	12h after the scheduled dose	No co-medications	*2, *3	None
Mamiya 1998	P	Steady state	199 ± 79 mg/day	NI	88% other ASDs	*2, *3	*2, *3

© 2024 Milosavljević F et al. *JAMA Network Open.*

Table S7. Study design of included trials on Valproic acid: Table summarizes the design, intervention data and genetic data for clinical trials included in any valproic acid related meta-analysis performed. If only a subset of the cohort could be included in meta-analysis, only characteristic of that specific subset are presented. Doses are presented in Mean ± Sd and/or (Range). [a] *Genotyping for this gene was performed, but the data could not be included; NI: No information; ASD: Anti-seizure drug; P: Prospective; R: Retrospective; VPA: Valproic acid.*

**Trial**	**Study design**	**Dosing regimen**	**Dose**	**Time of blood sampling**	**Co-medications (% of cohort taking the given drug/class)**	**CYP2C19 Alleles searched**	**CYP2C9 Alleles searched**	**UGT1A6 Alleles searched**	**UGT2B7 Alleles searched**
Zheng 2023	P	Steady state	(600 – 1200) mg/day	12h after the last evening dose and before 9 AM the next morning	NI	*2, *3	N/A	19T>G 541A>G 552A>C	802C>T -161C>T 211G>T
Wang 2021	P	Steady State	(500 – 1500) mg/day	At 8am	Olanzapine	*2, *3	/	/	/
Du 2021	P	Steady State	23.7 ± 7.0 mg/kg/day	1h before a scheduled dose	No drugs that interfere with VPA; NI for other drugs	*2, *3	*3	19T>G 541A>G 552A>C	802C>T -161C>T 211G>T
Wu 2021	R	Steady State	22.6 ± 5.66 mg/kg/day	Before a scheduled morning dose	47% no other ASDs; 23% one additional ASD; 18% two additional ASDs; 12% three or more additional ASDs	N/A	*3	N/A	N/A
Xu 2021	R	Steady State	20 mg/kg/day (oral solution); 10 - 15 mg/kg/day (tablets)	30 min before the scheduled dose for some patients, variable for the rest of the cohort	No other ASDs; NI for other drugs	N/A	*3	19T>G 541A>G	802C>T -161C>T 211G>T
Nandith 2021	P	Steady State	20 mg/kg twice or thrice a day	NI	No other ASDs; No enzyme inducers or inhibitors; NI for other drugs	N/A	N/A	19T>G 541A>G 552A>C	-161C>T 211G>T
Song 2020	P	Steady State	872 ± 356 mg/day	1 hour before a next scheduled morning dose	No other ASDs; NI for other drugs	*2, *3	*13	N/A	N/A
Zhao 2020	P	Steady State	19.9 ± 6.2 mg/kg/day	Before a scheduled morning dose	No other ASDs; NI for other drugs	N/A	N/A	19T>G 541A>G 552A>C	802C>T -161C>T

© 2024 Milosavljević F et al. *JAMA Network Open.*

Table S7. Continued

Trial	Study design	Dosing regimen	Dose	Time of blood sampling	Co-medications (% of cohort taking the given drug/class)	CYP2C19 Alleles searched	CYP2C9 Alleles searched	UGT1A6 Alleles searched	UGT2B7 Alleles searched
Wang 2019	P	Steady State	500 ± 180 mg/day	NI	15% Levetiracetam;<br>6% Topiramate;<br>4% Oxcarbazepine etc.	N/A	N/A	541A>G	-161C>T
Feng 2019	P	Steady State	655 ± 296 mg/day	2 h before a scheduled morning dose	21% Lamotrigine;<br>21% Carbamazepine,<br>21% Oxcarbamazepine;<br>14% Levetiracetam	N/A	*3	19T>G;<br>552A>C	802C>T<br>-161C>T<br>211G>T
Feng 2018	P	Steady State	636 ± 278 mg/day	2 h before a scheduled morning dose	5% Lamotrigine;<br>3% Levetiracetam;<br>1% Carbamazepine etc.	*2, *3	*3	19T>G	802C>T<br>-161C>T<br>211G>T
Zhang 2017	P	Steady State	434 ± 192 mg/day	In the morning	No other ASDs;<br>NI for other drugs	N/A	N/A	N/A	802C>T
Sun 2017	P	Steady State	Starting continuous IV infusion:<br>1600 mg/day<br>Maximum:<br>3000 mg/day	In the morning	Midazolam (IV) or Levetiracetam if needed;<br>No enzyme inductors or inhibitors;<br>NI for other drugs	*2, *3	*2, *3	N/A[a]	802C>T<br>211G>T
Mei 2017	P	Steady State	19.9 ± 5.9 mg/kg/day	2 h before a scheduled morning dose	No other ASDs;<br>NI for other drugs	*2, *3	*3	19T>G	802C>T<br>-161C>T<br>211G>T
Li Z 2017	P	Steady State	Intravenous:<br>1200 mg/day	After the surgery	7% Carbapenems, Glycerol fructose, Musk and/or Mannitol	N/A	*3	541A>G<br>552A>C	802C>T
Wang 2017	P	Steady State	20.1 ± 6.4	Before a scheduled morning dose, ~12h after the evening dose	No other ASDs;<br>NI for other drugs	N/A	*3	N/A	N/A
Zhao 2017	P	Steady State	19.9 ± 6.5	Before a scheduled morning dose	No other ASDs;<br>NI for other drugs	N/A	*3	N/A	N/A

© 2024 Milosavljević F et al. *JAMA Network Open.*

Table S7. Continued

Trial	Study design	Dosing regimen	Dose	Time of blood sampling	Co-medications (% of cohort taking the given drug/class)	CYP2C19 Alleles searched	CYP2C9 Alleles searched	UGT1A6 Alleles searched	UGT2B7 Alleles searched
Jogamoto 2017	R	Steady State	28.1 ± 10.1 mg/kg/day	Variable	100% Stiripentol;<br>68% Clobazam;<br>29% Topiramat;<br>19% K-Bromide etc.	*2, *3	N/A	N/A	N/A
Algharably 2016	P	Steady State	20.6 ± 7.1 mg/kg/day	Before a scheduled morning dose	No other ASDs:<br>No enzyme inductors or inhibitors;<br>NI for other drugs	N/A	N/A	541A>G<br>552A>C	N/A
Kang 2016	P	Steady State	(80 – 2000) mg/day	Before a scheduled morning dose	No other ASDs;<br>NI for other drugs	N/A	N/A	541A>G<br>552A>C	N/A
Feng 2016	P	Steady State	444 ± 189 mg/day	NI	4% Lamotrigine;<br>4% Levetiracetam;<br>4% Clonazepam etc.	*2, *3	N/A[a]	19T>G	802C>T
Du 2016	P	Steady State	17.6 ± 6.4 mg/kg/day	Before a scheduled morning dose	NI	N/A	N/A	N/A	802C>T<br>211G>T
Chatzistefanidis 2016	P	Steady State	Children:<br>24.7 ± 13<br>Adults:<br>18.9 ± 8.8<br>mg/kg/day	Immediately before a scheduled morning dose	No enzyme inducers/inhibitors;<br>No drugs interacting with protein binding;<br>37% Levetiracetam, Lacosamide, Pregabalin, Gabapentin or Zonisamide	N/A	N/A	19T>G<br>541A>G<br>552A>C	802C>T
Shen 2016	P	Steady State	16.3 ± 4.6 mg/kg/day	NI	No other Antiseizure drugs;<br>NI for other drugs	N/A	N/A	541A>G;<br>552A>C	211G>T
Wang 2016	P	Steady State	17.3 ± 8.7 mg/kg/day	In the early morning	100% Lamotrigine;<br>No drugs known to influence pharmacokinetics of VPA;<br>NI for other drugs	N/A	N/A	N/A	802C>T<br>-161C>T<br>211G>T
Wen 2016	R	Steady State	19.9 ± 9.76 mg/kg/day	Before a scheduled morning dose	6% Meropenem;<br>No enzyme inducers or inhibitor;<br>NI for other drugs	N/A	N/A	N/A	-161C>T

© 2024 Milosavljević F et al. *JAMA Network Open.*

Table S7. Continued

Trial	Study design	Dosing regimen	Dose	Time of blood sampling	Co-medications (% of cohort taking the given drug/class)	CYP2C19 Alleles searched	CYP2C9 Alleles searched	UGT1A6 Alleles searched	UGT2B7 Alleles searched
Smith 2016	R	Steady State	(84 – 5400) mg/day	10–16 h after the last dose (in 10% of samples may vary between 10–26 h)	No carbamazepine, phenobarbital or phenytoin; NI for other drugs	*2, *3, *4, *17	*2, *3	N/A	N/A
Sun 2015	P	Steady State	(500 – 1000) mg/day	Immediately before a scheduled morning dose	NI	N/A	N/A	N/A	802C>T 211G>T
Han 2015	NI	NI	NI	NI	NI	*2, *3	N/A	N/A	N/A
Tóth 2015	P	Steady State	(30 – 40) mg/kg/day (Target)	Before a scheduled morning dose	No other Antiseizure drugs; NI for other drugs	N/A	*2, *3	N/A	N/A
Jain 2015	P	Steady State	21.8 ± 9.1 mg/kg/day	Immediately before a scheduled morning dose	No drugs interfering with VPA metabolism; NI for other drugs	N/A	N/A	19T>G 541A>G 552A>C	N/A
Aphichartphun kawee 2014	P	Steady State	20.7 ± 10.8 mg/day/kg	Before a scheduled morning dose	44% Enzyme inducers; 56% drugs with no effect on the enzymes; 20% Clonazepam etc.	N/A	N/A	19T>G 541A>G 552A>C	N/A
Inoue 2014	P	Steady State	24.4 ± 9.1 mg/kg/day	During the peak steady-state period, 2.5–3 h after a meal	47% No other ASDs; 22% Clobazam; 14%, Zonisamide; 6% Levetiracetam etc.	N/A	N/A	N/A	-161C>T
Liao 2013	P	Steady State	22.9 ± 6.4 mg/kg/day	Before a scheduled morning dose	No other ASDs; NI for other drugs	*2, *3	*2, *3	N/A	N/A
Ma 2013	P	Steady State	(10 – 40) mg/kg/day	Before a scheduled morning dose	No co-medications	N/A	N/A	N/A	211G>T
Rutigliano 2013	NI	NI	11.3 ± 4.8 mg/kg/day	12h after the last dose	NI	*2	N/A	N/A	N/A
Jin 2013	P	Steady State	(10 – 30) mg/kg/day	Early in the morning before the next dose	NI	N/A	N/A	541A>G	N/A

© 2024 Milosavljević F et al. *JAMA Network Open.*

Table S7. Continued

Trial	Study design	Dosing regimen	Dose	Time of blood sampling	Co-medications (% of cohort taking the given drug/class)	CYP2C19 Alleles searched	CYP2C9 Alleles searched	UGT1A6 Alleles searched	UGT2B7 Alleles searched
Guo 2012	P	Steady State	17.2 ± 15.5 mg/kg/day	Before a scheduled dose	No other drugs that modulate activities of UGT1A6, UGT2B7 and CYP2C9; NI for other drugs	N/A	*3	N/A[a]	802C>T
Chu 2012	P	Steady State	(250 – 1000) mg/day	Immediately before a scheduled morning dose	56% No other ASDs; 16% carbamazepine; 8% Topiramate; 3% Piracetam etc.	N/A	N/A	541A>G 552A>C	802C>T
Sterjev 2012	NI	NI	932 ± 354 mg/day	NI	NI	N/A	N/A	541A>G	N/A
Hung 2011	P	Steady State	20.0 ± 0.7 mg/kg/day	NI	No other antiseizure drugs; No drugs that may interact with VPA; NI for other drugs	N/A	N/A	19T>G 541A>G 552A>C	802C>T -161C>T
Wang 2010	P	Steady State	NI	Before a scheduled morning dose	NI	N/A	N/A	541A>G	N/A
Tan 2010	P	Steady State	NI	12 h after the evening dose	No other antiseizure drugs; NI for other drugs	N/A	*3	N/A	N/A
Chung 2008	P	Steady State	600 ± NI mg/day	0.25, 0.5, 1, 1.5, 2, 4, 6, 8, 12, 24, and 48 h after the last dose	100% Lorazepam, No other drugs	N/A	N/A	N/A	802C>T
Sun 2007	P	Steady State	(10 – 30) mg/kg/day	Early in the morning before a scheduled dose	NI	N/A	N/A	552A>C	N/A

© 2024 Milosavljević F et al. *JAMA Network Open.*

Table S8. Study design of included trials on Lamotrigine: Table summarizes the design, intervention data and genetic data for clinical trials included in any Lamotrigine related meta-analysis performed. If only a subset of the cohort could be included in meta-analysis, only characteristic of that specific subset are presented. Doses are presented in Mean ± Sd and/or (Range). Comedications known to greatly influence lamotrigine metabolism are presented in bold. *: Estimated; NI: No information; ASD: Anti-seizure drug; P: Prospective; R: Retrospective; LTG: Lamotrigine; VPA: Valproic acid; PHT: Phenytoin; CBZ: Carbamazepine; OXC: Oxcarbazepine.

Trial	Study design	Dosing regimen	Dose	Time of blood sampling	Co-medications (% of cohort taking the given drug/class)	UGT1A4 Alleles searched	UGT2B7 Alleles searched
Božina 2023	P	Steady state	(12.5 – 550) mg/day	Between the 07:00 – 09:00 h	**30% VPA (statistically adjusted);** No drugs interacting with VPA or LTG	142T>G (*3)	-161C>T
Petrenaite 2022	P	Steady state	332 ± 170 mg/day	Before the morning dose of LTG ≥10 h after the latest dose; Not standardized across all patients	53% monotherapy; 37% other ASDs; 10% Non-interacting ASDs and oral contraceptives	142T>G (*3)	802C>T
Ortega-Vázquez 2020	P	Steady state	237 ± 110 mg/day	Just before the next scheduled morning dose	20% Monotherapy; **28% inhibitors (VPA); 25% inducers (PHT, CBZ, or OXC),** 28% inhibitor + inducer	Multiple (142T>G included)	-161C>T, 802C>T
Suzuki 2019 Monotherapy	P	Steady state	100 mg/day (Same dose)	At 08:00	54% antidepressants; 62% mood stabilizers; 52% atypical antipsychotics	142T>G (*3)	-161C>T
Suzuki 2019 +VPA			75 mg/day (Same dose)		100% VPA; 54% antidepressants; 62% mood stabilizers; 52% atypical antipsychotics	142T>G (*3)	-161C>T
Petrenaite 2018	R	Steady state	(100 – 1550) mg/day	Before the morning dose of LTG ≥10 h after the latest dose; Not standardized across all patients	80% monotherapy; 10% non-ASDs (acetylsalicylic acid, levothyroxine, tinzaparin, tramadol); 5% Topiramate;	142T>G (*3)	802C>T
Smith 2018	R	NI	456 ± 193 mg/day	10–30 hours after the last dose intake	**9% VPA;** No other enzyme inducers (carbamazepine, phenytoin, and phenobarbital)	142T>G (*3)	None

© 2024 Milosavljević F et al. *JAMA Network Open.*

Table S8. Continued

Trial	Study design	Dosing regimen	Dose	Time of blood sampling	Co-medications (% of cohort taking the given drug/class)	UGT1A4 Alleles searched	UGT2B7 Alleles searched
He 2017	P	Steady state	1.83 ± 0.87 mg/kg/day	NI	100% VPA;<br>No drugs that may cause renal stones in the past 6 months	142T>G (*3)	None
Du 2016	P	Steady state	2.74 ± 1.17 mg/kg/day	Before the next scheduled morning dose	NI	142T>G (*3)	None
Reimers 2016	P	Steady state	(50 – 800) mg/day	10–24 h after the last dose	**25% VPA;**<br>35% non-interacting ASDs;<br>11% other drugs	142T>G (*3)	None
Wang 2016	P	Steady state	NI	On an empty stomach in the early morning	100% VPA;<br>No drugs known to influence the pharmacokinetics of VPA and/or LTG;<br>NI for other drugs	None	-161C>T, 802C>T
Liu 2015 Monotherapy	P	Steady state	2.31 ± 1.39 mg/kg/day	Before the next scheduled morning dose	100% monotherapy;<br>NI for other drugs	142T>G (*3)	-161C>T, 802C>T
Liu 2015 +VPA			2.78 ± 1.65 mg/kg		Valproate 100%,<br>NI for other drugs		
Wang 2015	P	Steady state	2.83 ± 1.77 mg/kg/day	On empty stomach in the early morning	100% VPA;<br>NI for other drugs	142T>G (*3)	None
Zhou 2015	P	Steady state	146 ± 75 mg/day	Before the next scheduled morning dose	No other ASDs;<br>NI for other drugs	None	-161C>T
Chang 2014	P	Steady state	(0.41 - 4.30) mg/kg/day	Before the next scheduled morning dose	No other ASDs;<br>No drugs that influence the liver enzyme activity	142T>G (*3)	None

© 2024 Milosavljević F et al. *JAMA Network Open.*

Table S9. Study design of included trials on Carbamazepine: Table summarizes the design, intervention data and genetic data for clinical trials included in any Carbamazepine related meta-analysis performed. If only a subset of the cohort could be included in meta-analysis, only characteristic of that specific subset are presented. Doses are presented in Mean ± Sd and/or (Range). *NI: No information; ASD: Anti-seizure drug; P: Prospective; R: Retrospective; CBZ: Carbamazepine.*

Trial	Study design	Dosing regimen	Dose	Time of blood sampling	Co-medications (% of cohort taking the given drug/class)	CYP3A5	EPHX1	UGT2B7	Active metabolite data was reported
Venkatraman 2023	P	Steady state	(6.2 – 12) mg/kg/day	NI	No other ASDs;<br>NI for other drugs	/	337C>T	802C>T	Yes
Pham 2020	P	Steady state	7.7 ± 3.7 mg/kg/day	30 minutes before the next scheduled morning dose	No other ASDs;<br>No drugs known to affect liver enzyme activity	*3	/	/	No
Ganesapandian 2019	P	Steady state	600 ± 602 mg/day	NI	No other ASDs;<br>No inducers or inhibitors or substrates of CYP3A5<br>NI for other drugs	*3	/	/	No
Lu 2018	P	Steady state	499 ± 278 mg/day	Before the next scheduled morning dose	No other ASDs;<br>No drugs that influence CYP450 or UGT2B7;<br>NI for other drugs	*3	/	802C>T	No
Zhang 2018	P	Steady state	4.81 ± 1.99 mg/kg/day	In the morning	No other ASDs;<br>NI for other drugs	/	/	802C>T	No
Liu 2016	P	NI	NI	NI	NI	*3	/	/	No
Chbili 2016	P	Steady state	8.51 ± 3.21 mg/kg/day	NI	No other ASDs;<br>NI for other drugs	/	337C>T<br>416A>G	/	Yes
Daci 2015	P	Steady state	8.18 ± 3.91 mg/kg/day	Early in the morning	No other ASDs;<br>No drugs for other pathologies	/	337C>T<br>416A>G	/	Yes
Wang 2015	P	Steady state	428 ± 204 mg/day	Between 07:00 - 09:00;<br>Before the next scheduled morning dose	No other ASDs;<br>No drugs known to influence CBZ metabolism	*3	/	/	Yes

© 2024 Milosavljević F et al. *JAMA Network Open.*

Table S9. Continued

Trial	Study design	Dosing regimen	Dose	Time of blood sampling	Co-medications (% of cohort taking the given drug/class)	CYP3A5	EPHX1	UGT2B7	Active metabolite data was reported
Ma 2015	P	Steady state	10.04 ± 3.14 mg/kg/day	Before the next scheduled morning dose	No other ASDs;<br>NI for other drugs	*3	337C>T	802C>T	Yes
Liu 2014	P	NI	NI	NI	No other ASDs;<br>NI for other drugs	*3	/	/	No
Zhu 2014	P	Steady state	10.9 ± 5.0 mg/kg/day	Before the next scheduled dose	No other ASDs;<br>NI for other drugs	*3	337C>T<br>416A>G	/	Yes
Saruwatari 2014	R	Steady state	245 ± 162 mg/day	1-4h after CBZ dose was taken	No ASDs known to induce CYP3A activity; No drugs known to influence disposition of CBZ	*3	/	/	No
Panomvana 2013	R	Steady state	14.1 ± 5.5 mg/kg/day	Before the next scheduled morning dose	No relevant co-medications	*3	/	/	No
Yun 2013	P	Steady state	7.3 ± 2.7 mg/kg/day	In the early morning, before breakfast	No other ASDs;<br>NI for other drugs	/	337C>T<br>416A>G	/	No
Hung 2012	P	Steady state	849 ± 300 mg/day	NI	No other ASDs;<br>No drugs that may interact with CBZ	/	337C>T<br>416A>G	-<br>161C>T	No
Meng 2011	P	Steady state	7.0 ± 2.5 mg/kg/day	Early morning on empty stomach; Before the next scheduled morning dose	No other ASDs;<br>No drugs known to influence Liver enzyme activities	*3	/	/	No
Park 2009	P	Steady state	11.9 ± 6.5 mg/kg/day	Before 07:00 - 09:00;<br>Before the next scheduled morning dose	No drugs known to influence CYP450 activity;<br>NI for other drugs	*3	/	/	No
Seo 2006	P	Steady state	11.1 ± 5.5 mg/kg/day	Varies	35% VPA;<br>11% Phenytoin<br>19% Phenobarbital	*3	/	/	No

© 2024 Milosavljević F et al. *JAMA Network Open.*

3) Forest plots – RoM (Ratio of means)

3.1) Phenytoin related RoM meta-analyses (Figures S5-S8)

Study or Subgroup	log[Ratio of Means]	SE	IMs Total	NMs Total	Weight	Ratio of Means IV, Random, 95% CI
Ortega-Vázquez 2015	-0.06899287	0.28262446	3	61	2.1%	0.93 [0.54, 1.62]
Rosemary 2006	0.04514385	0.25778102	16	10	2.4%	1.05 [0.63, 1.73]
Mamiya 1998	0.0704314	0.21472797	3	131	3.0%	1.07 [0.70, 1.63]
Lin 2008	0.34898677	0.18409459	10	43	3.5%	1.42 [0.99, 2.03]
Guevara 2017	0.26254969	0.15575956	16	34	4.1%	1.30 [0.96, 1.76]
Li Z 2016	0.39189074	0.14213563	6	56	4.5%	1.48 [1.12, 1.96]
Soga 2004	0.56349585	0.13305942	3	25	4.7%	1.76 [1.35, 2.28]
Sharma 2015	0.63335263	0.1301682	9	26	4.8%	1.88 [1.46, 2.43]
van der Weide 2001	0.27548189	0.12354419	21	37	4.9%	1.32 [1.03, 1.68]
Caraco 2001	0.46242733	0.11713357	12	18	5.1%	1.59 [1.26, 2.00]
Hung 2012	0.48404487	0.10807798	16	252	5.3%	1.62 [1.31, 2.01]
Lee 2007	0.58238257	0.1029236	9	87	5.5%	1.79 [1.46, 2.19]
Hung 2004	0.37627832	0.10135071	18	151	5.5%	1.46 [1.19, 1.78]
George 2012	-0.07437697	0.07383354	11	36	6.2%	0.93 [0.80, 1.07]
Yamamoto 2015	0.33382638	0.06987885	13	157	6.3%	1.40 [1.22, 1.60]
Fohner 2019	0.44222929	0.069514	88	231	6.3%	1.56 [1.36, 1.78]
Aynacioglu 1999	0.31220611	0.06910889	32	68	6.3%	1.37 [1.19, 1.56]
Wanounou 2022	0.41866266	0.06830527	16	127	6.3%	1.52 [1.33, 1.74]
Kesavan 2010	0.74616759	0.06692982	40	244	6.4%	2.11 [1.85, 2.40]
Shaul 2022	0.40836306	0.04982511	67	69	6.7%	1.50 [1.36, 1.66]
**Total (95% CI)**			**409**	**1863**	**100.0%**	**1.46 [1.33, 1.61]**

Heterogeneity: Tau² = 0.03; Chi² = 88.66, df = 19 (P < 0.00001); I² = 79%
Test for overall effect: Z = 7.84 (P < 0.00001)

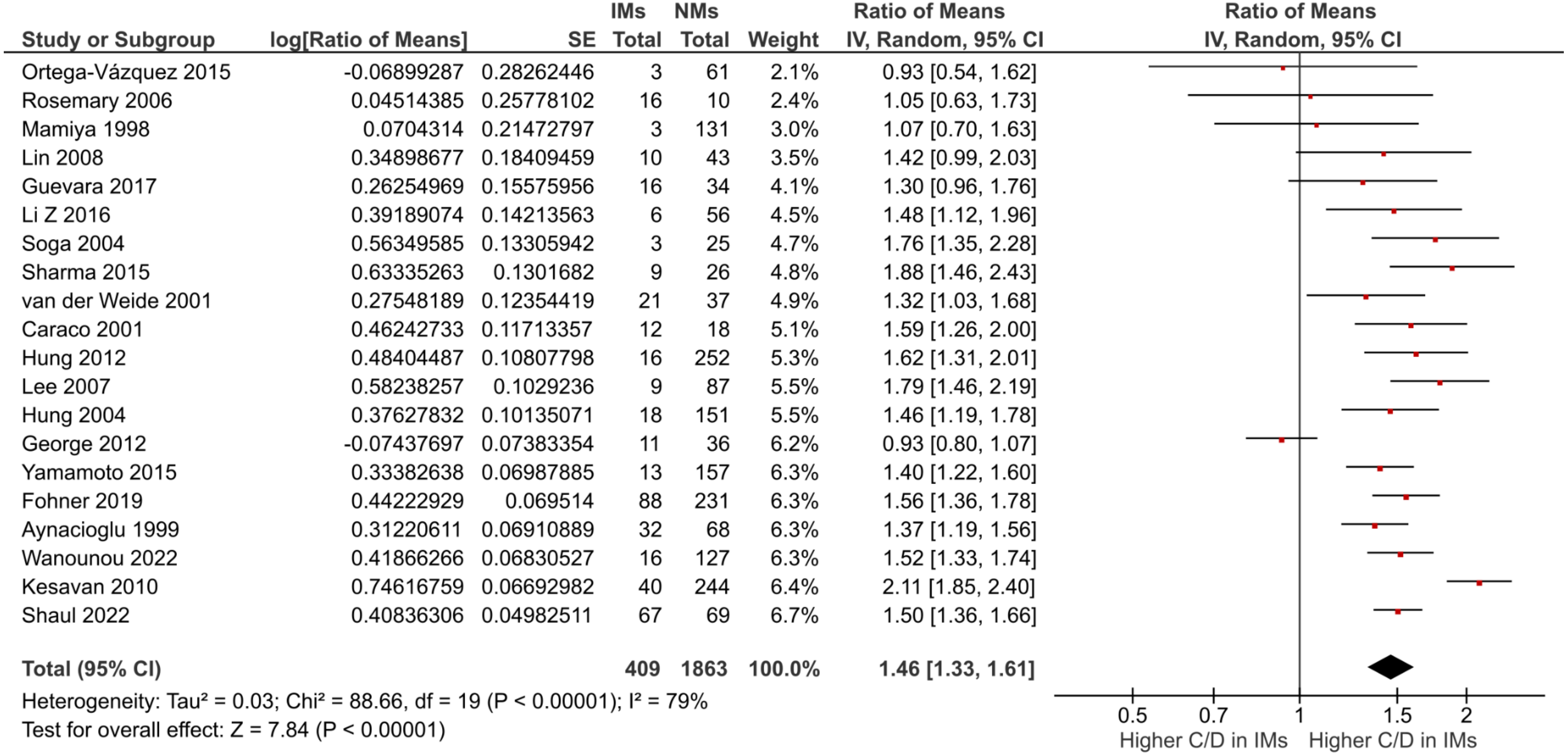

Figure S5. Forest plot (RoM): Phenytoin C/D in CYP2C9 IMs compared to CYP2C9 NMs. Based on the data from k=20 trials and N=2272 patients, CYP2C9 IMs had on average 46% (95%CI: 33%, 61%; df=19; p<0.001) higher C/D than CYP2C9 NMs. Considerable heterogeneity was observed (I^2=79%). *C/D: Plasma concentration-to-dose ratio; IMs: Intermediate metabolizers; NMs: Normal metabolizers (reference group).*

© 2024 Milosavljević F et al. *JAMA Network Open.*

Study or Subgroup	log[Ratio of Means]	SE	IMs Total	NMs Total	Weight	Ratio of Means IV, Random, 95% CI
Yamanaka 2005	0.31586218	0.35285464	3	10	0.5%	1.37 [0.69, 2.74]
Ortega-Vázquez 2015	-0.10536052	0.27361912	7	52	0.9%	0.90 [0.53, 1.54]
Taguchi 2005	0.37736789	0.2495744	9	5	1.0%	1.46 [0.89, 2.38]
Soga 2004	0.05298931	0.2493236	13	3	1.0%	1.05 [0.65, 1.72]
Kesavan 2010	0.09558871	0.24700774	130	103	1.1%	1.10 [0.68, 1.79]
Guevara 2017	0.07869434	0.1769984	10	40	2.1%	1.08 [0.76, 1.53]
Huang 2004	0.14310084	0.12261871	14	11	4.3%	1.15 [0.91, 1.47]
Lee 2007	0.35114243	0.09536418	44	36	7.1%	1.42 [1.18, 1.71]
Mamiya 1998	0.30228087	0.09226064	64	52	7.6%	1.35 [1.13, 1.62]
Hung 2004	0.10536052	0.07314156	88	47	12.1%	1.11 [0.96, 1.28]
Hung 2012	0.27338065	0.05244898	150	102	23.4%	1.31 [1.19, 1.46]
Yamamoto 2015	0.1804646	0.04065584	75	47	39.0%	1.20 [1.11, 1.30]
**Total (95% CI)**			**607**	**508**	**100.0%**	**1.23 [1.17, 1.30]**

Heterogeneity: Tau² = 0.00; Chi² = 10.55, df = 11 (P = 0.48); I² = 0%
Test for overall effect: Z = 8.23 (P < 0.00001)

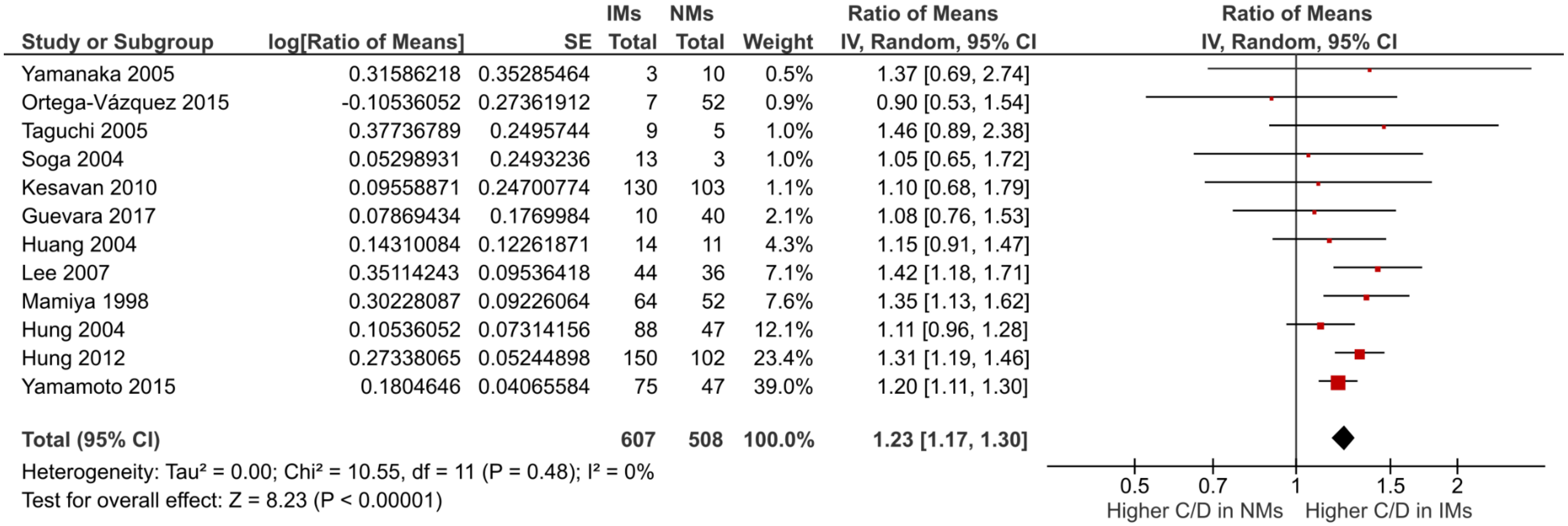

Figure S6. Forest plot (RoM): Phenytoin C/D in CYP2C19 IMs compared to CYP2C19 NMs. Based on the data from k=12 trials and N=1115 patients, CYP2C19 IMs had on average 23% (95%CI: 17%, 30%; df=11; p<0.001) higher C/D than CYP2C19 NMs. No heterogeneity was observed (I^2=0%). *C/D: Plasma concentration-to-dose ratio; IMs: Intermediate metabolizers; NMs: Normal metabolizers (reference group).*

© 2024 Milosavljević F et al. *JAMA Network Open.*

Study or Subgroup	log[Ratio of Means]	SE	PMs Total	NMs Total	Weight	Ratio of Means IV, Random, 95% CI
Taguchi 2005	0.12953842	0.31013521	6	5	3.2%	1.14 [0.62, 2.09]
Soga 2004	-0.0487652	0.28684603	9	3	3.7%	0.95 [0.54, 1.67]
Kesavan 2010	0.25288859	0.26702507	59	103	4.1%	1.29 [0.76, 2.17]
Lee 2007	0.70002006	0.14666604	7	36	10.6%	2.01 [1.51, 2.68]
Mamiya 1998	0.25782911	0.12416015	15	52	13.0%	1.29 [1.01, 1.65]
Hung 2004	0.39304259	0.09087392	16	47	18.0%	1.48 [1.24, 1.77]
Hung 2012	0.19767843	0.07297789	15	102	21.3%	1.22 [1.06, 1.41]
Yamamoto 2015	0.37320782	0.04899758	35	47	26.1%	1.45 [1.32, 1.60]
**Total (95% CI)**			**162**	**395**	**100.0%**	**1.39 [1.24, 1.56]**

Heterogeneity: Tau² = 0.01; Chi² = 13.46, df = 7 (P = 0.06); I² = 48%
Test for overall effect: Z = 5.68 (P < 0.00001)

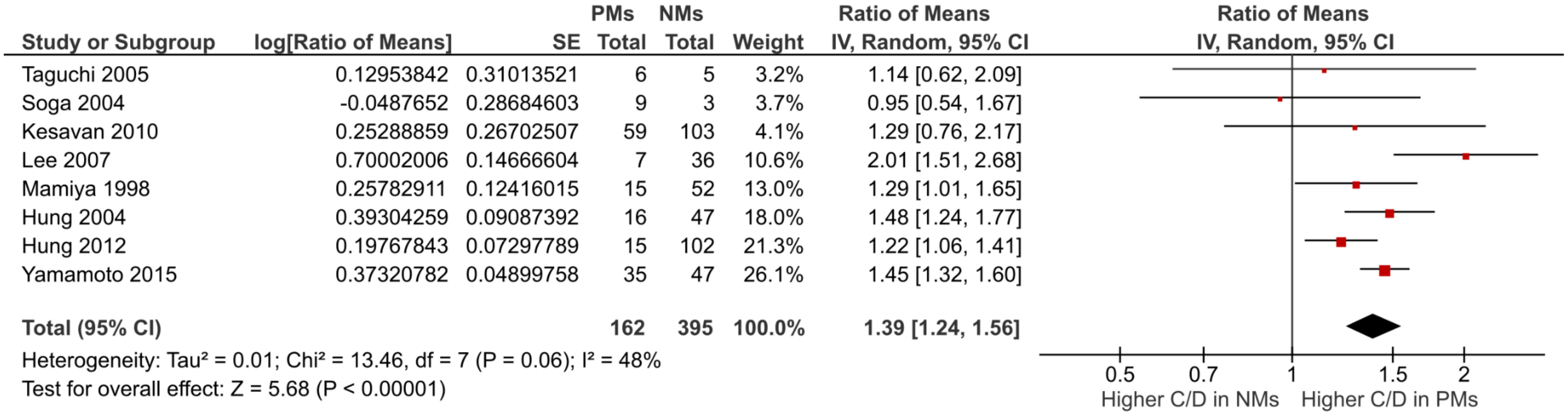

Figure S7. Forest plot (RoM): Phenytoin C/D in CYP2C19 PMs compared to CYP2C19 NMs. Based on the data from k=8 trials and N=557 patients, CYP2C19 PMs had on average 39% (95%CI: 24%, 56%; df=7; p<0.001) higher C/D than CYP2C19 NMs. Low level of heterogeneity was observed (I^2=48%). *C/D: Plasma concentration-to-dose ratio; PMs: Poor metabolizers; NMs: Normal metabolizers (reference group).*

© 2024 Milosavljević F et al. *JAMA Network Open.*

Study or Subgroup	log[Ratio of Means]	SE	IM+PMs Total	NMs Total	Weight	Ratio of Means IV, Random, 95% CI
Soga 2004	0.01260573	0.24228813	22	3	1.1%	1.01 [0.63, 1.63]
Taguchi 2005	0.28546882	0.22401931	15	5	1.2%	1.33 [0.86, 2.06]
Kesavan 2010	0.14739998	0.18935277	189	103	1.7%	1.16 [0.80, 1.68]
Lee 2007	0.40686147	0.09219948	51	36	7.3%	1.50 [1.25, 1.80]
Mamiya 1998	0.29399124	0.08628913	79	52	8.3%	1.34 [1.13, 1.59]
Hung 2004	0.15537094	0.07074393	104	47	12.4%	1.17 [1.02, 1.34]
Hung 2012	0.26673061	0.05055429	165	102	24.2%	1.31 [1.18, 1.44]
Yamamoto 2015	0.24591179	0.03759359	110	47	43.8%	1.28 [1.19, 1.38]
**Total (95% CI)**			**735**	**395**	**100.0%**	**1.29 [1.23, 1.35]**

Heterogeneity: Tau² = 0.00; Chi² = 6.34, df = 7 (P = 0.50); I² = 0%
Test for overall effect: Z = 10.12 (P < 0.00001)

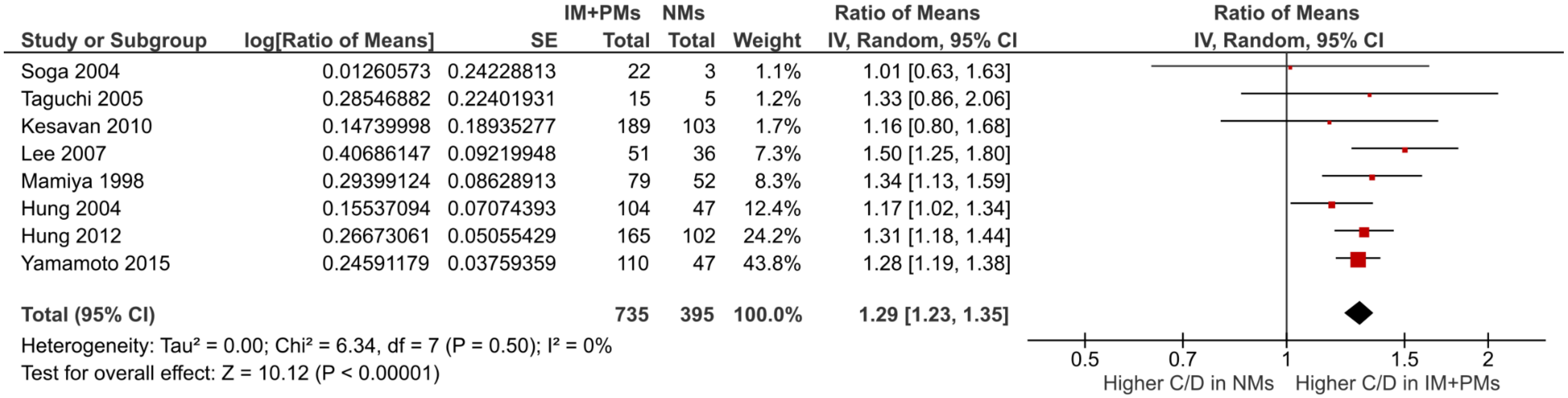

Figure S8. Forest plot (RoM): Phenytoin C/D in combined CYP2C19 IMs and PMs compared to CYP2C19 NMs. Based on the data from k=8 trials and N=1130 patients, carriers of either CYP2C19 IM or PM status had on average 29% (95%CI: 23%, 35%; df=7; p<0.001) higher C/D than CYP2C19 NMs. No heterogeneity was observed (I^2=0%). *C/D: Plasma concentration-to-dose ratio; PMs: Poor metabolizers; IMs: Intermediate metabolizers; NMs: Normal metabolizers (reference group).*

© 2024 Milosavljević F et al. *JAMA Network Open.*

3.2) Valproic acid related RoM meta-analyses (Figures S9-S17)

Study or Subgroup	log[Ratio of Means]	SE	IM Total	NM Total	Weight	Ratio of Means IV, Random, 95% CI
Mei 2017	-0.07644701	0.27910621	7	43	1.5%	0.93 [0.54, 1.60]
Wang 2017	-0.0325526	0.20725178	7	95	2.6%	0.97 [0.64, 1.45]
Xu 2021	-0.02928281	0.16518289	11	181	3.7%	0.97 [0.70, 1.34]
Tóth 2015	0.18785069	0.16016971	15	35	3.8%	1.21 [0.88, 1.65]
Feng 2018	-0.17261232	0.14514101	18	156	4.4%	0.84 [0.63, 1.12]
Li Z 2017	0.33031678	0.12370174	5	79	5.5%	1.39 [1.09, 1.77]
Wu 2021	0.11357801	0.11434085	15	91	6.0%	1.12 [0.90, 1.40]
Rutigliano 2013	0.04271773	0.10883599	7	62	6.4%	1.04 [0.84, 1.29]
Song 2020	-0.04255961	0.1054613	8	75	6.6%	0.96 [0.78, 1.18]
Liao 2013	0.43702231	0.09461469	16	115	7.4%	1.55 [1.29, 1.86]
Zhao 2017	0.22314355	0.0886991	11	189	7.9%	1.25 [1.05, 1.49]
Du 2021	0.19829755	0.0850986	21	208	8.2%	1.22 [1.03, 1.44]
Smith 2016	-0.00493733	0.07469971	67	182	9.2%	1.00 [0.86, 1.15]
Sun 2017	0.02686364	0.03816395	104	285	12.8%	1.03 [0.95, 1.11]
Tan 2010	0.13720112	0.02803013	15	164	13.7%	1.15 [1.09, 1.21]
**Total (95% CI)**			**327**	**1960**	**100.0%**	**1.12 [1.04, 1.20]**

Heterogeneity: Tau² = 0.01; Chi² = 34.00, df = 14 (P = 0.002); I² = 59%
Test for overall effect: Z = 3.01 (P = 0.003)

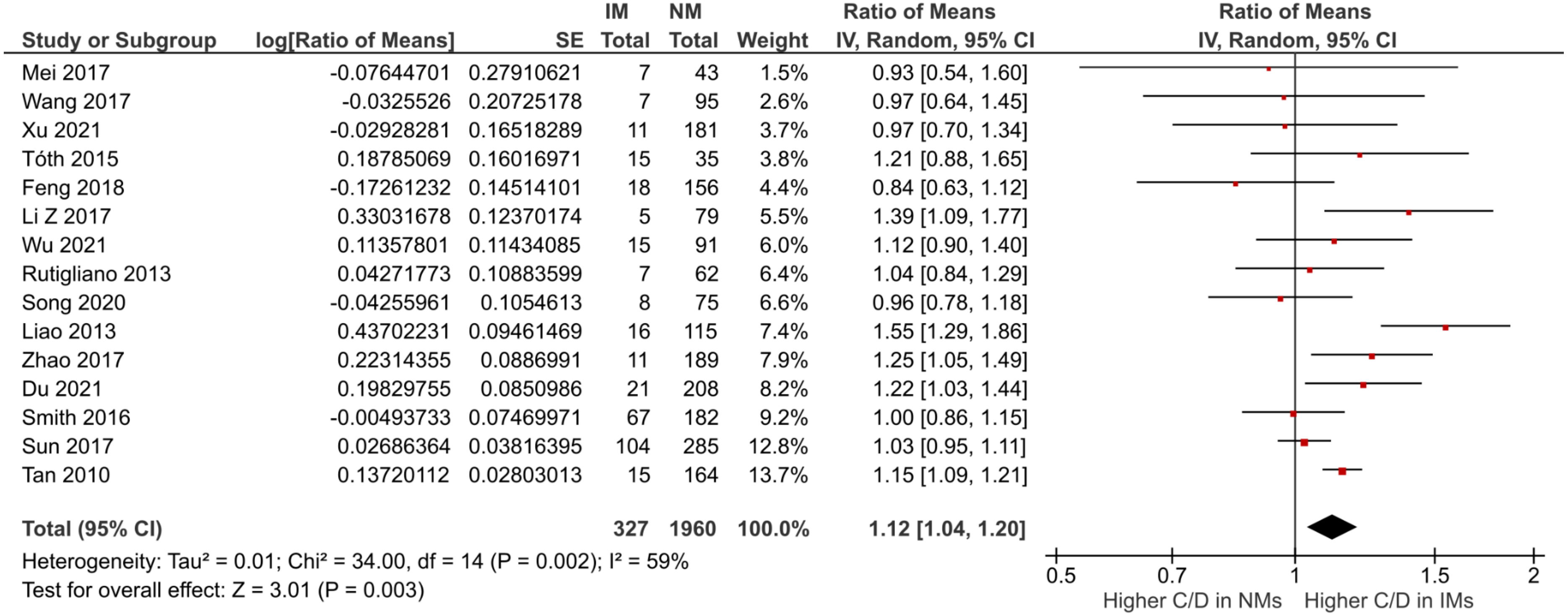

Figure S9. Forest plot (RoM): Valproic acid C/D in CYP2C9 IMs compared to CYP2C9 NMs. Based on the data from k=15 trials and N=2287 patients, CYP2C9 IMs had on average 12% (95%CI: 4%, 20%; df=14; p=0.003) higher C/D than CYP2C9 NMs. Moderate heterogeneity was observed (I^2=59%). *C/D: Plasma concentration-to-dose ratio; IMs: Intermediate metabolizers; NMs: Normal metabolizers (reference group).*

© 2024 Milosavljević F et al. *JAMA Network Open.*

Study or Subgroup	log[Ratio of Means]	SE	IM Total	NM Total	Weight	Ratio of Means IV, Random, 95% CI
Zheng 2023	0.83563846	0.23789968	18	23	3.3%	2.31 [1.45, 3.68]
Mei 2017	0.23087635	0.18643517	23	14	4.6%	1.26 [0.87, 1.82]
Feng 2016	0.23494207	0.15789854	28	18	5.7%	1.26 [0.93, 1.72]
Jogamoto 2017	0.08155774	0.14716034	15	9	6.1%	1.08 [0.81, 1.45]
Smith 2016	-0.00436449	0.10663974	74	96	8.1%	1.00 [0.81, 1.23]
Song 2020	0.28993179	0.10396143	33	37	8.2%	1.34 [1.09, 1.64]
Liao 2013	0.07132478	0.09557645	56	44	8.7%	1.07 [0.89, 1.30]
Feng 2018	0.01082125	0.09013259	85	60	9.0%	1.01 [0.85, 1.21]
Wang 2021	0.36866304	0.05647493	137	118	10.9%	1.45 [1.29, 1.62]
Du 2021	-0.04300144	0.05059016	107	100	11.2%	0.96 [0.87, 1.06]
Sun 2017	-0.05701668	0.03393518	173	177	11.9%	0.94 [0.88, 1.01]
Han 2015	0.00740744	0.02519527	77	72	12.2%	1.01 [0.96, 1.06]
**Total (95% CI)**			**826**	**768**	**100.0%**	**1.12 [1.02, 1.24]**

Heterogeneity: Tau² = 0.02; Chi² = 66.33, df = 11 (P < 0.00001); I² = 83%
Test for overall effect: Z = 2.31 (P = 0.02)

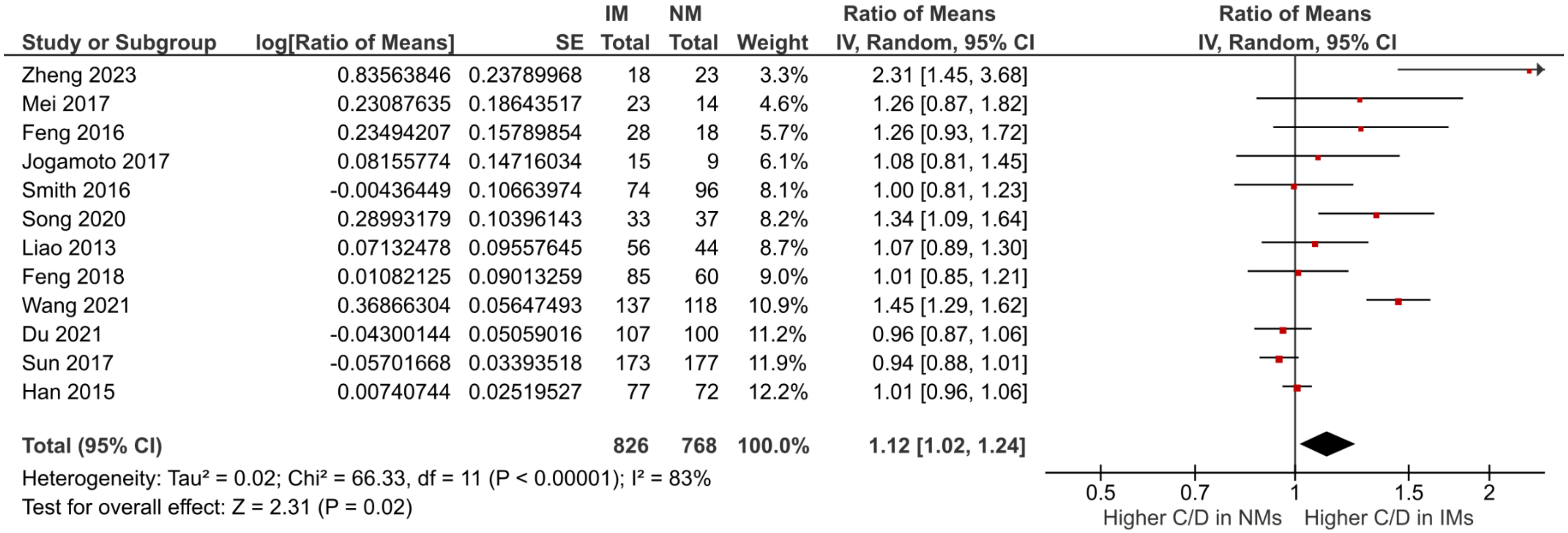

Figure S10. Forest plot (RoM): Valproic acid C/D in CYP2C19 IMs compared to CYP2C19 NMs. Based on the data from k=12 trials and N=1594 patients, CYP2C19 IMs had on average 12% (95%CI: 2%, 24%; df=11; p=0.021) higher C/D than CYP2C19 NMs. High heterogeneity was observed (I^2=83%). *C/D: Plasma concentration-to-dose ratio; IMs: Intermediate metabolizers; NMs: Normal metabolizers (reference group).*

© 2024 Milosavljević F et al. *JAMA Network Open.*

Study or Subgroup	log[Ratio of Means]	SE	PM Total	NM Total	Weight	Ratio of Means IV, Random, 95% CI
Zheng 2023	0.93477226	0.37322687	5	23	3.3%	2.55 [1.23, 5.29]
Mei 2017	0.19363277	0.21396146	10	14	6.1%	1.21 [0.80, 1.85]
Feng 2016	-0.04046488	0.17022803	10	18	7.2%	0.96 [0.69, 1.34]
Jogamoto 2017	-0.2375539	0.14236215	4	9	8.0%	0.79 [0.60, 1.04]
Feng 2018	0.05969571	0.11892191	28	60	8.6%	1.06 [0.84, 1.34]
Liao 2013	0.36442692	0.10104542	15	44	9.0%	1.44 [1.18, 1.76]
Song 2020	0.69013966	0.09840071	13	37	9.1%	1.99 [1.64, 2.42]
Wang 2021	0.44463226	0.08566706	41	118	9.4%	1.56 [1.32, 1.85]
Smith 2016	-0.09269215	0.07860965	9	96	9.5%	0.91 [0.78, 1.06]
Du 2021	-0.03245123	0.0653337	22	100	9.8%	0.97 [0.85, 1.10]
Han 2015	0.34018282	0.05794548	28	72	9.9%	1.41 [1.25, 1.57]
Sun 2017	-0.02934196	0.0543567	51	177	10.0%	0.97 [0.87, 1.08]
**Total (95% CI)**			**236**	**768**	**100.0%**	**1.20 [1.02, 1.41]**

Heterogeneity: Tau² = 0.07; Chi² = 99.13, df = 11 (P < 0.00001); I² = 89%

Test for overall effect: Z = 2.20 (P = 0.03)

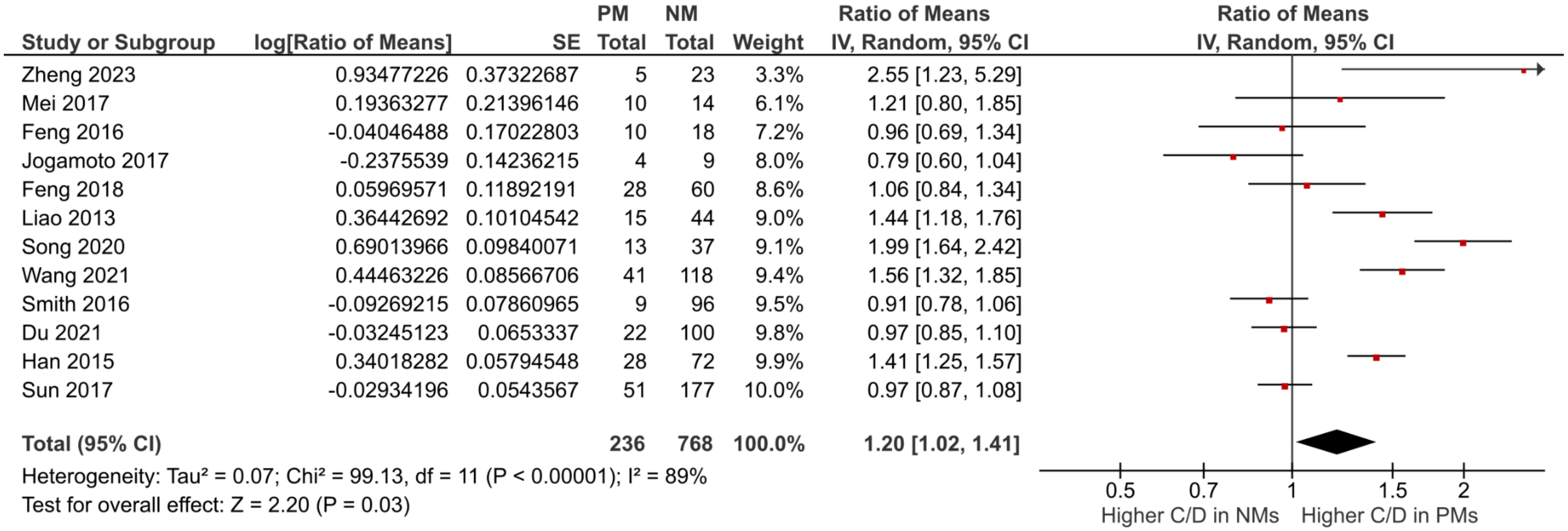

Figure S11. Forest plot (RoM): Valproic acid C/D in CYP2C19 PMs compared to CYP2C19 NMs. Based on the data from k=12 trials and N=1004 patients, CYP2C19 PMs had on average 20% (95%CI: 2%, 41%; df=11; p=0.027) higher C/D than CYP2C19 NMs. High heterogeneity was observed (I^2=89%). *C/D: Plasma concentration-to-dose ratio; PMs: Poor metabolizers; NMs: Normal metabolizers (reference group).*

© 2024 Milosavljević F et al. *JAMA Network Open.*

Study or Subgroup	log[Ratio of means]	SE	*2 He Total	*2 None Total	Weight	Ratio of means IV, Random, 95% CI
Feng 2019	0.32617432	0.21103828	5	20	1.8%	1.39 [0.92, 2.10]
Zheng 2023	-0.85736643	0.2028781	18	25	1.9%	0.42 [0.29, 0.63]
Wang 2019	0.0033215	0.19787983	49	101	1.9%	1.00 [0.68, 1.48]
Jain 2015	0.34333333	0.17873111	31	36	2.2%	1.41 [0.99, 2.00]
Mei 2017	0.03946166	0.17079314	19	24	2.3%	1.04 [0.74, 1.45]
Feng 2016	-0.06431179	0.13346369	19	35	3.1%	0.94 [0.72, 1.22]
Nandith 2021	-0.17366349	0.13205917	61	31	3.1%	0.84 [0.65, 1.09]
Algharably 2016	-0.0724955	0.1080197	16	25	3.7%	0.93 [0.75, 1.15]
Guo 2012	0.09209647	0.10226822	39	49	3.8%	1.10 [0.90, 1.34]
Xu 2021	-0.24968868	0.09910222	70	113	3.9%	0.78 [0.64, 0.95]
Chatzistefanidis 2016	0.03495	0.09810463	66	51	4.0%	1.04 [0.85, 1.26]
Kang 2016	-0.15257959	0.09467829	61	55	4.0%	0.86 [0.71, 1.03]
Li Z 2017	-0.1163897	0.09450722	37	45	4.1%	0.89 [0.74, 1.07]
Shen 2016	-0.11519571	0.09415413	37	55	4.1%	0.89 [0.74, 1.07]
Feng 2018	0.05406503	0.08579132	67	99	4.3%	1.06 [0.89, 1.25]
Zhao 2020	-0.06283058	0.06871784	56	55	4.8%	0.94 [0.82, 1.07]
Sterjev 2012	-0.07807665	0.0668776	61	69	4.8%	0.92 [0.81, 1.05]
Wang 2010	-0.08538517	0.06654178	65	76	4.8%	0.92 [0.81, 1.05]
Jin 2013	-0.3254224	0.06462074	28	69	4.9%	0.72 [0.64, 0.82]
Chu 2012	0.01754431	0.06067099	45	88	5.0%	1.02 [0.90, 1.15]
Hung 2011	-0.35272433	0.05921941	50	101	5.0%	0.70 [0.63, 0.79]
Du 2021	0.02989759	0.04595333	96	120	5.4%	1.03 [0.94, 1.13]
Sun 2017	0.06563716	0.04103306	156	201	5.5%	1.07 [0.99, 1.16]
Aphichartphunkawee 2014	-0.23780071	0.02544599	24	56	5.8%	0.79 [0.75, 0.83]
Sun 2007	-0.23069514	0.01943761	24	40	5.9%	0.79 [0.76, 0.82]
**Total (95% CI)**			**1200**	**1639**	**100.0%**	**0.91 [0.85, 0.97]**

Heterogeneity: Tau² = 0.02; Chi² = 146.00, df = 24 (P < 0.00001); I² = 84%

Test for overall effect: Z = 2.85 (P = 0.004)

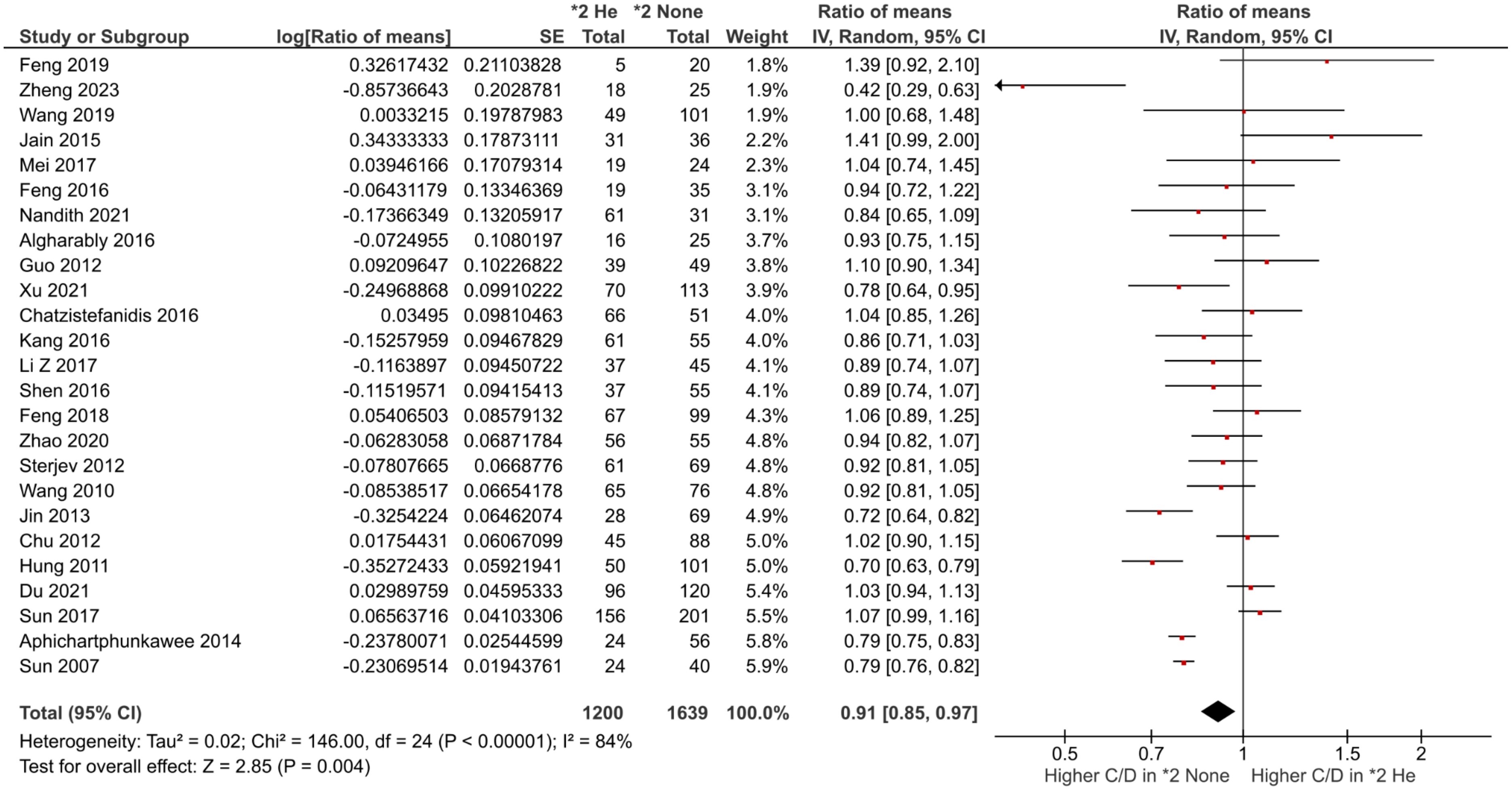

*Figure S12. Forest plot (RoM): Valproic acid C/D in UGT1A6*2 heterozygous (*2 He) carriers and *2 non carriers (*2 None).* Heterozygous carriers were defined as either 19GT, 541AG or 552CA carriers, while *2 non-carriers were defined as either 19TT, 541AA or 552AA carriers. Based on the data from k=25 trials and N=2839 patients, *2 heterozygous carriers had on average lower Valproic acid C/D (fold-change: 0.91 [95%CI: 0.85, 0.97]; df=24; p=0.004) than UGT1A6*2 non-carriers. High heterogeneity was observed (I^2=84%). *C/D: Plasma concentration-to-dose ratio.*

© 2024 Milosavljević F et al. *JAMA Network Open.*

Study or Subgroup	log[Ratio of means]	SE	*2 Ho Total	*2 None Total	Weight	Ratio of means IV, Random, 95% CI
Wang 2019	-0.24953728	0.57054106	7	101	1.1%	0.78 [0.25, 2.38]
Jain 2015	-0.1993329	0.31514966	13	36	2.6%	0.82 [0.44, 1.52]
Li Z 2017	-0.07001921	0.29128105	2	45	2.8%	0.93 [0.53, 1.65]
Xu 2021	0.04889863	0.28899633	10	113	2.8%	1.05 [0.60, 1.85]
Guo 2012	-0.48756876	0.27331466	6	49	3.0%	0.61 [0.36, 1.05]
Feng 2019	0.53031663	0.27302743	3	20	3.0%	1.70 [1.00, 2.90]
Chu 2012	0.26982446	0.26725814	3	88	3.1%	1.31 [0.78, 2.21]
Mei 2017	-0.26537036	0.26037762	4	24	3.2%	0.77 [0.46, 1.28]
Nandith 2021	-0.15468759	0.26017383	7	31	3.2%	0.86 [0.51, 1.43]
Feng 2016	-0.07530191	0.25980855	4	35	3.2%	0.93 [0.56, 1.54]
Feng 2018	0.19390593	0.20671954	8	99	4.0%	1.21 [0.81, 1.82]
Algharably 2016	-0.50849733	0.19664434	7	25	4.2%	0.60 [0.41, 0.88]
Shen 2016	-0.3354365	0.17887279	5	55	4.5%	0.72 [0.50, 1.02]
Zheng 2023	-0.86507796	0.17524118	2	25	4.5%	0.42 [0.30, 0.59]
Kang 2016	0.01869213	0.16183747	13	55	4.8%	1.02 [0.74, 1.40]
Sun 2007	-0.31268337	0.1536652	3	40	4.9%	0.73 [0.54, 0.99]
Chatzistefanidis 2016	0.12495	0.14841995	17	51	5.0%	1.13 [0.85, 1.52]
Wang 2010	-0.04712914	0.1474731	6	76	5.0%	0.95 [0.71, 1.27]
Aphichartphunkawee 2014	0.38227049	0.11957388	4	56	5.6%	1.47 [1.16, 1.85]
Hung 2011	-0.53469821	0.11884869	11	101	5.6%	0.59 [0.46, 0.74]
Zhao 2020	0.06213178	0.11611651	11	55	5.6%	1.06 [0.85, 1.34]
Du 2021	0.0386953	0.11087445	13	120	5.7%	1.04 [0.84, 1.29]
Sterjev 2012	-0.14368739	0.09351182	23	69	6.0%	0.87 [0.72, 1.04]
Sun 2017	0.04663422	0.06211322	38	201	6.5%	1.05 [0.93, 1.18]
**Total (95% CI)**			**220**	**1570**	**100.0%**	**0.90 [0.80, 1.02]**

Heterogeneity: Tau² = 0.06; Chi² = 79.79, df = 23 (P < 0.00001); I² = 71%
Test for overall effect: Z = 1.59 (P = 0.11)

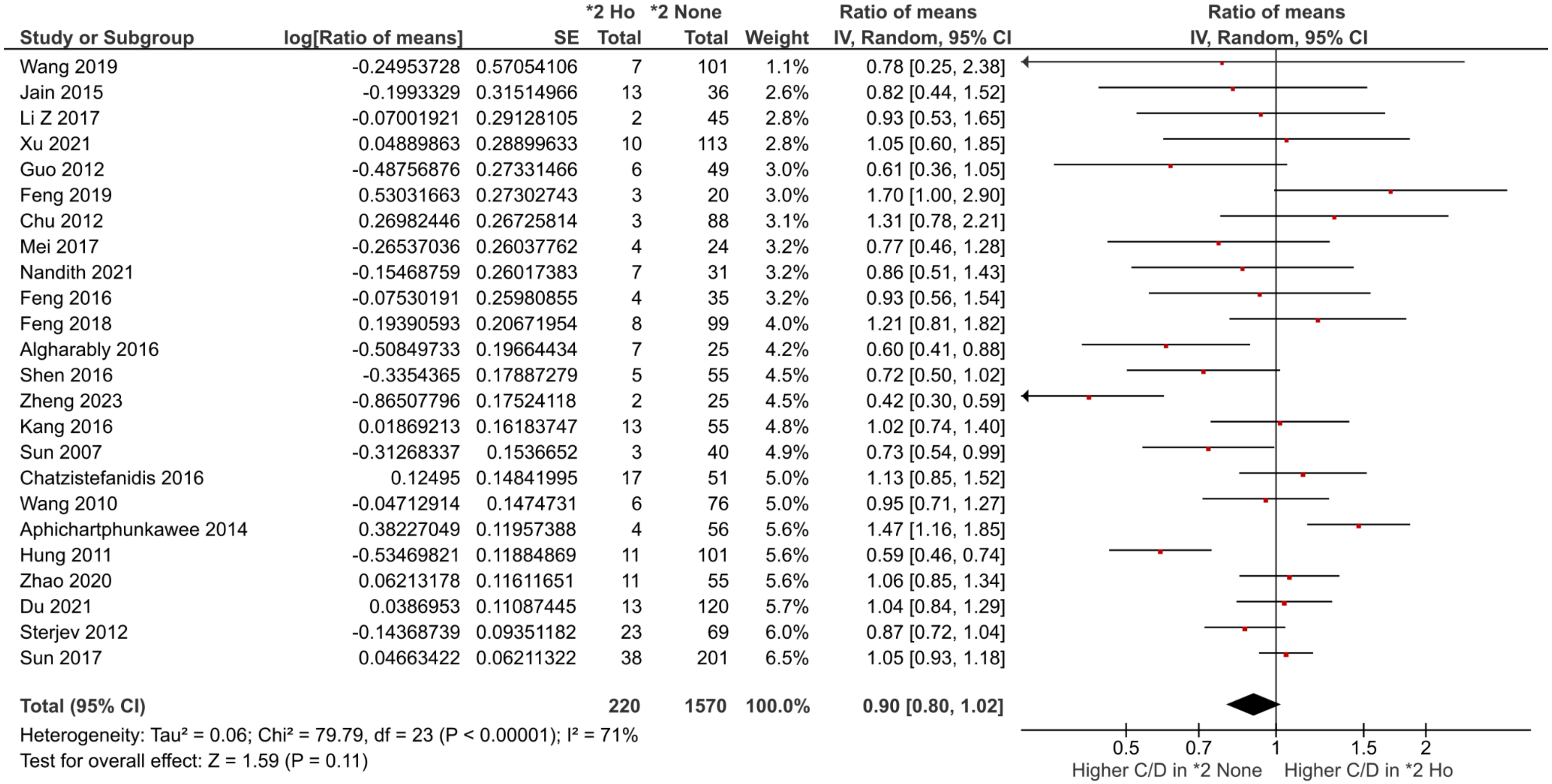

*Figure S13. Forest plot (RoM): Valproic acid C/D in UGT1A6*2 homozygous (*2 Ho) carriers and *2 non carriers (*2 None).* Homozygous carriers were defined as either 19GG, 541GG or 552CC carriers, while *2 non-carriers were defined as either 19TT, 541AA or 552AA carriers. Based on the data from k=24 trials and N=1790 patients, there was no significant differences in Valproic acid C/D between UGT1A6*2 homozygous carriers (fold-change: 0.90 [95%CI: 0.80, 1.02]; df=23; p=0.11) and *2 non-carriers. Moderate-to-high heterogeneity was observed (I^2=71%). *C/D: Plasma concentration-to-dose ratio.*

© 2024 Milosavljević F et al. *JAMA Network Open.*

Study or Subgroup	log[Ratio of Means]	SE	*2 He Total	*2 None Total	Weight	Ratio of Means IV, Random, 95% CI
Zheng 2023	0.03653466	0.32029888	25	15	1.2%	1.04 [0.55, 1.94]
Li Z 2017	0.28927465	0.2813733	66	5	1.5%	1.34 [0.77, 2.32]
Feng 2019	0.14488958	0.26729901	13	11	1.6%	1.16 [0.68, 1.95]
Wang 2019	0.03403832	0.23426911	71	74	2.0%	1.03 [0.65, 1.64]
Chung 2008	0.2726963	0.18236102	5	5	2.8%	1.31 [0.92, 1.88]
Mei 2017	-0.13868399	0.16787974	26	18	3.1%	0.87 [0.63, 1.21]
Sun 2015	-0.35856888	0.14628089	46	14	3.5%	0.70 [0.52, 0.93]
Feng 2016	-0.16092844	0.14061961	27	23	3.7%	0.85 [0.65, 1.12]
Nandith 2021	0.00542988	0.13636362	42	38	3.8%	1.01 [0.77, 1.31]
Guo 2012	0.05258354	0.12139267	31	36	4.2%	1.05 [0.83, 1.34]
Xu 2021	0.15629222	0.11042191	84	94	4.5%	1.17 [0.94, 1.45]
Zhang 2017	-0.07878609	0.10848535	47	60	4.6%	0.92 [0.75, 1.14]
Feng 2018	0.06347687	0.09409271	73	74	5.0%	1.07 [0.89, 1.28]
Chatzistefanidis 2016	0.12	0.09351939	60	74	5.0%	1.13 [0.94, 1.35]
Zhao 2020	0.07589848	0.07615242	63	34	5.6%	1.08 [0.93, 1.25]
Inoue 2014	0.10536052	0.07537188	42	29	5.6%	1.11 [0.96, 1.29]
Wang 2016	0.12429772	0.0749304	84	66	5.6%	1.13 [0.98, 1.31]
Hung 2011	-0.02960514	0.06954181	60	83	5.8%	0.97 [0.85, 1.11]
Wen 2016	0.12136086	0.06561927	61	79	5.9%	1.13 [0.99, 1.28]
Du 2016	-0.48097266	0.06376405	50	11	5.9%	0.62 [0.55, 0.70]
Chu 2012	-0.11908326	0.05557237	60	65	6.2%	0.89 [0.80, 0.99]
Du 2021	-0.08961216	0.048849	106	107	6.4%	0.91 [0.83, 1.01]
Sun 2017	-0.03381965	0.03947929	149	201	6.6%	0.97 [0.89, 1.04]
**Total (95% CI)**			**1291**	**1216**	**100.0%**	**0.99 [0.91, 1.06]**

Heterogeneity: Tau² = 0.02; Chi² = 86.69, df = 22 (P < 0.00001); I² = 75%
Test for overall effect: Z = 0.35 (P = 0.72)

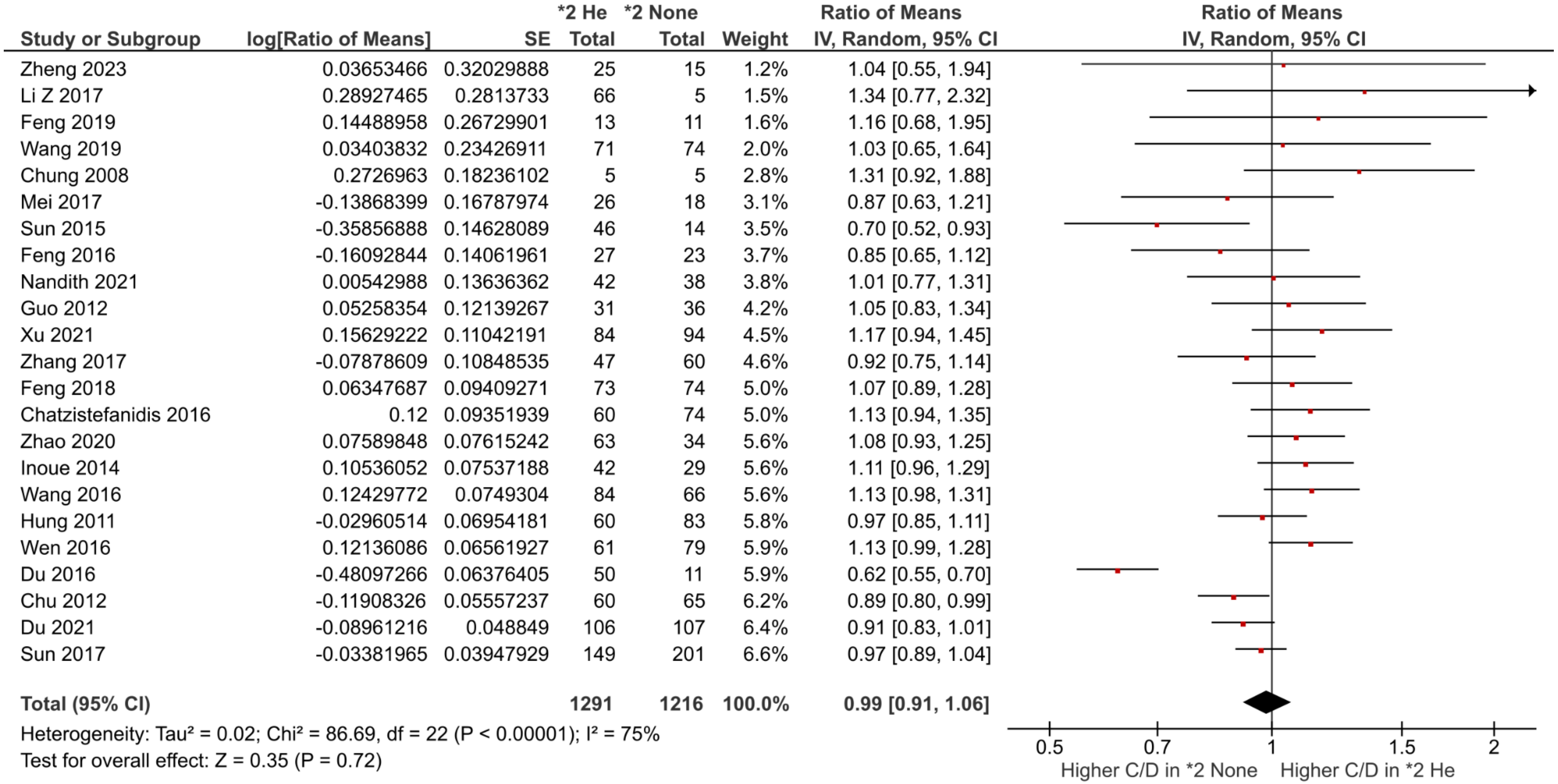

*Figure S14. Forest plot (RoM): Valproic acid C/D in UGT2B7*2 heterozygous (*2 He) carriers and *2 non carriers (*2 None).* Heterozygous carriers were defined as either 802CT or -161CT carriers, while *2 non-carriers were defined as either 802CC or -161CC carriers. Based on the data from k=23 trials and N=2507 patients, there was no significant difference in Valproic acid C/D between *2 heterozygous carriers and *2 non-carriers (fold-change: 0.99 [95%CI: 0.91, 1.06]; df=22; p=0.72). High heterogeneity was observed (I^2=75%). *C/D: Plasma concentration-to-dose ratio.*

© 2024 Milosavljević F et al. *JAMA Network Open.*

Study or Subgroup	log[Ratio of Means]	SE	*2 Ho Total	*2 None Total	Weight	Ratio of Means IV, Random, 95% CI
Zheng 2023	-0.29825549	0.37415354	6	15	1.3%	0.74 [0.36, 1.55]
Li Z 2017	0.08687073	0.32228609	13	5	1.7%	1.09 [0.58, 2.05]
Wang 2019	0.31598012	0.3083089	12	74	1.8%	1.37 [0.75, 2.51]
Feng 2019	-0.11817442	0.2567806	5	11	2.4%	0.89 [0.54, 1.47]
Xu 2021	0.18277621	0.20610754	13	94	3.2%	1.20 [0.80, 1.80]
Chung 2008	0.39609642	0.20579109	4	5	3.2%	1.49 [0.99, 2.22]
Feng 2016	-0.26203286	0.20129745	8	23	3.3%	0.77 [0.52, 1.14]
Guo 2012	0.00234467	0.15591686	15	36	4.4%	1.00 [0.74, 1.36]
Mei 2017	-0.10478293	0.15527711	3	18	4.4%	0.90 [0.66, 1.22]
Zhang 2017	-0.41560208	0.1529175	10	60	4.4%	0.66 [0.49, 0.89]
Sun 2015	-0.26800931	0.14358916	42	14	4.7%	0.76 [0.58, 1.01]
Inoue 2014	0.29905506	0.13783855	7	29	4.9%	1.35 [1.03, 1.77]
Nandith 2021	0.01262415	0.13175866	19	38	5.1%	1.01 [0.78, 1.31]
Wen 2016	-0.25593337	0.12909525	9	79	5.1%	0.77 [0.60, 1.00]
Chu 2012	0.00621764	0.12458919	11	65	5.3%	1.01 [0.79, 1.28]
Wang 2016	0.31309179	0.11578236	16	66	5.6%	1.37 [1.09, 1.72]
Feng 2018	0.10417289	0.11065229	26	74	5.7%	1.11 [0.89, 1.38]
Hung 2011	0.29942803	0.10773325	19	83	5.8%	1.35 [1.09, 1.67]
Du 2021	-0.11332869	0.10704365	16	107	5.9%	0.89 [0.72, 1.10]
Zhao 2020	0.22957444	0.08322658	25	34	6.7%	1.26 [1.07, 1.48]
Sun 2017	-0.12643077	0.06239316	45	201	7.3%	0.88 [0.78, 1.00]
Du 2016	-0.0993182	0.04147231	41	11	7.9%	0.91 [0.83, 0.98]
**Total (95% CI)**			**365**	**1142**	**100.0%**	**1.01 [0.92, 1.11]**

Heterogeneity: Tau² = 0.03; Chi² = 61.94, df = 21 (P < 0.00001); I² = 66%
Test for overall effect: Z = 0.20 (P = 0.84)

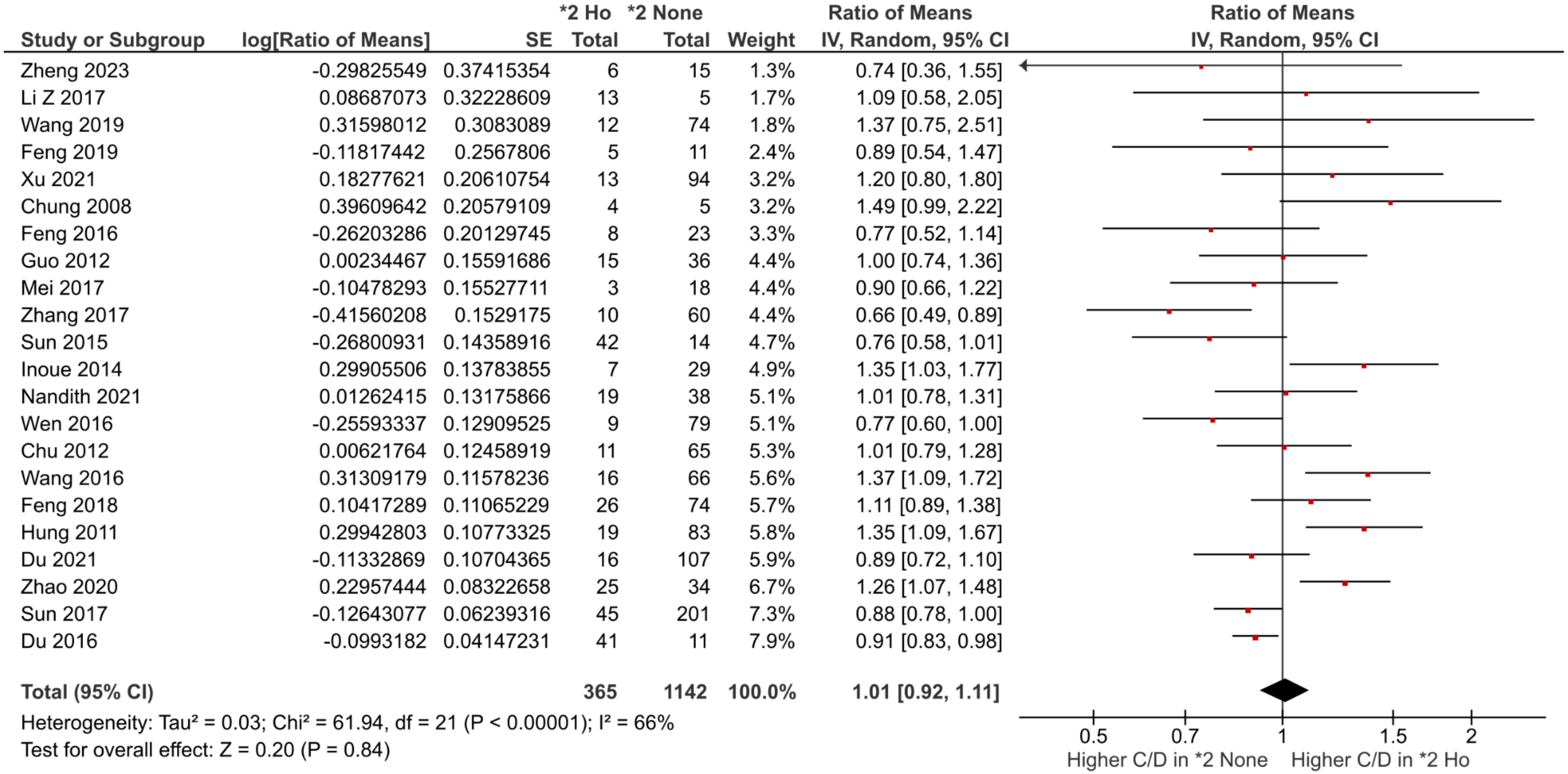

*Figure S15. Forest plot (RoM): Valproic acid C/D in UGT2B7*2 homozygous (*2 Ho) carriers and *2 non carriers (*2 None).* Homozygous carriers were defined as either 802TT or -161TT carriers, while *2 non-carriers were defined as either 802CC or -161CC carriers. Based on the data from k=22 trials and N=1507 patients, there was no significant difference in Valproic acid C/D between *2 homozygous carriers and *2 non-carriers (fold-change: 1.01 [95%CI: 0.92, 1.11]; df=21; p=0.84). Moderate heterogeneity was observed (I^2=66%). *C/D: Plasma concentration-to-dose ratio.*

© 2024 Milosavljević F et al. *JAMA Network Open.*

Study or Subgroup	log[Ratio of Mean]	SE	*3 He Total	*3 None Total	Weight	Ratio of Mean IV, Random, 95% CI
Feng 2019	-0.33061687	0.38404665	6	20	0.3%	0.72 [0.34, 1.53]
Zheng 2023	0.02095784	0.33501544	10	35	0.4%	1.02 [0.53, 1.97]
Mei 2017	-0.24173452	0.23827156	9	37	0.9%	0.79 [0.49, 1.25]
Sun 2015	0.01003718	0.14109785	23	78	2.5%	1.01 [0.77, 1.33]
Nandith 2021	0.25152484	0.13907048	17	80	2.5%	1.29 [0.98, 1.69]
Shen 2016	-0.06364527	0.11358754	23	70	3.8%	0.94 [0.75, 1.17]
Xu 2021	-0.13633644	0.11154428	49	137	4.0%	0.87 [0.70, 1.09]
Ma 2013	-0.05047624	0.10591371	90	147	4.4%	0.95 [0.77, 1.17]
Du 2016	-0.08061235	0.09809701	26	74	5.1%	0.92 [0.76, 1.12]
Feng 2018	0.06042289	0.09296648	43	126	5.7%	1.06 [0.89, 1.27]
Wang 2016	-0.1188612	0.08493467	45	119	6.8%	0.89 [0.75, 1.05]
Du 2021	-0.04580954	0.0487662	44	181	20.7%	0.96 [0.87, 1.05]
Sun 2017	-0.00950126	0.03386866	121	256	42.9%	0.99 [0.93, 1.06]
**Total (95% CI)**			**506**	**1360**	**100.0%**	**0.97 [0.93, 1.01]**

Heterogeneity: Tau² = 0.00; Chi² = 9.41, df = 12 (P = 0.67); I² = 0%
Test for overall effect: Z = 1.30 (P = 0.19)

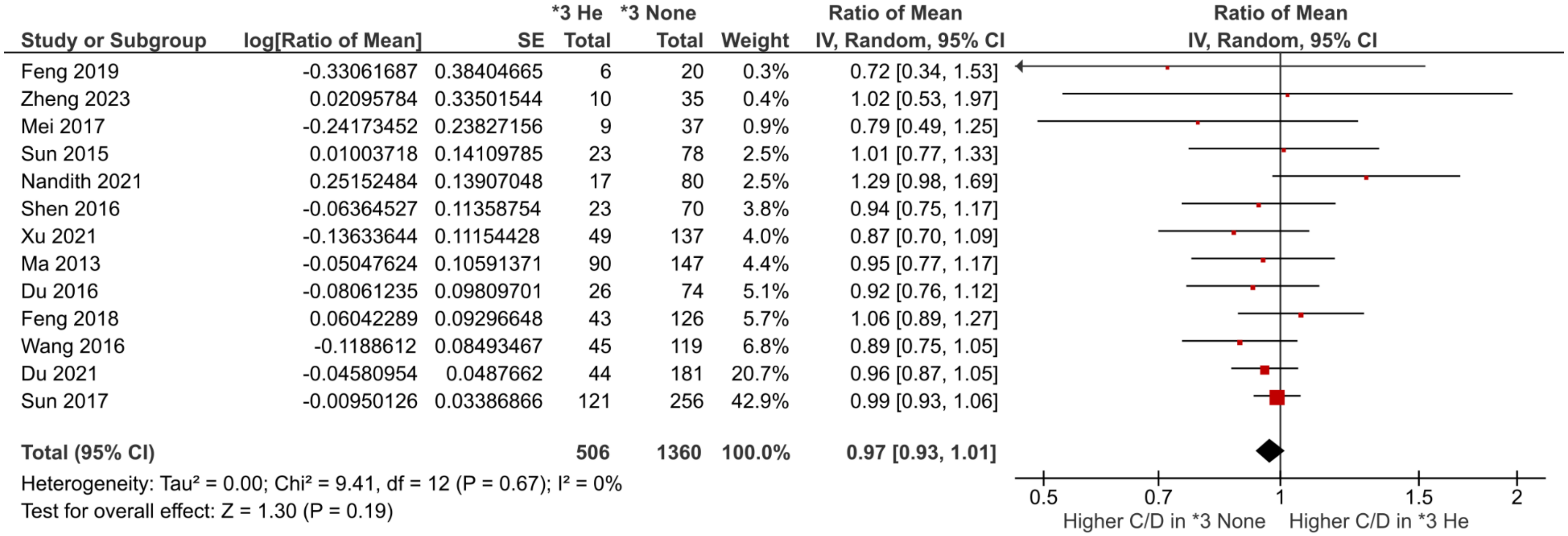

*Figure S16. Forest plot (RoM): Valproic acid C/D in UGT2B7*3 heterozygous (*3 He) carriers and *3 non carriers (*3 None).* Heterozygous carriers were defined as 211GT carriers, while *3 non-carriers were defined as 211GG carriers. Based on the data from k=13 trials and N=1866 patients, there was no significant difference in Valproic acid C/D between *3 heterozygous carriers and *3 non-carriers (fold-change: 0.97 [95%CI: 0.93, 1.01]; df=12; p=0.19). No heterogeneity was observed (I^2=0%). *C/D: Plasma concentration-to-dose ratio.*

© 2024 Milosavljević F et al. *JAMA Network Open.*

Study or Subgroup	log[Ratio of Mean]	SE	*3 Ho Total	*3 None Total	Weight	Ratio of Mean IV, Random, 95% CI
Sun 2017	0.05417271	0.40254425	3	126	8.4%	1.06 [0.48, 2.32]
Du 2021	0.27717935	0.39903117	4	181	8.5%	1.32 [0.60, 2.88]
Feng 2018	0.42247645	0.36076203	5	137	9.6%	1.53 [0.75, 3.09]
Feng 2019	-0.52448798	0.33882834	3	20	10.4%	0.59 [0.30, 1.15]
Ma 2013	-0.76999241	0.18874654	11	147	17.6%	0.46 [0.32, 0.67]
Shen 2016	-0.34942439	0.09797255	4	70	22.7%	0.71 [0.58, 0.85]
Xu 2021	-0.02392459	0.09745308	17	256	22.8%	0.98 [0.81, 1.18]
**Total (95% CI)**			**47**	**937**	**100.0%**	**0.81 [0.62, 1.08]**

Heterogeneity: Tau² = 0.08; Chi² = 20.27, df = 6 (P = 0.002); I² = 70%
Test for overall effect: Z = 1.45 (P = 0.15)

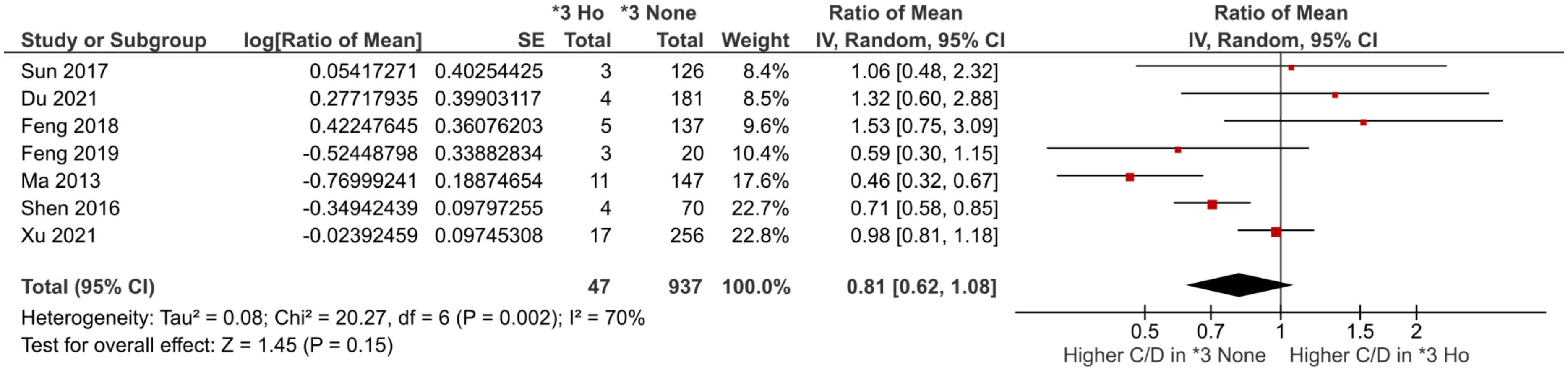

*Figure S17. Forest plot (RoM): Valproic acid C/D in UGT2B7*3 homozygous (*3 Ho) carriers and *3 non carriers (*3 None).* Homozygous carriers were defined as 211TT carriers, while *3 non-carriers were defined as 211GG carriers. Based on the data from k=7 trials and N=984 patients, there was no significant difference in Valproic acid C/D between *3 homozygous carriers and *3 non-carriers (fold-change: 0.81 [95%CI: 0.62, 1.08]; df=6; p=0.15). Moderate heterogeneity was observed (I^2=70%). *C/D: Plasma concentration-to-dose ratio.*

© 2024 Milosavljević F et al. *JAMA Network Open.*

3.3) Lamotrigine related RoM meta-analyses (Figures S18-S20)

Study or Subgroup	log[Ratio of Means]	SE	Experimental Total	Control Total	Weight	Ratio of Means IV, Random, 95% CI
Suzuki 2019b	-0.55961579	0.26633843	5	13	5.1%	0.57 [0.34, 0.96]
Petrenaite 2018	-0.01769958	0.24109322	8	25	5.5%	0.98 [0.61, 1.58]
Ortega-Vázquez 2020	-0.09984597	0.19129005	20	58	6.3%	0.90 [0.62, 1.32]
Suzuki 2019a	-0.11466291	0.18092859	14	22	6.5%	0.89 [0.63, 1.27]
Liu 2015a	0.32158362	0.15959435	22	34	6.8%	1.38 [1.01, 1.89]
Reimers 2016	-0.15415068	0.15268494	19	127	6.9%	0.86 [0.64, 1.16]
Chang 2014	0.66042889	0.14204534	24	82	7.1%	1.94 [1.47, 2.56]
Wang 2015	0.22746321	0.10675754	46	102	7.6%	1.26 [1.02, 1.55]
Božina 2023	-0.05302788	0.08410375	106	365	7.9%	0.95 [0.80, 1.12]
Petrenaite 2022	0.037179	0.07368177	38	157	8.0%	1.04 [0.90, 1.20]
Du 2016	-0.24708702	0.07278138	28	74	8.0%	0.78 [0.68, 0.90]
Liu 2015b	0.25079508	0.06821924	49	109	8.1%	1.29 [1.12, 1.47]
He 2017	0.14632971	0.06298238	33	39	8.1%	1.16 [1.02, 1.31]
Smith 2018	-0.60567595	0.06122187	87	447	8.1%	0.55 [0.48, 0.62]
**Total (95% CI)**			**499**	**1654**	**100.0%**	**0.99 [0.82, 1.20]**

Heterogeneity: Tau² = 0.11; Chi² = 161.79, df = 13 (P < 0.00001); I² = 92%
Test for overall effect: Z = 0.06 (P = 0.95)

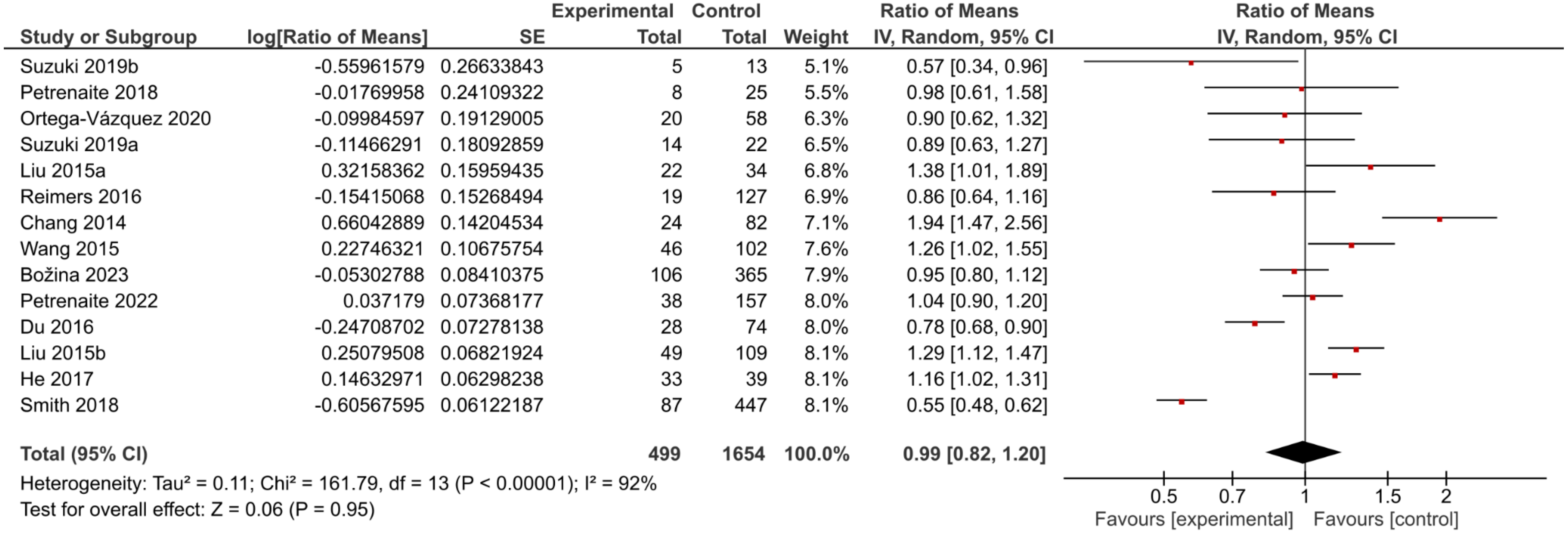

*Figure S18. Forest plot (RoM): Lamotrigine C/D in UGT1A4*3 non-carriers compared to UGT1A4*3 carriers.* Based on the data from 14 cohorts from k=12 trials and N=2153 patients, on the average there was no significant difference in lamotrigine C/D between UGT1A4*3 carriers and non-carriers (fold-change: 0.99 [95%CI: 0.82, 1.20]; df=13; p=0.95). Very high level of heterogeneity was observed (I^2=92%). *C/D: Plasma concentration-to-dose ratio.*

© 2024 Milosavljević F et al. *JAMA Network Open.*

Study or Subgroup	log[Ratio of Means]	SE	Experimental Total	Control Total	Weight	Ratio of Means IV, Random, 95% CI
Suzuki 2019b	0.55961579	0.26633843	13	5	1.9%	1.75 [1.04, 2.95]
Suzuki 2019a	0.11466291	0.18092859	22	14	4.0%	1.12 [0.79, 1.60]
Ortega-Vázquez 2020	-0.21503383	0.16173966	35	60	5.0%	0.81 [0.59, 1.11]
Petrenaite 2018	0.05715841	0.14847847	18	7	5.9%	1.06 [0.79, 1.42]
Petrenaite 2022	-0.02396919	0.0873901	105	42	15.6%	0.98 [0.82, 1.16]
Božina 2023	0.00477222	0.08239355	237	119	17.2%	1.00 [0.85, 1.18]
Wang 2016	0.01639381	0.07923434	84	66	18.4%	1.02 [0.87, 1.19]
Zhou 2015	0.08025693	0.05602346	55	77	32.0%	1.08 [0.97, 1.21]
**Total (95% CI)**			**569**	**390**	**100.0%**	**1.03 [0.96, 1.11]**

Heterogeneity: Tau² = 0.00; Chi² = 7.78, df = 7 (P = 0.35); I² = 10%
Test for overall effect: Z = 0.91 (P = 0.36)

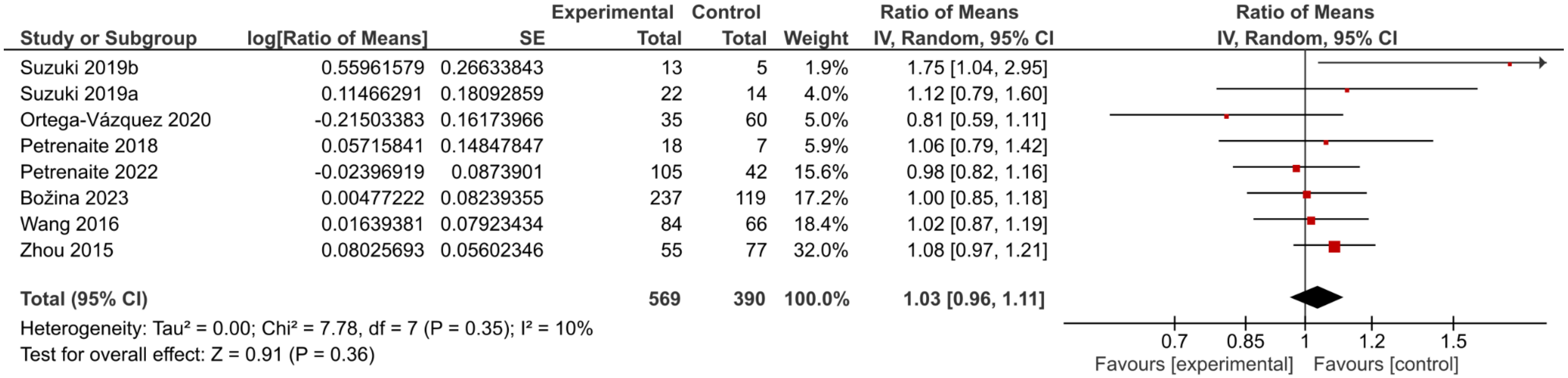

*Figure S19. Forest plot (RoM): Lamotrigine C/D in UGT2B7*2 heterozygous (*2 He) carriers and *2 non carriers (*2 None).* Heterozygous carriers were defined as either 802CT or -161CT carriers, while *2 non-carriers were defined as either 802CC or -161CC carriers. Based on the data from 8 cohorts from k=7 trials and N=959 patients, there was no significant difference in lamotrigine C/D between *2 heterozygous carriers and *2 non-carriers (fold-change: 1.03 [95%CI: 0.96, 1.11]; df=7; p=0.36). Low heterogeneity was observed (I^2=10%). *C/D: Plasma concentration-to-dose ratio.*

Study or Subgroup	log[Ratio of Means]	SE	Experimental Total	Control Total	Weight	Ratio of Means IV, Random, 95% CI
Petrenaite 2018	0.31666961	0.20096846	8	7	13.8%	1.37 [0.93, 2.04]
Zhou 2015	0.27577293	0.18373884	8	77	15.2%	1.32 [0.92, 1.89]
Wang 2016	-0.23144235	0.12502371	16	66	21.4%	0.79 [0.62, 1.01]
Božina 2023	0.00479805	0.09952443	115	119	24.5%	1.00 [0.83, 1.22]
Petrenaite 2022	0.20829704	0.09496483	52	42	25.1%	1.23 [1.02, 1.48]
**Total (95% CI)**			**199**	**311**	**100.0%**	**1.09 [0.90, 1.32]**

Heterogeneity: Tau² = 0.03; Chi² = 11.12, df = 4 (P = 0.03); I² = 64%
Test for overall effect: Z = 0.92 (P = 0.36)

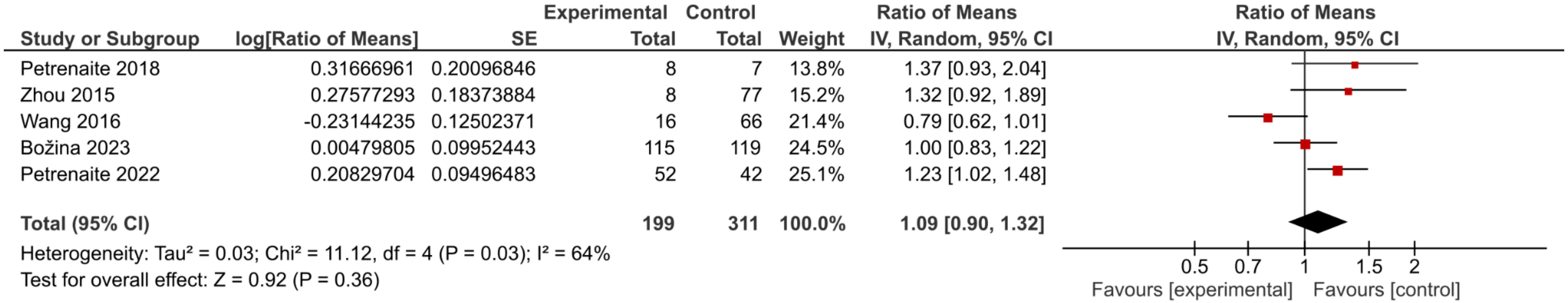

*Figure S20. Forest plot (RoM): Lamotrigine C/D in UGT2B7*2 homozygous (*2 Ho) carriers and *2 non carriers (*2 None).* Homozygous carriers were defined as either 802TT or -161TT carriers, while *2 non-carriers were defined as either 802CC or -161CC carriers. Based on the data from k=5 trials and N=510 patients, there was no significant difference in lamotrigine C/D between *2 homozygous carriers and *2 non-carriers (fold-change: 1.09 [95%CI: 0.90, 1.32]; df=4; p=0.36). Moderate heterogeneity was observed (I^2=64%). *C/D: Plasma concentration-to-dose ratio.*

© 2024 Milosavljević F et al. *JAMA Network Open.*

3.4) Carbamazepine related RoM meta-analyses (Figures S21-S26)

Study or Subgroup	log[Ratio of mean]	SE	CYP3A5 *3/*3 Total	CYP3A5 Expressor Total	Weight	Ratio of mean IV, Random, 95% CI
Pham 2020	0.0250013	0.20226229	6	32	3.3%	1.03 [0.69, 1.52]
Liu 2016	-0.2422197	0.1884359	32	35	3.7%	0.78 [0.54, 1.14]
Ganesapandian 2019	0.46661953	0.18429467	31	69	3.8%	1.59 [1.11, 2.29]
Lu 2018	0.01368269	0.14216438	33	29	5.4%	1.01 [0.77, 1.34]
Liu 2014	0.35667494	0.12536694	13	27	6.3%	1.43 [1.12, 1.83]
Park 2009	0.28007747	0.11850675	21	14	6.7%	1.32 [1.05, 1.67]
Panomvana 2013	-0.04153804	0.11777821	15	21	6.7%	0.96 [0.76, 1.21]
Wang 2015	0.14128609	0.09895182	32	34	7.9%	1.15 [0.95, 1.40]
Zhu 2014	0.17677626	0.06669028	132	78	10.4%	1.19 [1.05, 1.36]
Seo 2006	-0.03376848	0.06619096	75	69	10.5%	0.97 [0.85, 1.10]
Meng 2011	0.27931382	0.05961507	52	32	11.0%	1.32 [1.18, 1.49]
Saruwatari 2014	0.04717444	0.04700644	58	46	12.0%	1.05 [0.96, 1.15]
Ma 2015	0.01937906	0.04238911	80	86	12.4%	1.02 [0.94, 1.11]
**Total (95% CI)**			**580**	**572**	**100.0%**	**1.12 [1.03, 1.22]**

Heterogeneity: Tau² = 0.01; Chi² = 35.14, df = 12 (P = 0.0004); I² = 66%
Test for overall effect: Z = 2.70 (P = 0.007)

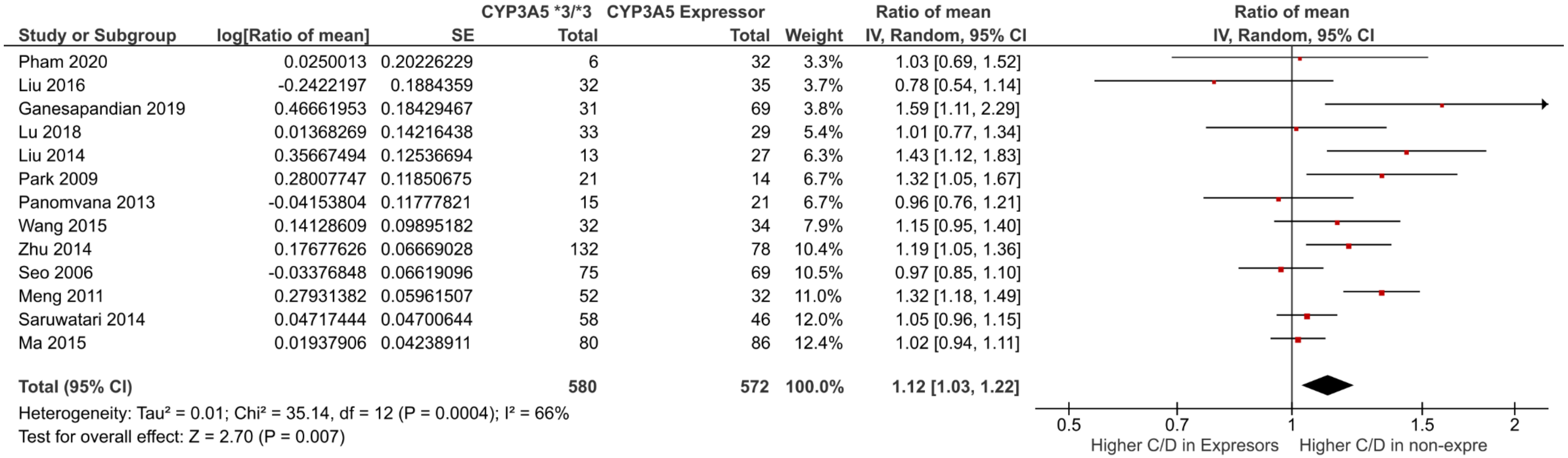

Figures S21. Forest plot (RoM): Carbamazepine active moiety C/D in CYP3A5 non-expressors and expressors. CYP3A5 non-expressors were defined as the carriers of *3/*3 genotype, while every other participant was classified as CYP3A5 expressor. Based on the data from k=13 trials and N=1152 patients, CYP3A5 non-expressors had on average 12% higher carbamazepine active moiety C/D (95%CI: 3%, 22%; df=12; p=0.007) compared to CYP3A5 expressors. Moderate heterogeneity was observed (I^2=66%). *C/D: Plasma concentration-to-dose ratio.*

© 2024 Milosavljević F et al. *JAMA Network Open.*

Study or Subgroup	log[Ratio of mean]	SE	337CT Total	337TT Total	Weight	Ratio of mean IV, Random, 95% CI
Venkatraman 2023	0.21872909	0.1308097	63	35	11.9%	1.24 [0.96, 1.61]
Yun 2013	-0.04445176	0.12532248	37	19	12.2%	0.96 [0.75, 1.22]
Chbili 2016	0.01036279	0.1067575	16	78	13.4%	1.01 [0.82, 1.25]
Daci 2015	0.05505978	0.0975264	72	63	14.0%	1.06 [0.87, 1.28]
Zhu 2014	-0.10135249	0.06933622	115	68	15.8%	0.90 [0.79, 1.04]
Hung 2012	-0.43325	0.06695374	113	58	15.9%	0.65 [0.57, 0.74]
Ma 2015	-0.22066063	0.05004576	81	51	16.8%	0.80 [0.73, 0.88]
**Total (95% CI)**			**497**	**372**	**100.0%**	**0.91 [0.78, 1.06]**

Heterogeneity: Tau² = 0.03; Chi² = 35.45, df = 6 (P < 0.00001); I² = 83%
Test for overall effect: Z = 1.20 (P = 0.23)

Ratio of mean
IV, Random, 95% CI

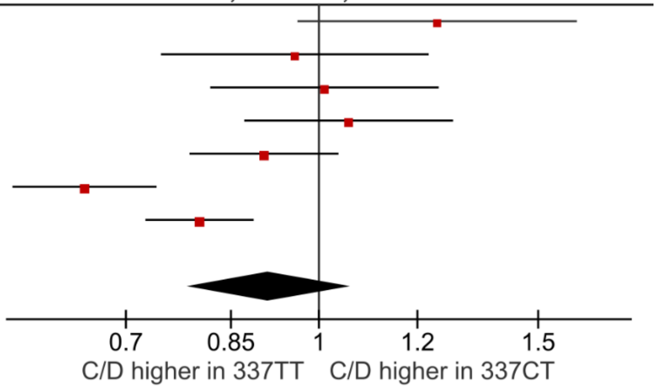

Figure S22. Forest plot (RoM): Carbamazepine active moiety C/D in EPHX1 337CT and 337TT carriers. Carriers of 337CT genotype are heterozygous carriers of mutated allele while 337TT carriers are considered the controls. Based on the data from k=7 trials and N=869 patients, there was no significant differences in Carbamazepine active moiety C/D between 337CT and 337TT carriers (fold-change: 0.91 [95%CI: 0.78, 1.06]; df=6; p=0.23). High heterogeneity was observed (I^2=83%). *C/D: Plasma concentration-to-dose ratio.*

© 2024 Milosavljević F et al. *JAMA Network Open.*

Study or Subgroup	log[Ratio of mean]	SE	337CC Total	337TT Total	Weight	Ratio of mean IV, Random, 95% CI
Venkatraman 2023	0.16597505	0.3100182	17	35	10.2%	1.18 [0.64, 2.17]
Yun 2013	-0.17022115	0.12658222	27	19	14.4%	0.84 [0.66, 1.08]
Zhu 2014	-0.02439145	0.11703578	27	68	14.6%	0.98 [0.78, 1.23]
Daci 2015	-0.24459849	0.09562234	10	63	15.0%	0.78 [0.65, 0.94]
Hung 2012	-0.6464	0.07980377	63	58	15.2%	0.52 [0.45, 0.61]
Chbili 2016	0.58314629	0.07637574	24	78	15.2%	1.79 [1.54, 2.08]
Ma 2015	-0.10025075	0.05687103	34	51	15.4%	0.90 [0.81, 1.01]
**Total (95% CI)**			**202**	**372**	**100.0%**	**0.93 [0.67, 1.29]**

Heterogeneity: Tau² = 0.18; Chi² = 130.21, df = 6 (P < 0.00001); I² = 95%
Test for overall effect: Z = 0.43 (P = 0.66)

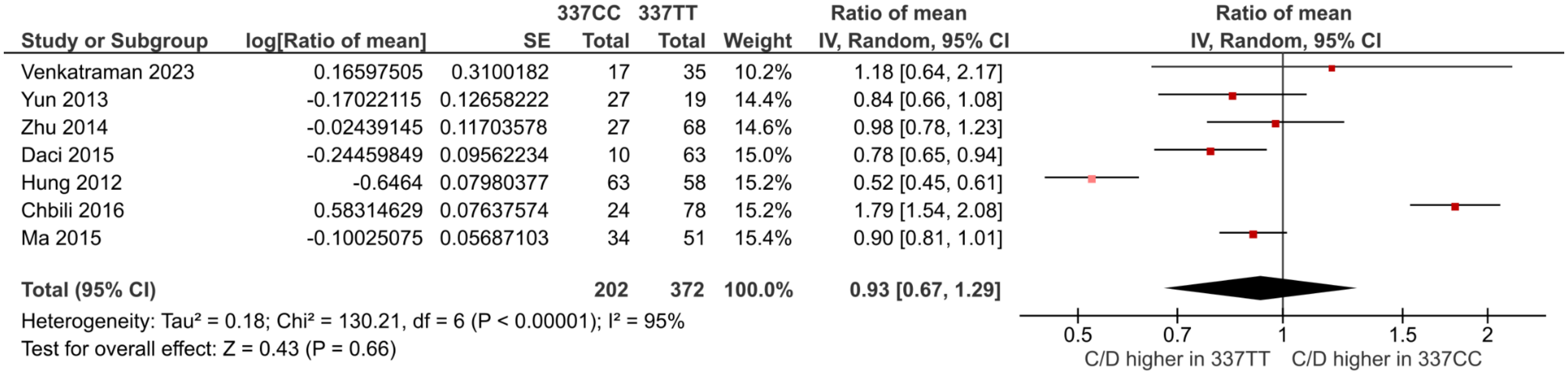

Figure S23. Forest plot (RoM): Carbamazepine active moiety C/D in EPHX1 337CC and 337TT carriers. Carriers of 337CC genotype are homozygous carriers of mutated allele while 337TT carriers are considered the controls. Based on the data from k=7 trials and N=574 patients, there was no significant differences in Carbamazepine active moiety C/D between 337CC and 337TT carriers (fold-change: 0.93 [95%CI: 0.67, 1.29]; df=6; p=0.66). High heterogeneity was observed (I^2=95%). *C/D: Plasma concentration-to-dose ratio.*

© 2024 Milosavljević F et al. *JAMA Network Open.*

Study or Subgroup	log[Ratio of mean]	SE	416AA Total	416AG Total	Weight	Ratio of mean IV, Random, 95% CI
Yun 2013	0.29266961	0.11403373	17	64	15.0%	1.34 [1.07, 1.68]
Daci 2015	-0.08376988	0.10013242	55	80	17.4%	0.92 [0.76, 1.12]
Zhu 2014	-0.06624939	0.08140033	36	172	21.3%	0.94 [0.80, 1.10]
Chbili 2016	-0.02083409	0.07693985	13	98	22.4%	0.98 [0.84, 1.14]
Hung 2012	0.0652	0.07081301	55	176	23.9%	1.07 [0.93, 1.23]
**Total (95% CI)**			**176**	**590**	**100.0%**	**1.03 [0.92, 1.15]**

Heterogeneity: Tau² = 0.01; Chi² = 8.60, df = 4 (P = 0.07); I² = 54%
Test for overall effect: Z = 0.46 (P = 0.65)

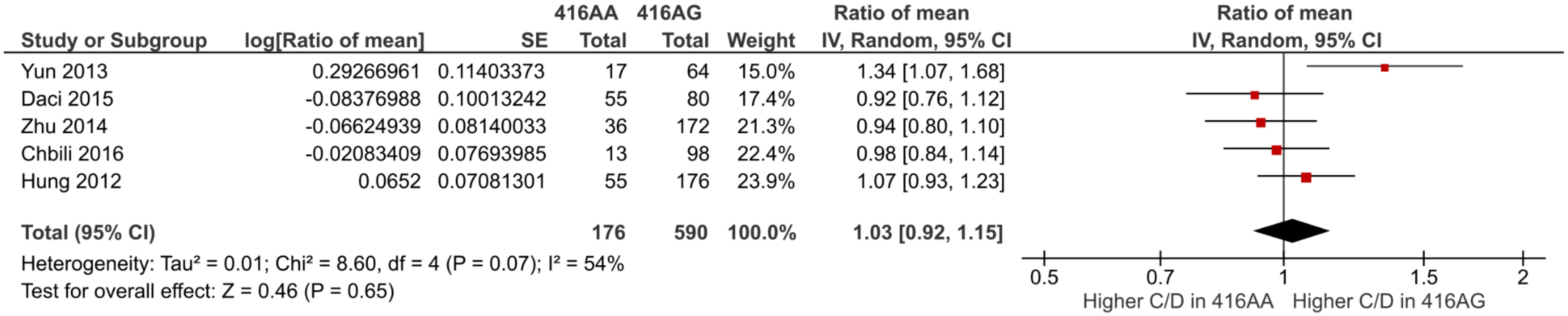

Figure S24. Forest plot (RoM): Carbamazepine active moiety C/D in EPHX1 416AA and 416AG carriers. Carriers of 416AG genotype are heterozygous carriers of mutated allele while 416AA carriers are considered the controls. Based on the data from k=5 trials and N=766 patients, there was no significant differences in Carbamazepine active moiety C/D between 416AA and 416AG carriers (fold-change: 1.03 [95%CI: 0.92, 1.15]; df=4; p=0.65). Low-to-moderate heterogeneity was observed (I^2=54%). No reliable meta-analysis on 416GG carriers was not possible due to low amount available data. *C/D: Plasma concentration-to-dose ratio.*

© 2024 Milosavljević F et al. *JAMA Network Open.*

Study or Subgroup	log[Ratio of mean]	SE	UGT2B7 *2 He Total	UGT2B7 *2 None Total	Weight	Ratio of mean IV, Random, 95% CI
Venkatraman 2023	-0.16368431	0.16542425	47	49	8.3%	0.85 [0.61, 1.17]
Lu 2018	-0.29721748	0.1273891	32	28	12.6%	0.74 [0.58, 0.95]
Zhang 2018	-0.08939761	0.11093351	30	40	15.3%	0.91 [0.74, 1.14]
Hung 2012	0.05125	0.06306137	84	133	28.3%	1.05 [0.93, 1.19]
Ma 2015	0.00107312	0.04451766	79	68	35.5%	1.00 [0.92, 1.09]
**Total (95% CI)**			**272**	**318**	**100.0%**	**0.95 [0.86, 1.05]**

Heterogeneity: Tau² = 0.01; Chi² = 7.38, df = 4 (P = 0.12); I² = 46%
Test for overall effect: Z = 0.95 (P = 0.34)

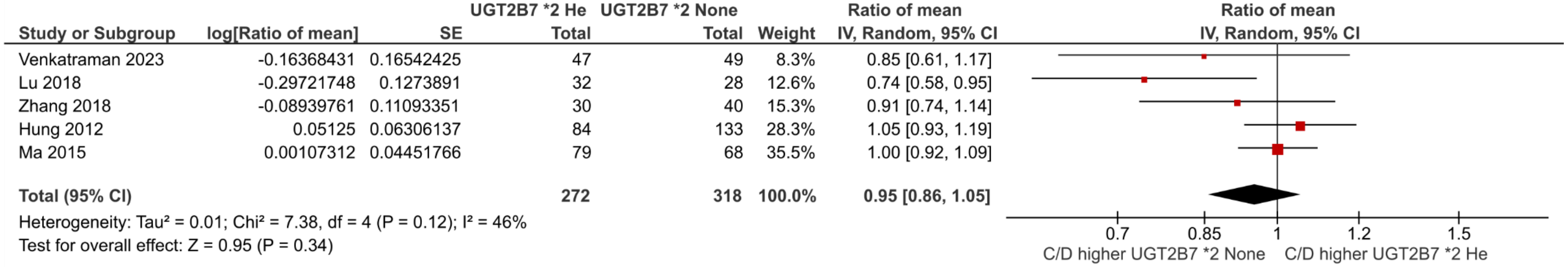

*Figure S25. Forest plot (RoM): Carbamazepine active-moiety C/D in UGT2B7*2 heterozygous (*2 He) carriers and *2 non carriers (*2 None).* Heterozygous carriers were defined as either 802CT or -161CT carriers, while *2 non-carriers were defined as either 802CC or -161CC carriers. Based on the data from k=5 trials and N=590 patients, there was no significant difference in carbamazepine active-moiety C/D between *2 heterozygous carriers and *2 non-carriers (fold-change: 0.95 [95%CI: 0.86, 1.05]; df=4; p=0.34). Low heterogeneity was observed (I^2=46%). *C/D: Plasma concentration-to-dose ratio.*

Study or Subgroup	log[Ratio of mean]	SE	UGT2B7 *2 Ho Total	UGT2B7 *2 None Total	Weight	Ratio of mean IV, Random, 95% CI
Zhang 2018	-0.23934779	0.19555388	8	40	12.4%	0.79 [0.54, 1.15]
Venkatraman 2023	0.08083061	0.1793561	19	49	14.3%	1.08 [0.76, 1.54]
Hung 2012	0.13605	0.10472787	17	133	30.9%	1.15 [0.93, 1.41]
Ma 2015	-0.08177279	0.07723088	19	68	42.5%	0.92 [0.79, 1.07]
**Total (95% CI)**			**63**	**290**	**100.0%**	**0.99 [0.85, 1.15]**

Heterogeneity: Tau² = 0.01; Chi² = 4.43, df = 3 (P = 0.22); I² = 32%
Test for overall effect: Z = 0.14 (P = 0.89)

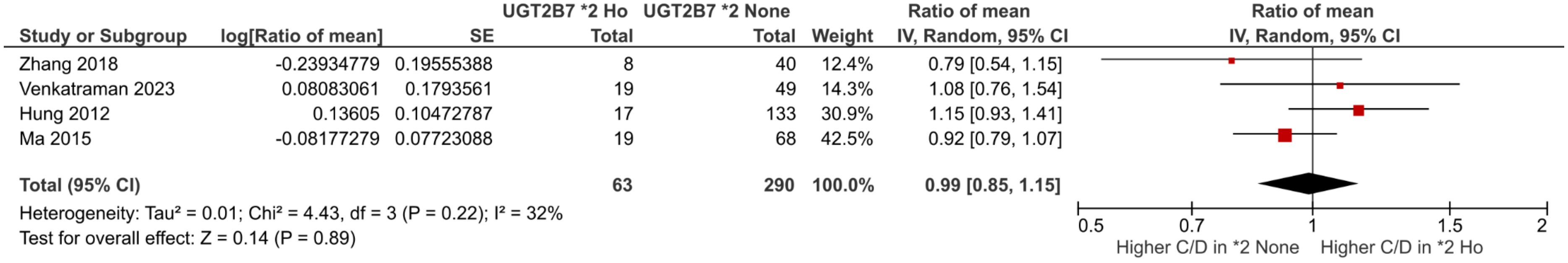

*Figure S26. Forest plot (RoM): Carbamazepine active-moiety C/D in UGT2B7*2 homozygous (*2 Ho) carriers and *2 non carriers (*2 None).* Homozygous carriers were defined as either 802TT or -161TT carriers, while *2 non-carriers were defined as either 802CC or -161CC carriers. Based on the data from k=4 trials and N=353 patients, there was no significant difference in carbamazepine active-moiety C/D between *2 homozygous carriers and *2 non-carriers (fold-change: 0.99 [95%CI: 0.85, 1.15]; df=3; p=0.89). Low heterogeneity was observed (I^2=32%). *C/D: Plasma concentration-to-dose ratio.*

© 2024 Milosavljević F et al. *JAMA Network Open.*

4) Funnel plots

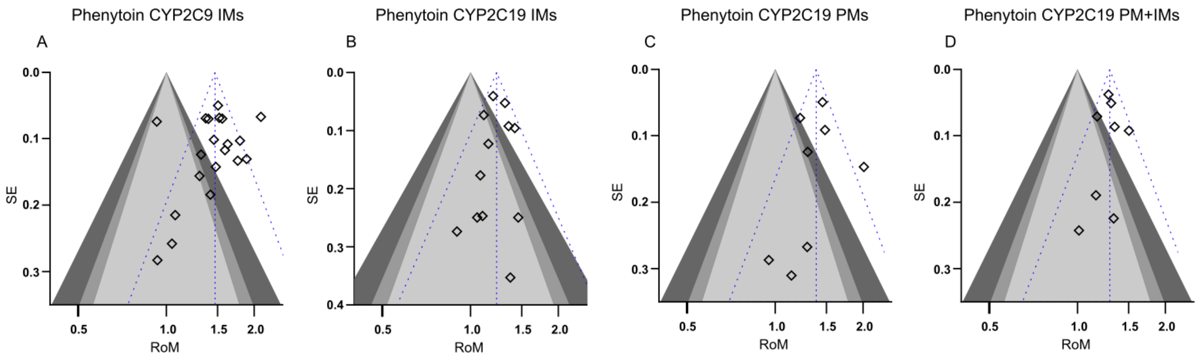

Figure S27. Funnel plot: Small-trial effect for Phenytoin meta-analyses: **A)** Asymmetry was observed both visually and by Egger test (p=0.031) for CYP2C9 IMs vs NMs meta-analysis. Small trials reporting more pronounced effects of CYP2C9 IM status are likely missing, indicating that the results reported here could be slightly underestimated. Regarding 3 meta-analyses on the impact of CYP2C19 gene on phenytoin C/D, no funnel plot asymmetry was observed both visually or by Egger test; p=0.52, p=0.49 and p=0.68 for CYP2C19 IMvsNM (C), CYP2C19 PMvsNMs (D) and CYP2C19 IM+PMvsNMs (E) respectively.

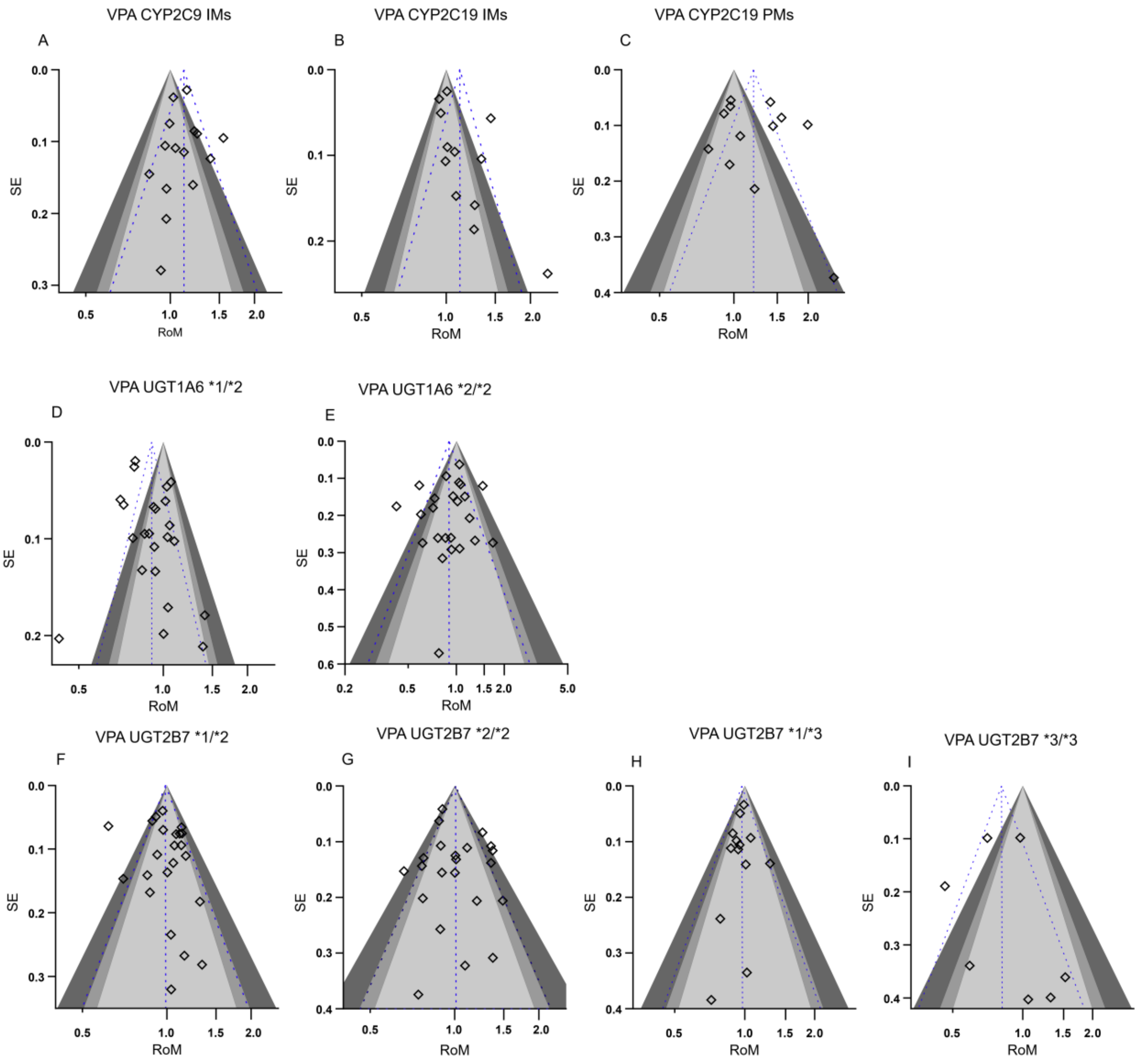

Figure S28. Funnel plot for Valproic Acid meta-analyses: No visual asymmetries were observed in any of the analyzed funnel plots. Egger test showed no significant funnel plot asymmetry in most of the meta-analyses: (A) p=0.61; (B) p=0.28; (C) p=0.27; (D) p=0.19; (F) p=0.88; (G) p=0.68; (H) p=0.48; (I) p=0.70; except for E) UGT1A6*2 homozygote meta-analysis where Egger's test showed significant effect of small trials (p=0.09).

© 2024 Milosavljević F et al. *JAMA Network Open.*

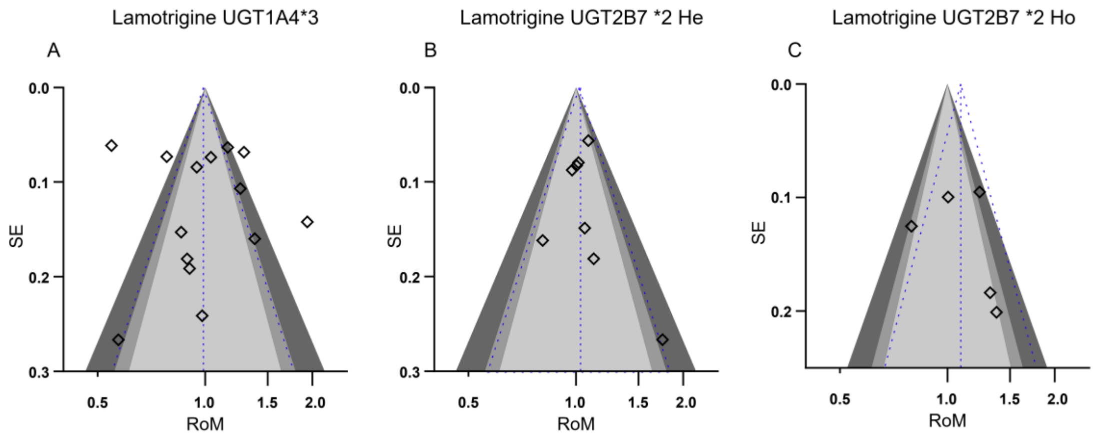

Figure S29. Funnel plot for Lamotrigine meta-analyses: No visual asymmetries were observed on funnel plots of all 3 meta-analyses. Egger's test didn't detect significant asymmetry (p=0.13) in the meta-analysis of Lamotrigine C/D in UGT1A4*3 carriers vs non-carriers (A), while two meta-analyses on UGT2B7 genotype (B, C) did not have the enough data to perform Egger's test.

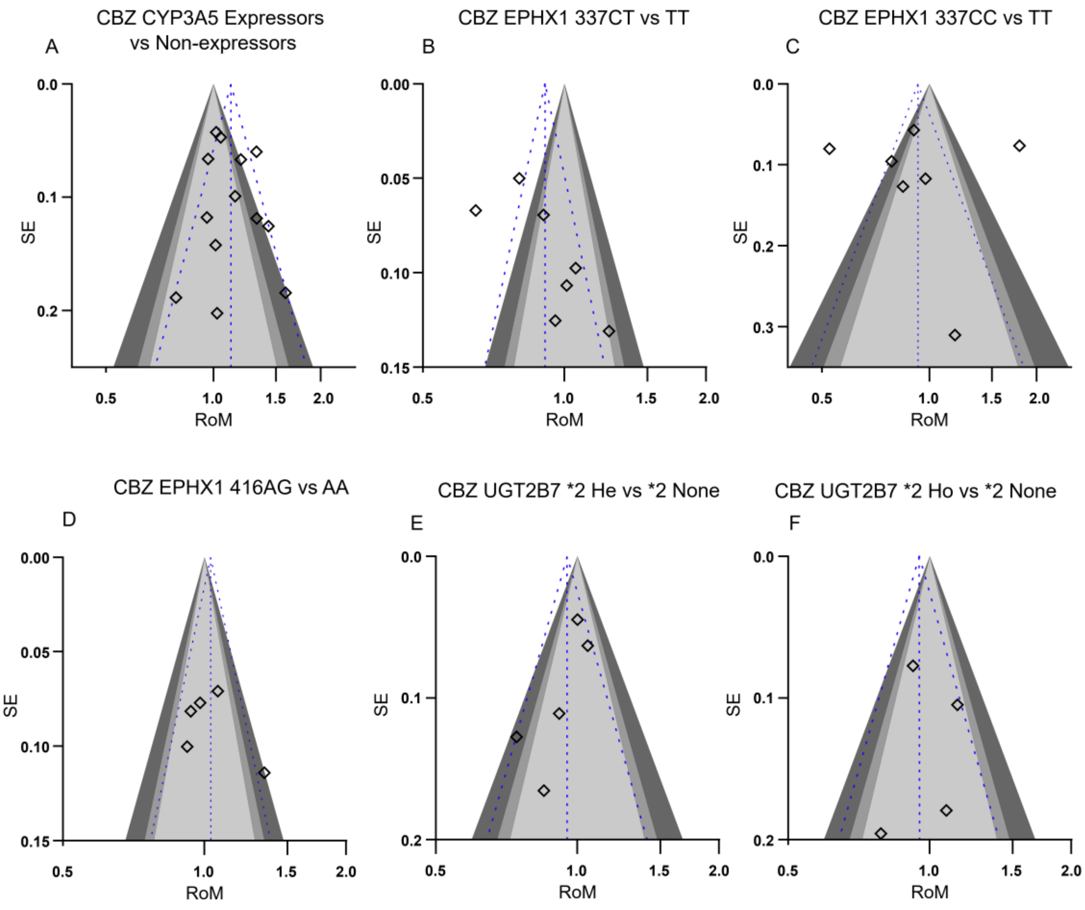

Figure S30. Funnel plot for Carbamazepine meta-analyses: No visual asymmetries were observed in any of the analyzed funnel plots. Egger test showed no significant funnel plot asymmetry (p=0.98) in the meta-analysis of carbamazepine active-moiety C/D in CYP3A5 expressors and non-expressors (A) while for the rest of the meta-analyses the number of included trials was insufficient for reliable funnel plot analysis (B-F).

© 2024 Milosavljević F et al. *JAMA Network Open.*

5) Risk-of-Bias analysis (ROBINS-I tool)

Bias was analyzed using ROBINS-I tool across seven bias domains: D1: Confounding; D2: Selection D3: Classification; D4: Deviations from intended intervention; D5: Missing data; D6: Outcome measurement; D7: Data reporting. Possible grades for each domain and overall risk, sorted from the lowest to the highest risk, were: L (Low risk), M (Moderate risk), S (Serious risk), C (Critical risk), NI (No information).

Table S10. RoB analysis: Phenytoin. Out of k=23 included trials across 4 meta-analyses related to Phenytoin: k=12 trials were assessed to have overall moderate RoB, k=9 trials were assessed to have overall serious RoB, and k=2 trials had insufficient data for reliable grading. No trial had low or critical overall risk of bias. Most common issues leading to the "Serious RoB" grade were insufficient control of confounding effects (such as CYP450, UGT1A6 or UGT2B7 inducing or inhibiting co-medications), and the risk of unbalanced times of blood sample collections for pharmacokinetic analysis.

Study	D1	D2	D3	D4	D5	D6	D7	Overall
Shaul 2022	L	L	L	L	L	M	M	**M**
Wanounou 2022	L	L	L	L	L	M	L	**M**
Fohner 2019	S	M	L	M	L	S	S	**S**
Guevara 2017	M	L	L	L	L	M	M	**M**
Li Z 2016	S	L	L	L	L	NI	S	**S**
Ortega-Vázquez 2015	M	L	L	L	L	M	M	**M**
Sharma 2015	M	M	L	L	L	M	S	**S**
Yamamoto 2015	M	L	L	L	L	M	M	**M**
George 2012	S	L	NI	L	L	M	M	**S**
Hung 2012	M	L	L	M	L	NI	M	**M**
Kesavan 2010	M	L	L	L	L	NI	M	**M**
Lin 2008	M	L	L	L	L	NI	M	**M**
Lee 2007	S	L	L	NI	L	M	M	**S**
Rosemary 2006	M	M	L	L	L	S	S	**S**
Yamanaka 2005	M	L	L	L	L	NI	M	**M**
Taguchi 2005	S	L	L	L	L	M	M	**S**
Huang 2004	M	NI	L	L	L	M	M	**M**
Hung 2004	S	M	L	NI	L	S	M	**S**
Soga 2004	NI	L	L	L	L	M	M	**NI**
Caraco 2001	L	L	L	L	L	M	M	**M**
van der Weide 2001	M	L	L	L	L	M	M	**M**
Aynacioglu 1999	NI	M	L	L	L	M	M	**NI**
Mamiya 1998	S	L	L	NI	L	NI	M	**S**

© 2024 Milosavljević F et al. *JAMA Network Open.*

Table S11. RoB analysis: Valproic acid. Out of k=45 included trials across 9 meta-analyses related to Valproic acid: k=23 trials were assessed to have overall moderate RoB, k=17 trials were assessed to have overall serious RoB, and k=5 trials had insufficient data for reliable grading. No trial had low or critical overall risk of bias. Most common issues leading to the “Serious RoB” grade were insufficient control of confounding effects (such as CYP450 inducing or inhibiting co-medications), and selective reporting of quantifiable data only for one subset of the cohort.

**Study**	**D1**	**D2**	**D3 CYP 2C9**	**D3 CYP 2C19**	**D3 UGT 1A6**	**D3 UGT 2B7**	**D4**	**D5**	**D6**	**D7**	**Overall**
Zheng 2023	M	L	L	N/A	M	M	L	L	M	M	M
Wang 2021	M	L	L	N/A	N/A	N/A	L	L	M	M	M
Du 2021	M	L	M	L	M	M	L	L	M	M	M
Wu 2021	S	M	N/A	L	N/A	N/A	L	L	S	S	S
Xu 2021	M	M	N/A	L	M	M	M	L	S	M	S
Nandith 2021	M	L	N/A	N/A	M	M	L	L	NI	M	M
Song 2020	M	L	L	M	N/A	N/A	L	L	M	M	M
Zhao 2020	M	L	N/A	N/A	M	M	L	L	M	M	M
Wang 2019	M	L	N/A	N/A	M	M	NI	L	NI	M	M
Feng 2019	M	L	N/A	L	M	M	L	L	M	M	M
Feng 2018	M	L	L	L	M	M	L	L	M	M	M
Zhang 2017	S	L	N/A	N/A	N/A	M	M	L	M	M	S
Sun 2017	M	L	L	M	N/A	M	L	L	M	M	M
Mei 2017	M	L	L	M	M	M	L	L	M	M	M
Li Z 2017	M	L	N/A	L	M	M	L	NI	M	M	M
Wang 2017	M	L	N/A	L	N/A	N/A	L	L	M	S	S
Zhao 2017	S	L	N/A	L	N/A	N/A	L	L	M	M	S
Jogamoto 2017	S	M	L	N/A	N/A	N/A	L	L	NI	M	S
Algharably 2016	M	L	N/A	N/A	M	N/A	L	L	M	M	M
Kang 2016	S	L	N/A	N/A	M	N/A	L	L	S	M	S
Feng 2016	M	M	L	N/A	M	M	L	L	S	M	S
Du 2016	M	L	N/A	N/A	N/A	M	L	L	M	M	M
Chatzistefanidis 2016	M	L	N/A	N/A	M	M	L	L	M	M	M
Shen 2016	M	L	N/A	N/A	M	M	NI	L	NI	M	M
Wang 2016	M	L	N/A	N/A	N/A	M	L	L	M	M	M
Wen 2016	S	M	N/A	N/A	N/A	M	L	L	S	L	S
Smith 2016	S	M	L	L	N/A	N/A	L	L	S	M	S
Sun 2015	NI	L	N/A	N/A	N/A	M	L	L	M	M	NI
Han 2015	NI	NI	L	N/A	N/A	N/A	NI	NI	NI	NI	NI
Tóth 2015	M	L	N/A	L	N/A	N/A	L	L	M	M	M
Jain 2015	M	M	N/A	N/A	M	N/A	L	L	M	M	M
Aphichartphunkawee 2014	S	L	N/A	N/A	M	N/A	L	L	M	M	S
Inoue 2014	S	L	N/A	N/A	N/A	M	L	L	M	M	S
Liao 2013	M	L	L	L	N/A	N/A	L	L	M	M	M
Ma 2013	S	L	N/A	N/A	N/A	M	L	L	NI	M	S
Rutigliano 2013	NI	NI	L	N/A	N/A	N/A	NI	NI	M	NI	NI
Jin 2013	S	L	N/A	N/A	M	N/A	L	L	NI	M	S
Guo 2012	M	L	N/A	L	N/A	M	L	NI	M	M	M

© 2024 Milosavljević F et al. *JAMA Network Open.*

Table S11. Continued

Study	D1	D2	D3 CYP 2C9	D3 CYP 2C19	D3 UGT 1A6	D3 UGT 2B7	D4	D5	D6	D7	Overall
Chu 2012	M	L	N/A	N/A	M	M	L	L	M	M	M
Sterjev 2012	NI	NI	N/A	N/A	M	N/A	NI	NI	NI	NI	NI
Hung 2011	S	L	N/A	N/A	M	M	NI	L	NI	M	S
Wang 2010	NI	L	N/A	N/A	M	N/A	L	L	NI	M	NI
Tan 2010	S	L	N/A	L	N/A	N/A	L	L	M	M	S
Chung 2008	M	M	N/A	N/A	N/A	M	L	L	M	M	M
Sun 2007	S	L	N/A	N/A	M	N/A	L	L	NI	M	S

Table S12. RoB analysis: Lamotrigine. Out of k=14 included trials across 3 meta-analyses related to Lamotrigine: k=8 trials were assessed to have overall moderate RoB and k=6 trials were assessed to have overall serious RoB. No trial had low or critical overall risk of bias. Most common issue leading to the "Serious RoB" grade was insufficient control of confounding effects such as valproic acid and phenytoin which are known to greatly influence lamotrigine C/D.

Study	D1	D2	D3	D4	D5	D6	D7	Overall
**Božina 2023**	L	L	M	L	L	M	M	**M**
**Petrenaite 2022**	S	L	M	M	L	M	M	**S**
**Ortega-Vázquez 2020**	S	L	M	L	L	M	M	**S**
**Suzuki 2019**	S	L	M	L	L	M	M	**S**
**Petrenaite 2018**	S	M	M	M	L	S	M	**S**
**Smith 2018**	S	M	M	L	L	S	M	**S**
**He 2017**	M	L	M	L	L	M	M	**M**
**Du 2016**	M	L	M	L	L	M	M	**M**
**Reimers 2016**	S	L	M	L	L	S	M	**S**
**Wang 2016**	M	L	M	L	L	M	M	**M**
**Liu 2015**	M	L	M	L	L	M	M	**M**
**Wang 2015**	M	L	M	L	L	M	M	**M**
**Zhou 2015**	M	L	M	L	L	M	M	**M**
**Chang 2014**	M	L	M	L	L	M	M	**M**

© 2024 Milosavljević F et al. *JAMA Network Open.*

Table S13. RoB analysis: Carbamazepine. Out of k=19 included trials across 6 meta-analyses related to Carbamazepine: k=4 trials were assessed to have overall moderate RoB, k=13 trials were assessed to have overall serious RoB, and k=2 trials had insufficient data for reliable grading. No trial had low or critical overall risk of bias. Most common issues leading to the “Serious RoB” grade were insufficient control of confounding effects (such as CYP450 or UGT inducing or inhibiting co-medications) and missing data on active metabolite. Also, many trials reported the risk of unbalanced times of blood sample collections for pharmacokinetic analysis between groups.

Study	D1	D2	D3	D4	D5	D6	D7	Overall
Venkatraman 2023	M	L	M	NI	L	NI	M	M
Pham 2020	S	L	M	L	L	S	M	S
Ganesapandian 2019	S	L	M	NI	L	NI	M	S
Lu 2018	S	L	M	L	L	M	L	S
Liu 2016	S	L	M	NI	NI	NI	NI	NI
Zhang 2018	S	L	M	L	L	M	M	S
Chbili 2016	M	L	M	NI	L	NI	M	M
Daci 2015	M	L	M	L	L	M	M	M
Wang 2015	M	L	M	L	L	M	S	S
Liu 2014	S	L	NI	NI	NI	NI	NI	NI
Ma 2015	M	L	M	L	L	M	M	M
Zhu 2014	S	L	M	L	L	M	M	S
Saruwatari 2014	S	M	M	L	L	S	S	S
Panomvana 2013	S	M	M	L	L	S	M	S
Yun 2013	S	L	M	L	L	M	M	S
Hung 2012	S	L	M	NI	L	NI	M	S
Meng 2011	S	L	M	L	L	S	M	S
Park 2009	S	L	M	L	L	M	M	S
Seo 2006	S	L	M	M	L	S	M	S

© 2024 Milosavljević F et al. *JAMA Network Open.*

6) Sensitivity analysis

6.1) Sensitivity analyses: Phenytoin (Tables S14-16)

Table S14. Sensitivity analysis: Phenytoin C/D in CYP2C9 IMvsNM. Sensitivity analysis demonstrated that reported meta-analysis results did not significantly change: 1) in both healthy volunteers taking single dose of phenytoin and neurology patients (epilepsy, post-operation prophylaxis…) taking multiple doses and achieving steady state; 2) in cohorts from different ethnic backgrounds; 3) in trials with different RoB grades; 4) if the most positive or negative trials were excluded; 4) if only large (total number of subjects is more than 100) cohorts were included or 5) in only trials on adults were included. This indicates very high level of robustness of the original meta-analysis results. *RoB: Risk-of-bias; N/A: Not applicable.*

[a] : p-value is produced by comparing the given model to the base model.

Model	Trials	Sample	Effect	p	I^2	Sensitivity (p)
Base model (most inclusive)	k=20	N=2272	1.46 [1.33, 1.61]	<0.001	79%	N/A
Adult cohorts (≥18 years old)	k=14	N=1821	1.43 [1.27, 1.61]	<0.001	83%	p=0.78[a]
Large trials (n>100)	k=9	N=1723	1.53 [1.38, 1.69]	<0.001	74%	p=0.52[a]
Most strict model (Moderate RoB, large trial and only adult cohorts)	k=7	N=1235	1.53 [1.35, 1.74]	<0.001	80%	p=0.58[a]
Most positive trial excluded	k=19	N=1988	1.43 [1.31, 1.56]	<0.001	69%	p=0.73[a]
Most negative trial excluded	k=19	N=2225	1.52 [1.42, 1.64]	<0.001	58%	p=0.49[a]
Trials with Moderate or NI RoB grade	k=12	N=1384	1.52 [1.38, 1.67]	<0.001	68%	p=0.44
Trials with Serious RoB grade	k=8	N=888	1.39 [1.13, 1.70]	<0.001	85%	
East Asian cohorts	k=8	N=980	1.52 [1.39, 1.66]	<0.001	18%	p=0.82
(Predominantly) Caucasian cohorts	k=6	N=693	1.47 [1.38, 1.56]	<0.001	0%	
South Asian cohorts	k=4	N=392	1.42 [0.87, 2.33]	0.17	80%	
Neurology patient cohorts	k=15	N=1837	1.46 [1.28, 1.67]	<0.001	83%	p=0.93
Healthy volunteer cohorts	k=5	N=435	1.47 [1.38, 1.57]	<0.001	0%	

© 2024 Milosavljević F et al. *JAMA Network Open.*

Table S15. Sensitivity analysis: Phenytoin C/D in CYP2C19 IMvsNM. Sensitivity analysis demonstrated that reported meta-analysis results did not significantly change: 1) in trials with different RoB grades; 2) if the most positive or negative trials were excluded; 3) if only large (total number of subjects is more than 100) cohorts were included or 4) in only trials on adults were included. Since 9 out of 12 included trials were conducted on East-Asian cohorts, and since no included trial was conducted on healthy volunteers; there was not enough data for certain sensitivity analyses. It is hard to ascertain high level of certainty regarding the result robustness as the data is limited, but for the cases for which the data is available, results are highly robust. *RoB: Risk-of-bias; N/A: Not applicable.*

[a] : p-value is produced by comparing the given model to the base model.

Model	Trials	Sample	Effect	p	I^2	Sensitivity (p)
Base model (most inclusive)	k=12	N=1115	1.23 [1.17, 1.30]	<0.001	0%	N/A
Adult cohorts (≥18 years old)	k=7	N=827	1.24 [1.15, 1.32]	<0.001	0%	p=0.95[a]
Large trials (n>100)	k=5	N=858	1.23 [1.15, 1.32]	<0.001	23%	p=0.97[a]
Most strict model (Moderate RoB, large trial and only adult cohorts)	k=2	N=485	1.30 [1.18, 1.44]	<0.001	0%	p=0.32[a]
Most positive trial excluded	k=11	N=1101	1.23 [1.17, 1.29]	<0.001	1%	p=0.96[a]
Most negative trial excluded	k=11	N=1056	1.24 [1.18, 1.30]	<0.001	0%	p=0.94[a]
Trials with Moderate or NI RoB grade	k=8	N=770	1.22 [1.15, 1.30]	<0.001	0%	p=0.51
Trials with Serious RoB grade	k=4	N=345	1.29 [1.12, 1.48]	<0.001	45%	
East Asian cohorts	k=9	N=773	1.24 [1.18, 1.31]	<0.001	5%	p=0.67
(Predominantly) Caucasian cohorts	k=1	N=50	1.08 [0.76, 1.53]	0.66	N/A	
South Asian cohorts	k=1	N=233	1.10 [0.68, 1.79]	0.70	N/A	
Healthy volunteer cohorts	k=0	N=0	N/A	N/A	N/A	N/A
Neurology patient cohorts	k=12	N=1115	1.23 [1.17, 1.30]	<0.001	0%	

Table S16. Sensitivity analysis: Phenytoin C/D in CYP2C19 PMvsNM. The only sensitivity analysis with sufficient data demonstrated that results did not significantly change if the most positive or negative trials were excluded. It is hard to ascertain high level of certainty regarding the result robustness as the data is limited for the majority of the sensitivity analyses. *RoB: Risk-of-bias; N/A: Not applicable.*

[a] : p-value is produced by comparing the given model to the base model.

Model	Trials	Sample	Effect	p	I^2	Sensitivity (p)
Base model (most inclusive)	k=8	N=557	1.39 [1.24, 1.56]	<0.001	48%	N/A
Adult cohorts (≥18 years old)	k=4	N=409	1.31 [1.19, 1.45]	<0.001	0%	p=0.44[a]
Large trials (n>100)	k=2	N=279	1.22 [1.07, 1.40]	0.004	0%	p=0.16[a]
Most strict model (Moderate RoB, large trial and only adult cohorts)	k=2	N=279	1.22 [1.07, 1.40]	0.004	0%	p=0.16[a]
Most positive trial excluded	k=7	N=514	1.36 [1.25, 1.47]	<0.001	14%	p=0.73[a]
Most negative trial excluded	k=7	N=545	1.40 [1.30, 1.51]	<0.001	44%	p=0.86[a]
Trials with Moderate or NI RoB grade	k=3	N=361	1.34 [1.17, 1.54]	<0.001	51%	p=0.04
Trials with Serious RoB grade	k=4	N=188	1.50 [1.23, 1.84]	<0.001	52%	
East Asian cohorts	k=7	N=395	1.40 [1.24, 1.58]	<0.001	55%	p=0.55
(Predominantly) Caucasian cohorts	k=0	N=0	N/A	N/A	N/A	
South Asian cohorts	k=1	N=162	1.29 [0.76, 2.17]	0.34	N/A	
Neurology patient cohorts	k=8	N=557	1.39 [1.24, 1.56]	<0.001	48%	N/A
Healthy volunteer cohorts	k=0	N=0	N/A	N/A	N/A	

6.2) Sensitivity analyses: Valproic acid (Tables S17-24)

Table S17. Sensitivity analysis: Valproic acid C/D in CYP2C9 IMvsNM. Sensitivity analysis demonstrated that reported meta-analysis results did not significantly change: 1) In both adult and pediatric cohorts; 2) in trials with different RoB grades; 3) if the most positive or negative trials were excluded; or 4) if only large cohorts (total number of subjects is more than 100) were included. Since no trials on healthy volunteers were included, and since great majority of trials were conducted on East-Asian cohorts; there was no enough data for all planned sensitivity analyses. It is hard to ascertain high level of certainty regarding the result robustness as the data is limited, but for the cases for which the data is available, results are highly robust *RoB: Risk-of-bias; N/A: Not applicable.*

[a] : p-value is produced by comparing the given model to the base model.

Model	Trials	Sample	Effect	p	I^2	Sensitivity (p)
Base model (most inclusive)	k=15	N=2287	1.12 [1.04, 1.20]	0.003	59%	N/A
Large trials (n>100)	k=10	N=1951	1.12 [1.03, 1.22]	0.009	67%	p=0.96[a]
Most strict model (Moderate RoB, large trial and only adult cohorts)	k=4	N=923	1.14 [0.92, 1.41]	0.22	86%	p=0.84[a]
Most positive trial excluded	k=14	N=2156	1.09 [1.03, 1.16]	0.005	39%	p=0.62[a]
Most negative trial excluded	k=14	N=2204	1.13 [1.05, 1.21]	0.001	59%	p=0.84[a]
Adult cohorts	k=6	N=1005	1.13 [0.98, 1.30]	0.09	78%	p=0.85
Pediatric cohorts	k=9	N=1234	1.15 [1.01, 1.30]	0.03	54%	
Trials with Moderate or NI RoB grade	k=8	N=1190	1.14 [0.99, 1.31]	0.07	74%	p=0.82
Trials with Serious RoB grade	k=6	N=1028	1.12 [1.05, 1.19]	<0.001	15%	
East Asian cohort	k=12	N=1919	1.13 [1.04, 1.23]	0.004	64%	p=0.21
(Predominantly) Caucasian cohorts	k=3	N=368	1.03 [0.92, 1.16]	0.56	0%	
Healthy volunteer cohorts	k=0	N=0	N/A	N/A	N/A	N/A
Neurology patient cohorts	k=15	N=2287	1.12 [1.04, 1.20]	0.003	59%	

© 2024 Milosavljević F et al. *JAMA Network Open.*

Table S18. Sensitivity analysis: Valproic acid C/D in CYP2C19 IMvsNM. Sensitivity analysis demonstrated that reported meta-analysis results did not significantly change only if the most negative trial was excluded. Inclusion only of large trials (total number of subjects is more than 100) or only trials on serious RoB caused the loss of the statistical significance indicating poor robustness of available data on the influence of CYP2C19 gene on Valproic acid C/D. Also, sensitivity analysis showed complete lack of effect in trials that predominantly included children, while trials on adults had 2 times more pronounced effect than original meta-analysis. The effect observed in adults was not statistically significant, likely due to the low number of participants. There was no enough data to analyze the effect of ethnicity and the differences between healthy volunteer vs neurology patient data. *RoB: Risk-of-bias; N/A: Not applicable.*

[a] : p-value is produced by comparing the given model to the base model.

Model	Trials	Sample	Effect	p	I^2	Sensitivity (p)
Base model (most inclusive)	k=12	N=1594	1.12 [1.02, 1.24]	0.02	83%	N/A
Large trials (n>100)	k=7	N=1376	1.05 [0.95, 1.17]	0.35	87%	p=0.37[a]
Most strict model (Moderate RoB, large trial and only adult cohorts)	k=5	N=1057	1.07 [0.90, 1.27]	0.42	91%	p=0.64[a]
Most positive trial excluded	k=11	N=1553	1.09 [1.00, 1.20]	0.06	82%	p=0.70[a]
Most negative trial excluded	k=11	N=1244	1.16 [1.03, 1.30]	0.01	82%	p=0.72[a]
Adult cohorts	k=5	N=886	1.27 [0.99, 1.64]	0.07	93%	p=0.08
Pediatric cohorts	k=6	N=684	1.01 [0.97, 1.05]	0.67	0%	
Trials with Moderate or NI RoB grade	k=8	N=1205	1.18 [1.01, 1.37]	0.04	89%	p=0.42
Trials with Serious RoB grade	k=3	N=240	1.08 [0.93, 1.25]	0.33	0%	
East Asian cohort	k=11	N=1424	1.14 [1.02, 1.26]	0.02	85%	p=0.26
(Predominantly) Caucasian cohorts	k=1	N=170	1.00 [0.81, 1.23]	0.97	N/A	
Neurology patient cohorts	k=12	N=1594	1.12 [1.02, 1.24]	0.02	83%	N/A
Healthy volunteer cohorts	k=0	N=0	N/A	N/A	N/A	

Table S19. Sensitivity analysis: Valproic acid C/D in CYP2C19 PMvsNM. Sensitivity analysis demonstrated that reported meta-analysis results did not significantly change only if the most negative trial was excluded. Inclusion only of large trials (total number of subjects is more than 100) or only trials on serious RoB caused the loss of the statistical significance indicating poor robustness of available data on the influence of CYP2C19 gene on Valproic acid C/D. Also, sensitivity analysis showed complete lack of effect in trials that predominantly included children, while trials on adults had more pronounced effect than original meta-analysis. The effect observed in adults was not statistically significant, likely due to the low number of participants in the available trails. There was no enough data to analyze the effect of ethnicity and the differences between healthy volunteer vs neurology patient data. *RoB: Risk-of-bias; N/A: Not applicable.*

[a] : p-value is produced by comparing the given model to the base model.

Model	Trials	Sample	Effect	p	I^2	Sensitivity (p)
Base model (most inclusive)	k=12	N=1004	1.20 [1.02, 1.41]	0.03	89%	N/A
Large trials (n>100)	k=5	N=714	1.13 [0.92, 1.39]	0.23	92%	p=0.66[a]
Most strict model (Moderate RoB, large trial and only adult cohorts)	k=4	N=559	1.30 [0.94, 1.79]	0.11	95%	p=0.67[a]
Most positive trial excluded	k=11	N=976	1.17 [0.99, 1.38]	0.06	89%	p=0.83[a]
Most negative trial excluded	k=11	N=991	1.24 [1.05, 1.47]	0.01	89%	p=0.76[a]
Adult cohorts	k=5	N=570	1.38 [0.99, 1.92]	0.06	94%	p=0.26
Pediatric cohorts	k=5	N=321	1.11 [0.93, 1.33]	0.25	66%	
Trials with Moderate or NI RoB grade	k=8	N=758	1.32 [1.06, 1.64]	0.01	90%	p=0.002
Trials with Serious RoB grade	k=3	N=146	0.89 [0.79, 1.01]	0.07	0%	
East Asian cohort	k=11	N=899	1.24 [1.04, 1.47]	0.02	89%	p=0.010
(Predominantly) Caucasian cohorts	k=1	N=105	0.91 [0.78, 1.06]	0.24	N/A	
Healthy volunteer cohorts	k=0	N=0	N/A	N/A	N/A	N/A
Neurology patient cohorts	k=12	N=1004	1.20 [1.02, 1.41]	0.03	89%	

© 2024 Milosavljević F et al. *JAMA Network Open.*

*Table S20. Sensitivity analysis: Valproic acid C/D in UGT1A6 *2 Heterozygous carriers and non-carriers.* Sensitivity analysis demonstrated that reported meta-analysis results did not significantly change: 1) if the most negative trial was excluded or 2) only large trials (total number of subjects is more than 100) were included. There was complete lack of effect in trials that predominantly included children, while trials on adults had slightly more pronounced effect than original meta-analysis. The effect observed in adults was not statistically significant, likely due to the low number of participants in the available trails. Similar effect was observed in sensitivity analysis of trials with different RoB grade where trials with worse RoB grade had much more pronounced positive results that trials with better RoB grade. There was no enough data to analyze the effect of ethnicity and the differences between healthy volunteer vs neurology patient data. *RoB: Risk-of-bias; N/A: Not applicable.*

[a] : p-value is produced by comparing the given model to the base model.

Model	Trials	Sample	Effect	p	I^2	Sensitivity (p)
Base model (most inclusive)	k=25	N=2839	0.91 [0.85, 0.97]	0.004	84%	N/A
Large trials (n>100)	k=13	N=2063	0.93 [0.87, 1.01]	0.08	74%	p=0.59[a]
Most strict model (Moderate RoB, large trial and only adult cohorts)	k=6	N=893	1.01 [0.96, 1.07]	0.63	0%	p=0.01[a]
Most positive trial excluded	k=24	N=2796	0.92 [0.86, 0.98]	0.01	83%	p=0.77[a]
Most negative trial excluded	k=24	N=2772	0.90 [0.84, 0.96]	0.001	83%	p=0.83[a]
Adult cohorts	k=8	N=1004	0.85 [0.74, 0.98]	0.02	90%	p=0.07
Pediatric cohorts	k=14	N=1428	0.98 [0.92, 1.05]	0.59	30%	
Trials with Moderate or NI RoB grade	k=15	N=1466	0.98 [0.90, 1.06]	0.004	56%	p=0.008
Trials with Serious RoB grade	k=8	N=1102	0.82 [0.75, 0.90]	<0.001	88%	
East Asian cohort	k=19	N=2312	0.90 [0.83, 0.98]	0.01	85%	p=0.02
(Predominantly) Caucasian cohorts	k=2	N=247	0.96 [0.86, 1.07]	0.44	0%	
South Asian cohorts	k=2	N=159	1.07 [0.65, 1.78]	0.78	82%	
Thai Cohort	k=1	N=78	0.79 [0.75, 0.83]	<0.001	N/A	
Egyptian Cohort	k=1	N=41	0.93 [0.75, 1.15]	0.50	N/A	
Healthy volunteer cohorts	k=0	N=0	N/A	N/A	N/A	N/A
Neurology patient cohorts	k=25	N=2839	0.91 [0.85, 0.97]	0.004	84%	

© 2024 Milosavljević F et al. *JAMA Network Open.*

*Table S21. Sensitivity analysis: Valproic acid C/D in UGT1A6 *2 Homozygous carriers and non-carriers.* Sensitivity analysis demonstrated that reported meta-analysis results did not significantly change: 1) if the most negative trial was excluded; 2) only large trials (total number of subjects is more than 100) were included; 3) in both adult and pediatric cohorts; 4) trials with different RoB grades. This indicates good robustness of the observed effect. There was no enough data to analyze the effect of ethnicity and the differences between healthy volunteer vs neurology patient data. *RoB: Risk-of-bias; N/A: Not applicable.*

[a] : p-value is produced by comparing the given model to the base model.

Model	Trials	Sample	Effect	p	I^2	Sensitivity (p)
Base model (most inclusive)	k=24	N=1790	0.90 [0.80, 1.02]	0.11	71%	N/A
Large trials (n>100)	k=6	N=822	0.93 [0.73, 1.19]	0.58	77%	p=0.83[a]
Most strict model (Moderate RoB, large trial and only adult cohorts)	k=3	N=348	1.07 [0.88, 1.29]	0.51	0%	p=0.15[a]
Most positive trial excluded	k=23	N=1763	0.94 [0.84, 1.05]	0.27	62%	p=0.66[a]
Most negative trial excluded	k=23	N=1767	0.89 [0.78, 1.00]	0.06	71%	p=0.83[a]
Adult cohorts	k=8	N=671	0.87 [0.65, 1.16]	0.35	88%	p=0.57
Pediatric cohorts	k=14	N=916	0.96 [0.85, 1.10]	0.57	26%	
Trials with Moderate or NI RoB grade	k=15	N=932	0.88 [0.73, 1.06]	0.18	67%	p=0.69
Trials with Serious RoB grade	k=7	N=684	0.94 [0.73, 1.20]	0.61	83%	
East Asian cohort	k=18	N=1451	0.89 [0.77, 1.03]	0.11	71%	p=0.001
(Predominantly) Caucasian cohorts	k=2	N=160	0.97 [0.75, 1.25]	0.80	57%	
South Asian cohorts	k=2	N=87	0.84 [0.57, 1.25]	0.39	0%	
Thai Cohort	k=1	N=60	1.47 [1.16, 1.85]	0.0001	N/A	
Egyptian Cohort	k=1	N=32	0.60 [0.41, 0.88]	0.010	N/A	
Healthy volunteer cohorts	k=0	N=0	N/A	N/A	N/A	N/A
Neurology patient cohorts	k=24	N=1790	0.90 [0.80, 1.02]	0.11	71%	

© 2024 Milosavljević F et al. *JAMA Network Open.*

*Table S22. Sensitivity analysis: Valproic acid C/D in UGT2B7*3 Heterozygous carriers and non-carriers.* Sensitivity analysis demonstrated that reported meta-analysis results did not significantly change: 1) if the most negative trial was excluded; 2) only large trials (total number of subjects is more than 100) were included; 3) in both adult and pediatric cohorts; 4) trials with different RoB grades. This indicates good robustness of the observed effect. There was no enough data to analyze the effect of ethnicity and the differences between healthy volunteer vs neurology patient data. There were also not enough included trials to perform the sensitivity analysis on meta-analysis of Valproic acid C/D in UGT2B7 *3 homozygous carriers and *3 non-carriers. *RoB: Risk-of-bias; N/A: Not applicable.*

[a] : p-value is produced by comparing the given model to the base model.

Model	Trials	Sample	Effect	p	I^2	Sensitivity (p)
Base model (most inclusive)	k=13	N=1866	0.97 [0.93, 1.01]	0.19	0%	N/A
Large trials (n>100)	k=8	N=1559	0.97 [0.93, 1.01]	0.16	0%	p=0.91[a]
Most strict model (Moderate RoB, large trial and only adult cohorts)	k=5	N=1035	0.97 [0.93, 1.02]	0.26	0%	p=0.97[a]
Most positive trial excluded	k=12	N=1840	0.97 [0.93, 1.02]	0.21	0%	p=0.97[a]
Most negative trial excluded	k=12	N=1769	0.96 [0.92, 1.01]	0.11	0%	p=0.82[a]
Adult cohorts	k=4	N=616	0.99 [0.93, 1.05]	0.69	0%	p=0.54
Pediatric cohorts	k=8	N=1013	0.96 [0.89, 1.04]	0.28	0%	
Trials with Moderate or NI RoB grade	k=10	N=1342	0.98 [0.93, 1.02]	0.31	0%	p=0.40
Trials with Serious RoB grade	k=2	N=423	0.91 [0.79, 1.06]	0.24	0%	
East Asian cohort	k=12	N=1769	0.96 [0.92, 1.01]	0.92	0%	p=0.04
(Predominantly) Caucasian cohorts	k=0	N=0	N/A	N/A	N/A	
South Asian cohorts	k=1	N=97	1.29 [0.98, 1.69]	0.07	N/A	
Healthy volunteer cohorts	k=0	N=0	N/A	N/A	N/A	N/A
Neurology patient cohorts	k=13	N=1866	0.97 [0.93, 1.01]	0.19	0%	

*Table S23. Sensitivity analysis: Valproic acid C/D in UGT2B7*2 Heterozygous carriers and non-carriers.* Sensitivity analysis demonstrated that reported meta-analysis results did not significantly change: 1) if the most negative trial was excluded; 2) only large trials (total number of subjects is more than 100) were included; 3) in both adult and pediatric cohorts; 4) trials with different RoB grades. This indicates good robustness of the observed effect. There was no enough data to analyze the effect of ethnicity and the differences between healthy volunteer vs neurology patient data. *RoB: Risk-of-bias; N/A: Not applicable.*

[a] : p-value is produced by comparing the given model to the base model.

Model	Trials	Sample	Effect	p	I^2	Sensitivity (p)
Base model (most inclusive)	k=23	N=2489	0.99 [0.91, 1.06]	0.72	75%	N/A
Large trials (n>100)	k=11	N=1832	1.01 [0.95, 1.07]	0.81	49%	p=0.67[a]
Most strict model (Moderate RoB, large trial and only adult cohorts)	k=7	N=1264	0.99 [0.92, 1.06]	0.76	50%	p=0.96[a]
Most positive trial excluded	k=22	N=2418	0.98 [0.91, 1.06]	0.64	75%	p=0.93[a]
Most negative trial excluded	k=22	N=2428	1.01 [0.96, 1.06]	0.67	39%	p=0.59[a]
Adult cohorts	k=8	N=875	0.97 [0.88, 1.06]	0.50	48%	p=0.67
Pediatric cohorts	k=15	N=1507	1.00 [0.89, 1.12]	0.99	81%	
Trials with Moderate or NI RoB grade	k=16	N=1740	0.99 [0.89, 1.09]	0.80	78%	p=0.44
Trials with Serious RoB grade	k=6	N=689	1.04 [0.95, 1.14]	0.37	34%	
East Asian cohort	k=21	N=2275	0.98 [0.90, 1.06]	0.60	76%	p=0.38
(Predominantly) Caucasian cohorts	k=1	N=134	1.13 [0.94, 1.35]	0.20	N/A	
South Asian cohorts	k=1	N=80	1.01 [0.77, 1.31]	0.97	N/A	
Healthy volunteer cohorts	k=1	N=10	1.31 [0.92, 1.88]	0.13	N/A	p=0.11
Neurology patient cohorts	k=22	N=2479	0.98 [0.91, 1.06]	0.57	75%	

*Table S24. Sensitivity analysis: Valproic acid C/D in UGT2B7 *2 Homozygous carriers and non-carriers.* Sensitivity analysis demonstrated that reported meta-analysis results did not significantly change: 1) if the most negative trial was excluded; 2) only large trials (n>100) were included; 3) in both adult and pediatric cohorts; 4) trials with different RoB grades. This indicates good robustness of the observed effect. There was no enough data to analyze the effect of ethnicity and the differences between healthy volunteer vs neurology patient data. *RoB: Risk-of-bias; N/A: Not applicable.*

[a] : p-value is produced by comparing the given model to the base model.

Model	Trials	Sample	Effect	p	I^2	Sensitivity (p)
Base model (most inclusive)	k=22	N=1507	1.01 [0.92, 1.11]	0.84	66%	N/A
Large trials (n>100)	k=5	N=678	1.05 [0.88, 1.26]	0.59	73%	p=0.70[a]
Most strict model (Moderate RoB, large trial and only adult cohorts)	k=3	N=469	0.94 [0.82, 1.07]	0.35	42%	p=0.38[a]
Most positive trial excluded	k=21	N=1498	1.00 [0.91, 1.09]	0.95	65%	p=0.85[a]
Most negative trial excluded	k=21	N=1437	1.03 [0.94, 1.13]	0.53	64%	p=0.77[a]
Adult cohorts	k=7	N=528	1.02 [0.84, 1.24]	0.81	69%	p=0.93
Pediatric cohorts	k=14	N=909	1.03 [0.93, 1.15]	0.55	63%	
Trials with Moderate or NI RoB grade	k=15	N=1017	1.03 [0.94, 1.13]	0.52	57%	p=0.73
Trials with Serious RoB grade	k=6	N=434	0.98 [0.75, 1.29]	0.89	81%	
East Asian cohort	k=21	N=1450	1.01 [0.92, 1.11]	0.85	68%	p=0.98
(Predominantly) Caucasian cohorts	k=0	N=0	N/A	N/A	N/A	
South Asian cohorts	k=1	N=57	1.01 [0.78, 1.31]	0.92	N/A	
Healthy volunteer cohorts	k=1	N=9	1.49 [0.99, 2.22]	0.05	N/A	p=0.06
Neurology patient cohorts	k=21	N=1498	1.00 [0.91, 1.09]	0.95	65%	

© 2024 Milosavljević F et al. *JAMA Network Open.*

6.3) Sensitivity analyses: Lamotrigine (Tables S25-26)

*Table S25. Sensitivity analysis: Lamotrigine C/D in UGT1A4*3 carriers and non-carriers.* Sensitivity analysis demonstrated that reported meta-analysis results did not significantly change: 1) In both adult and pediatric cohorts; 2) if the most positive or negative trials were excluded; or 3) if only large cohorts (total number of subjects is more than 100) were included. Since no trials on healthy volunteers were included, there was no enough data for all planned sensitivity analyses. Trials with moderate RoB grade reported slight increase in lamotrigine C/D in UGT1A4*3 carriers while trials with serious RoB grade reported slight decrease in C/D in UGT1A4*3 carriers compared to non-carriers. In both subgroups, effects were not statistically significant. Similar effect was observed in the sensitivity analysis of cohort ethnicity: trials on East Asian cohort reported slight increase while trials on Caucasian cohort reported slight decrease in lamotrigine C/D in UGT1A4*3 carriers compared to non-carriers. This indicates poor robustness of the observed results where cohort ethnicity and/or RoB grade are observed to be significant confounding factors. *RoB: Risk-of-bias; N/A: Not applicable.*

[a] : p-value is produced by comparing the given model to the base model.

Model	Cohorts	Sample	Effect	p	I^2	Sensitivity (p)
Base model (most inclusive)	k=14	N=2153	0.99 [0.82, 1.20]	0.95	92%	N/A
Large trials (n>100)	k=8	N=1860	1.01 [0.77, 1.31]	0.96	95%	p=0.94[a]
Most strict model (Moderate RoB, large trial and only adult cohorts)	k=2	N=577	1.34 [0.67, 2.70]	0.41	95%	p=0.42[a]
Most positive trial excluded	k=13	N=2047	0.95 [0.79, 1.14]	0.55	91%	p=0.71[a]
Most negative trial excluded	k=13	N=1619	1.06 [0.92, 1.21]	0.41	80%	p=0.59[a]
Adult cohorts	k=8	N=1471	0.92 [0.68, 1.24]	0.57	93%	p=0.25
Pediatric cohorts	k=5	N=536	1.14 [0.92, 1.55]	0.23	87%	
Trials with Moderate RoB grade	k=7	N=1113	1.18 [0.98, 1.43]	0.09	88%	p=0.03
Trials with Serious RoB grade	k=7	N=1050	0.81 [0.61, 1.07]	0.13	88%	
East Asian cohort	k=8	N=696	1.12 [0.92, 1.38]	0.27	87%	p=0.14
(Predominantly) Caucasian cohorts	k=5	N=1379	0.84 [0.62, 1.16]	0.29	93%	
Healthy volunteer cohorts	k=0	N=0	N/A	N/A	N/A	N/A
Neurology patient cohorts	k=13	N=2007	1.00 [0.88, 1.14]	0.96	93%	

© 2024 Milosavljević F et al. *JAMA Network Open.*

*Table S26. Sensitivity analysis: Lamotrigine C/D in UGT2B7*2 Heterozygous carriers vs *2 non-carriers.* Sensitivity analysis demonstrated that reported meta-analysis results did not significantly change: 1) In trials on both Caucasian and East Asian cohorts; 2) in trials with different RoB grades; 3) if the most positive or negative trials were excluded; or 4) if only large cohorts (total number of subjects is more than 100) were included. Since no trials on healthy volunteers were included, and since great majority of trials were conducted on adults; there was no enough data for all planned sensitivity analyses. It is hard to ascertain high level of certainty regarding the result robustness as the data is limited, but for the cases for which the data is available, results are highly robust. Not enough trials were included in the meta-analysis of lamotrigine C/D in UGT2B7*2 Homozygous carriers vs *2 non-carriers to perform a meaningful sensitivity analysis. *RoB: Risk-of-bias; N/A: Not applicable.*

[a] : p-value is produced by comparing the given model to the base model.

Model	Cohorts	Sample	Effect	p	I^2	Sensitivity (p)
Base model (most inclusive)	k=8	N=959	1.03 [0.96, 1.11]	0.36	10%	N/A
Large trials (n>100)	k=4	N=785	1.03 [0.96, 1.11]	0.35	0%	p=0.99[a]
Most strict model (Moderate RoB, large trial and only adult cohorts)	k=2	N=488	1.06 [0.97, 1.16]	0.22	0%	p=0.70[a]
Most positive trial excluded	k=7	N=827	1.01 [0.92, 1.11]	0.79	12%	p=0.72[a]
Most negative trial excluded	k=7	N=864	1.05 [0.98, 1.12]	0.17	0%	p=0.79[a]
Adult cohorts	k=7	N=809	1.04 [0.95, 1.14]	0.44	22%	p=0.83
Pediatric cohorts	k=1	N=150	1.02 [0.87, 1.19]	0.84	N/A	
Trials with Moderate RoB grade	k=3	N=638	1.05 [0.97, 1.13]	0.25	0%	p=0.90
Trials with Serious RoB grade	k=5	N=321	1.03 [0.87, 1.23]	0.71	41%	
East Asian cohort	k=4	N=336	1.09 [0.97, 1.22]	0.15	23%	p=0.30
(Predominantly) Caucasian cohorts	k=3	N=528	1.00 [0.90, 1.12]	0.99	0%	
Healthy volunteer cohorts	k=0	N=0	N/A	N/A	N/A	N/A
Neurology patient cohorts	k=8	N=959	1.03 [0.96, 1.11]	0.36	10%	

6.4) Sensitivity analyses: Carbamazepine (Tables S27-29)

Table S27. Sensitivity analysis: Carbamazepine C/D in CYP3A5 expressors and non-expressors. Sensitivity analysis demonstrated that reported meta-analysis results did not significantly change: 1) if the most positive or negative trials were excluded; or 2) if only large cohorts (total number of subjects is more than 100) were included. Since no included trials were conducted on healthy volunteers or children, and since the great majority of included trials were conducted East-Asian cohorts and had Serious RoB grade; there was no enough data for all planned sensitivity analyses. It is hard to ascertain high level of certainty regarding the result robustness as the data is limited, but for the cases for which the data is available, results are highly robust *RoB: Risk-of-bias; N/A: Not applicable.*

[a] : p-value is produced by comparing the given model to the base model.

Model	Trials	Sample	Effect	p	I^2	Sensitivity (p)
Base model (most inclusive)	k=13	N=1152	1.12 [1.03, 1.22]	0.007	66%	N/A
Large trials (n>100)	k=5	N=724	1.08 [0.98, 1.18]	0.12	63%	p=0.52[a]
Most strict model (Moderate RoB, large trial and only adult cohorts)	k=1	N=166	1.02 [0.94, 1.11]	0.65	N/A	p=0.11[a]
Most positive trial excluded	k=12	N=1068	1.09 [1.01, 1.18]	0.02	54%	p=0.68[a]
Most negative trial excluded	k=12	N=1008	1.14 [1.04, 1.24]	0.003	64%	p=0.78[a]
Adult cohorts	k=7	N=525	1.17 [1.03, 1.33]	0.02	70%	N/A
Pediatric cohorts	k=0	N=0	N/A	N/A	N/A	
Trials with Moderate or NI RoB grade	k=1	N=166	1.02 [0.94, 1.11]	0.65	N/A	p=0.09
Trials with Serious RoB grade	k=12	N=986	1.14 [1.04, 1.24]	0.007	65%	
East Asian cohort	k=12	N=1052	1.10 [1.02, 1.20]	0.02	65%	p=0.05
South Indian cohorts	k=1	N=100	1.59 [1.11, 2.29]	0.01	N/A	
Healthy volunteer cohorts	k=0	N=0	N/A	N/A	N/A	N/A
Neurology patient cohorts	k=12	N=1085	1.13 [1.05, 1.23]	0.002	66%	

© 2024 Milosavljević F et al. *JAMA Network Open.*

Table S28. Sensitivity analysis: Carbamazepine C/D in EPHX1 337TT and 337CT carriers. Sensitivity analysis demonstrated that reported meta-analysis results did not significantly change: 1) in trials with different RoB grades; 2) if the most positive or negative trials were excluded; or 3) if only large cohorts (total number of subjects is more than 100) were included. Since no included trials were conducted on healthy volunteers or children, and since the great majority of included trials were conducted East-Asian cohorts; there was not enough data for all planned sensitivity analyses. It is hard to ascertain high level of certainty regarding the result robustness as the data is limited, but for the cases for which the data is available, results are highly robust. *RoB: Risk-of-bias; N/A: Not applicable.*

[a] : p-value is produced by comparing the given model to the base model.

Model	Trials	Sample	Effect	p	I^2	Sensitivity (p)
Base model (most inclusive)	k=7	N=869	0.91 [0.78, 1.06]	0.23	83%	N/A
Large trials (n>100)	k=4	N=621	0.83 [0.70, 0.99]	0.04	86%	p=0.44[a]
Most strict model (Moderate RoB, large trial and only adult cohorts)	k=2	N=267	0.91 [0.69, 1.19]	0.49	84%	p=0.98[a]
Most positive trial excluded	k=6	N=771	0.87 [0.75, 1.01]	0.07	81%	p=0.68[a]
Most negative trial excluded	k=6	N=698	0.96 [0.85, 1.09]	0.55	68%	p=0.59[a]
Adult cohorts	k=6	N=686	0.92 [0.76, 1.10]	0.36	85%	N/A
Pediatric cohorts	k=0	N=0	N/A	N/A	N/A	
Trials with Moderate or NI RoB grade	k=4	N=459	1.00 [0.81, 1.22]	0.97	80%	p=0.23
Trials with Serious RoB grade	k=3	N=410	0.82 [0.63, 1.05]	0.12	86%	
East Asian cohort	k=4	N=542	0.81 [0.69, 0.94]	0.007	80%	p=0.03
(Predominantly) Caucasian cohorts	k=1	N=135	1.06 [0.87, 1.28]	0.57	N/A	
South Indian cohorts	k=1	N=98	1.24 [0.96, 1.61]	0.09	N/A	
North African cohorts	k=1	N=94	1.01 [0.82, 1.25]	0.92	N/A	
Healthy volunteer cohorts	k=0	N=0	N/A	N/A	N/A	N/A
Neurology patient cohorts	k=7	N=869	0.91 [0.78, 1.06]	0.23	83%	

© 2024 Milosavljević F et al. *JAMA Network Open.*

Table S29. Sensitivity analysis: Carbamazepine C/D in EPHX1 337TT and 337CC carriers. Since no included trials were conducted on healthy volunteers or children, and since the great majority of included trials were conducted East-Asian cohorts; there was no enough data for all planned sensitivity analyses. There is an indication that trails with different RoB grades produce different results and that exclusion of the most positive and most negative trail leads to the significant changes in the results, but due to the low number of overall included trials it is hard to claim certainty of the observed effects. *RoB: Risk-of-bias; N/A: Not applicable.*

[a] : p-value is produced by comparing the given model to the base model.

Model	Trials	Sample	Effect	p	I^2	Sensitivity (p)
Base model (most inclusive)	k=7	N=574	0.93 [0.67, 1.29]	0.66	95%	N/A[a]
Large trials (n>100)	k=1	N=102	1.79 [1.54, 2.08]	<0.001	N/A	p<0.001[a]
Most positive trial excluded	k=6	N=472	0.81 [0.65, 1.02]	0.07	87%	p=0.45[a]
Most negative trial excluded	k=6	N=453	1.03 [0.77, 1.38]	0.85	93%	p=0.65[a]
Adult cohorts	k=6	N=479	0.92 [0.63, 1.35]	0.68	96%	N/A
Pediatric cohorts	k=0	N=0	N/A	N/A	N/A	
Trials with Moderate or NI RoB grade	k=4	N=312	1.10 [0.72, 1.69]	0.66	95%	p=0.21
Trials with Serious RoB grade	k=3	N=262	0.75 [0.50, 1.13]	0.17	91%	
East Asian cohort	k=4	N=347	0.79 [0.58, 1.06]	0.12	92%	p<0.0001
(Predominantly) Caucasian cohorts	k=1	N=73	0.78 [0.65, 0.94]	0.01	N/A	
South Indian cohorts	k=1	N=52	1.18 [0.64, 2.17]	0.59	N/A	
North African cohorts	k=1	N=102	1.79 [1.54, 2.08]	<0.001	N/A	
Healthy volunteer cohorts	k=0	N=0	N/A	N/A	N/A	N/A
Neurology patient cohorts	k=7	N=574	0.93 [0.67, 1.29]	0.66	95%	

7) Forest plots – SMD (Standardized mean difference)

7.1) Phenytoin related SMD meta-analyses (Figures S31-S34)

Study or Subgroup	Experimental Mean	Experimental SD	Experimental Total	Control Mean	Control SD	Control Total	Weight	Std. Mean Difference IV, Random, 95% CI
Soga 2004	5.253219	0.646475	3	2.990215	1.682024	25	2.8%	1.35 [0.09, 2.61]
Ortega-Vázquez 2015	2.8	1.2	3	3	3.2	61	3.1%	-0.06 [-1.22, 1.10]
Mamiya 1998	2.2	0.8	3	2.050382	1.057601	131	3.1%	0.14 [-1.00, 1.29]
Sharma 2015	4.041	1.29	9	2.145	0.82	26	4.0%	1.94 [1.04, 2.84]
Li Z 2016	2.066364	0.580853	6	1.396404	0.87635	56	4.2%	0.77 [-0.08, 1.63]
Caraco 2001	749.5	263.5852	12	472	117	18	4.3%	1.43 [0.60, 2.26]
Rosemary 2006	4.10625	2.709316	16	3.925	2.458764	10	4.5%	0.07 [-0.72, 0.86]
Lee 2007	3.36	0.9	9	1.876782	0.89626	87	4.8%	1.64 [0.92, 2.37]
Lin 2008	0.066971	0.035903	10	0.047241	0.022231	43	4.9%	0.77 [0.07, 1.48]
George 2012	4.827273	0.737687	11	5.2	1.8	36	5.0%	-0.23 [-0.90, 0.45]
Guevara 2017	2.121688	0.955108	16	1.631765	1.024572	34	5.3%	0.48 [-0.12, 1.08]
Yamamoto 2015	3.543224	0.855342	13	2.537579	0.636205	157	5.4%	1.53 [0.94, 2.12]
van der Weide 2001	0.074919	0.027607	21	0.056879	0.032451	37	5.6%	0.58 [0.03, 1.12]
Wanounou 2022	16.81202	3.872183	16	11.06107	4.580153	127	5.6%	1.27 [0.73, 1.81]
Hung 2012	4.491553	1.882532	16	2.768079	1.16394	252	5.7%	1.41 [0.89, 1.93]
Hung 2004	4.388889	1.786514	18	3.012583	1.209121	151	5.8%	1.07 [0.57, 1.57]
Aynacioglu 1999	5.684375	1.881113	32	4.16	1.262175	68	6.1%	1.02 [0.57, 1.46]
Kesavan 2010	4	1.504728	40	1.896721	0.909229	244	6.4%	2.07 [1.70, 2.45]
Shaul 2022	3.610448	1.044703	67	2.4	0.7	69	6.4%	1.36 [0.98, 1.73]
Fohner 2019	13.69432	7.402723	88	8.8	5.2	231	6.9%	0.83 [0.58, 1.08]
**Total (95% CI)**			**409**			**1863**	**100.0%**	**1.01 [0.75, 1.28]**

Heterogeneity: Tau² = 0.25; Chi² = 78.84, df = 19 (P < 0.00001); I² = 76%
Test for overall effect: Z = 7.47 (P < 0.00001)

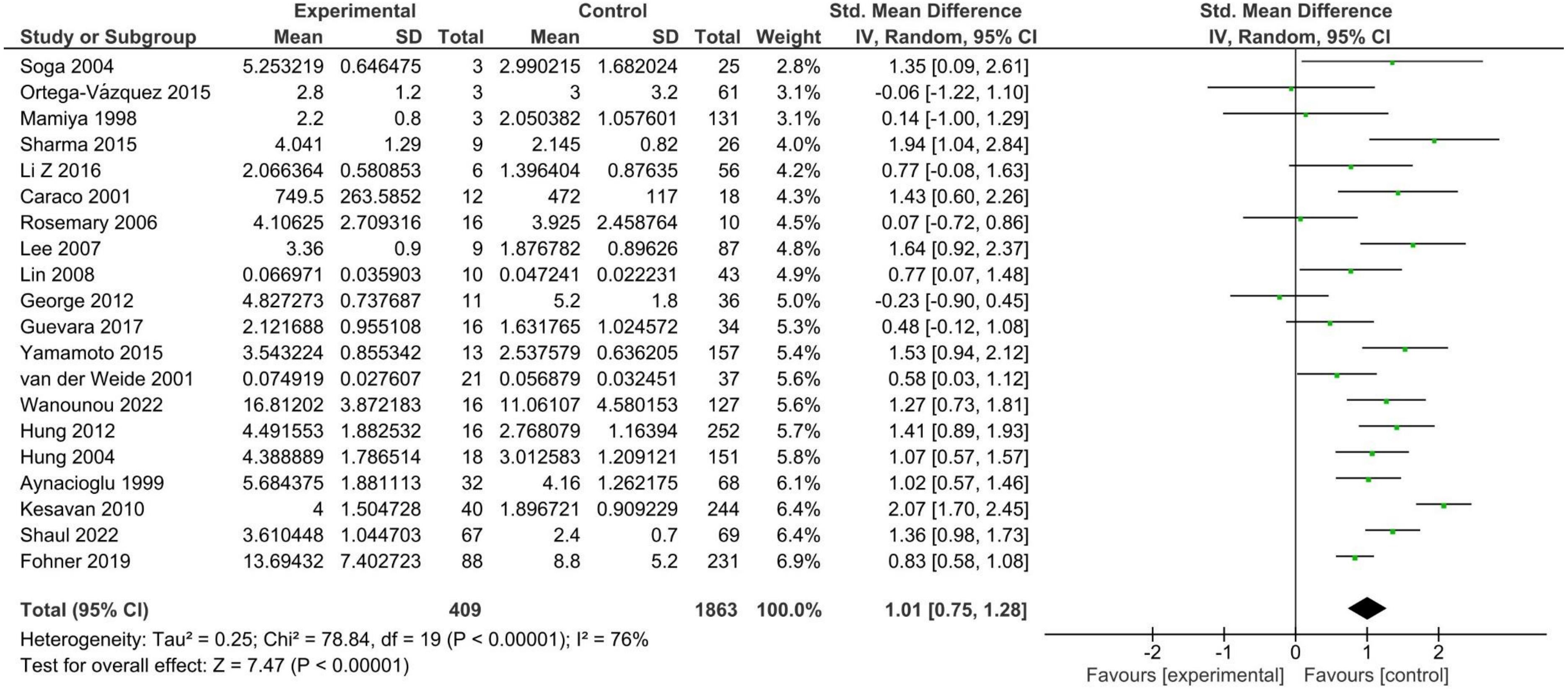

Figure S31. Forest plot (SMD): Phenytoin C/D in CYP2C9 IMs compared to CYP2C9 NMs. Based on the data from k=20 trials and N=2272 patients, large effect of CYP2C9 IMs status (SMD: 1.01 [95%CI: 0.75]; 1.28; df=19; p<0.001) on phenytoin C/D was observed. Considerable heterogeneity was observed (I^2=76%). *C/D: Plasma concentration-to-dose ratio; IMs: Intermediate metabolizers; NMs: Normal metabolizers (reference group).*

© 2024 Milosavljević F et al. *JAMA Network Open.*

Study or Subgroup	Experimental Mean	Experimental SD	Experimental Total	Control Mean	Control SD	Control Total	Weight	Std. Mean Difference IV, Random, 95% CI
Yamanaka 2005	2.339778	1.321673	3	1.706072	0.726766	10	1.8%	0.68 [-0.65, 2.01]
Soga 2004	3.115044	1.270568	13	2.954277	1.136896	3	2.0%	0.12 [-1.13, 1.38]
Taguchi 2005	2.511795	1.398115	9	1.722247	0.642819	5	2.5%	0.62 [-0.51, 1.74]
Huang 2004	3	0.9	14	2.6	0.8	11	4.4%	0.45 [-0.35, 1.25]
Ortega-Vázquez 2015	2.7	1.6	7	3	3.4	52	4.6%	-0.09 [-0.88, 0.70]
Guevara 2017	1.9038	0.899556	10	1.759725	1.055969	40	5.6%	0.14 [-0.56, 0.83]
Lee 2007	2.06	0.9	44	1.45	0.6	36	9.8%	0.77 [0.32, 1.23]
Yamamoto 2015	2.558811	0.660802	75	2.136306	0.404734	47	12.0%	0.73 [0.35, 1.10]
Mamiya 1998	2.3	1.2	64	1.7	0.8	52	12.1%	0.57 [0.20, 0.95]
Hung 2004	3	1.2	88	2.7	1.1	47	12.7%	0.26 [-0.10, 0.61]
Kesavan 2010	2.333077	6.405131	130	2.120388	1.185723	103	16.2%	0.04 [-0.21, 0.30]
Hung 2012	57.65646	26.21185	150	43.86521	16.41571	102	16.2%	0.60 [0.35, 0.86]
**Total (95% CI)**			**607**			**508**	**100.0%**	**0.42 [0.24, 0.61]**

Heterogeneity: $Tau^2 = 0.04$; $Chi^2 = 19.18$, df = 11 (P = 0.06); $I^2 = 43\%$
Test for overall effect: Z = 4.43 (P < 0.00001)

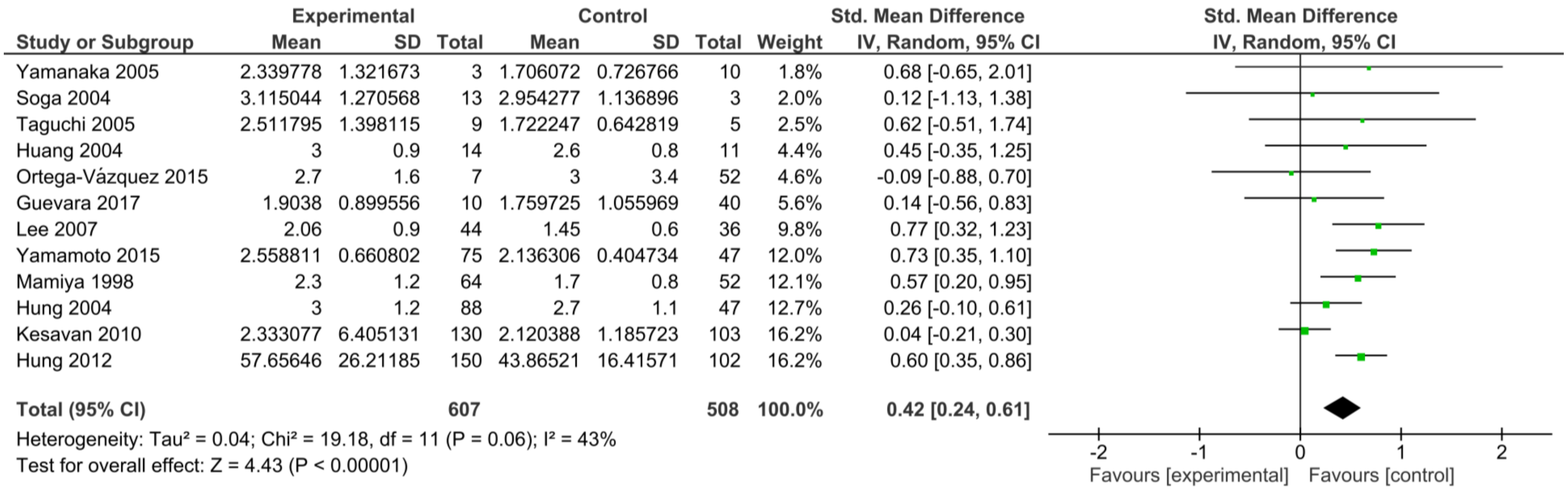

Figure S32. Forest plot (SMD): Phenytoin C/D in CYP2C19 IMs compared to CYP2C19 NMs. Based on the data from k=12 trials and N=1115 patients, CYP2C19 IMs status had moderate effect (SMD: 0.42 [95%CI: 0.24, 0.61]; df=11; p<0.001) on phenytoin C/D. Low heterogeneity was observed (I^2=43%). *C/D: Plasma concentration-to-dose ratio; IMs: Intermediate metabolizers; NMs: Normal metabolizers (reference group).*

© 2024 Milosavljević F et al. *JAMA Network Open.*

Study or Subgroup	Experimental Mean	Experimental SD	Experimental Total	Control Mean	Control SD	Control Total	Weight	Std. Mean Difference IV, Random, 95% CI
Soga 2004	2.813668	1.531428	9	2.954277	1.136896	3	8.1%	-0.09 [-1.40, 1.22]
Taguchi 2005	1.960438	1.255185	6	1.722247	0.642819	5	8.9%	0.21 [-0.98, 1.40]
Lee 2007	2.92	1	7	1.45	0.6	36	10.8%	2.14 [1.20, 3.08]
Hung 2004	4	1.1	16	2.7	1.1	47	13.7%	1.17 [0.56, 1.77]
Mamiya 1998	2.2	0.9	15	1.7	0.8	52	13.9%	0.60 [0.02, 1.18]
Hung 2012	53.45285	13.01565	15	43.86521	16.41571	102	14.2%	0.59 [0.05, 1.14]
Yamamoto 2015	3.102741	0.742701	35	2.136306	0.404734	47	14.5%	1.67 [1.16, 2.18]
Kesavan 2010	2.730508	5.479906	59	2.120388	1.185723	103	15.9%	0.18 [-0.14, 0.50]
**Total (95% CI)**			**162**			**395**	**100.0%**	**0.84 [0.32, 1.36]**

Heterogeneity: Tau² = 0.41; Chi² = 37.78, df = 7 (P < 0.00001); I² = 81%
Test for overall effect: Z = 3.19 (P = 0.001)

Std. Mean Difference IV, Random, 95% CI

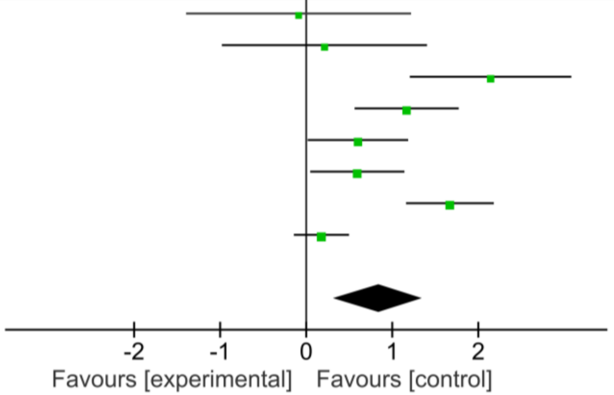

Figure S33. Forest plot (SMD): Phenytoin C/D in CYP2C19 PMs compared to CYP2C19 NMs. Based on the data from k=8 trials and N=557 patients, CYP2C19 PMs status had a large effect (SMD:0.84; CI95%: 0.32, 1.36; df=7; p=0.001) on phenytoin C/D. Substantial heterogeneity was observed (I^2=81%). *C/D: Plasma concentration-to-dose ratio; PMs: Poor metabolizers; NMs: Normal metabolizers.*

© 2024 Milosavljević F et al. *JAMA Network Open.*

	Experimental			Control				Std. Mean Difference
Study or Subgroup	Mean	SD	Total	Mean	SD	Total	Weight	IV, Random, 95% CI
Soga 2004	2.991754	1.356068	22	2.954277	1.136896	3	3.5%	0.03 [-1.18, 1.23]
Taguchi 2005	2.291252	1.325832	15	1.722247	0.642819	5	4.6%	0.45 [-0.57, 1.48]
Lee 2007	2.178039	0.951805	51	1.45	0.6	36	12.5%	0.87 [0.43, 1.32]
Mamiya 1998	2.281013	1.144561	79	1.7	0.8	52	14.7%	0.57 [0.21, 0.92]
Yamamoto 2015	2.731879	0.730261	110	2.136306	0.404734	47	14.7%	0.91 [0.55, 1.27]
Hung 2004	3.153846	1.23449	104	2.7	1.1	47	14.9%	0.38 [0.03, 0.73]
Hung 2012	57.27431	25.3012	165	43.86521	16.41571	102	17.4%	0.60 [0.35, 0.85]
Kesavan 2010	2.457143	6.119566	189	2.120388	1.185723	103	17.7%	0.07 [-0.17, 0.31]
**Total (95% CI)**			**735**			**395**	**100.0%**	**0.52 [0.27, 0.77]**

Heterogeneity: Tau² = 0.07; Chi² = 21.87, df = 7 (P = 0.003); I² = 68%
Test for overall effect: Z = 4.13 (P < 0.0001)

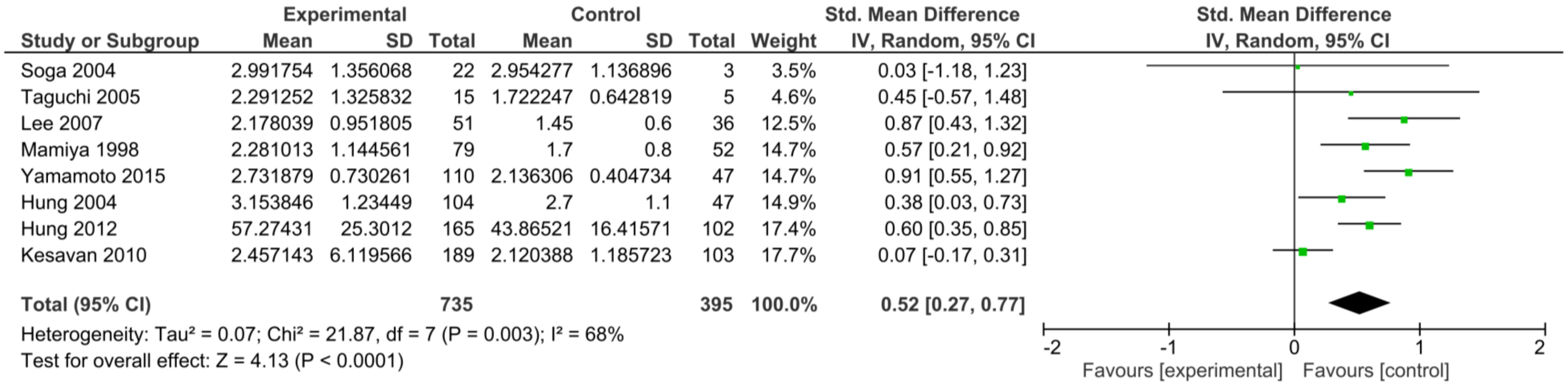

Figure S34. Forest plot (SMD): Phenytoin C/D in combined CYP2C19 IMs and PMs compared to CYP2C19 NMs. Based on the data from k=8 trials and N=1130 patients, carrying either CYP2C19 IM or PM status had a moderate effect (SMD: 0.52 [95%CI: 0.27, 0.77]; df=7; p<0.001) on phenytoin C/D. Moderate heterogeneity was observed (I^2=68%). *C/D: Plasma concentration-to-dose ratio; PMs: Poor metabolizers; IMs: Intermediate metabolizers; NMs: Normal metabolizers (reference group).*

© 2024 Milosavljević F et al. *JAMA Network Open.*

7.2) Valproic acid related SMD meta-analyses (Figures S35-S43)

Study or Subgroup	Experimental Mean	Experimental SD	Experimental Total	Control Mean	Control SD	Control Total	Weight	Std. Mean Difference IV, Random, 95% CI
Mei 2017	2.952888	1.56928	4	3.18748	1.78506	43	3.7%	-0.13 [-1.16, 0.89]
Li Z 2017	92.64	23.6	5	66.58	28.52	79	4.2%	0.91 [-0.00, 1.83]
Rutigliano 2013	5.5	1.44	7	5.27	1.88	62	5.1%	0.12 [-0.66, 0.91]
Wang 2017	2.72	1.46	7	2.81	1.16	95	5.2%	-0.08 [-0.84, 0.69]
Song 2020	3.91	0.99	8	4.08	1.97	75	5.5%	-0.09 [-0.82, 0.64]
Tóth 2015	4.857241	2.65384	15	4.02538	1.80632	35	6.5%	0.39 [-0.22, 1.00]
Xu 2021	2.58	1.34022	11	2.65667	1.87573	181	6.5%	-0.04 [-0.65, 0.57]
Zhao 2017	3.95	1.11	11	3.16	114	189	6.5%	0.01 [-0.60, 0.61]
Wu 2021	2.999673	1.25239	15	2.67761	0.97363	91	7.1%	0.31 [-0.23, 0.86]
Tan 2010	3.9	0.4	15	3.4	0.4	164	7.1%	1.24 [0.70, 1.79]
Liao 2013	4.35	1.48	16	2.80991	1.24867	115	7.1%	1.20 [0.65, 1.74]
Feng 2018	2.528804	1.48453	18	3.00524	1.64476	156	7.6%	-0.29 [-0.78, 0.20]
Du 2021	2.65	0.99379	21	2.17333	0.73159	208	8.0%	0.63 [0.17, 1.08]
Smith 2016	0.33747	0.15691	67	0.33914	0.22195	182	9.7%	-0.01 [-0.29, 0.27]
Sun 2017	4.15	1.32	104	4.04	1.5	285	10.2%	0.08 [-0.15, 0.30]
**Total (95% CI)**			**324**			**1960**	**100.0%**	**0.29 [0.05, 0.52]**

Heterogeneity: Tau² = 0.13; Chi² = 43.05, df = 14 (P < 0.0001); I² = 67%
Test for overall effect: Z = 2.34 (P = 0.02)

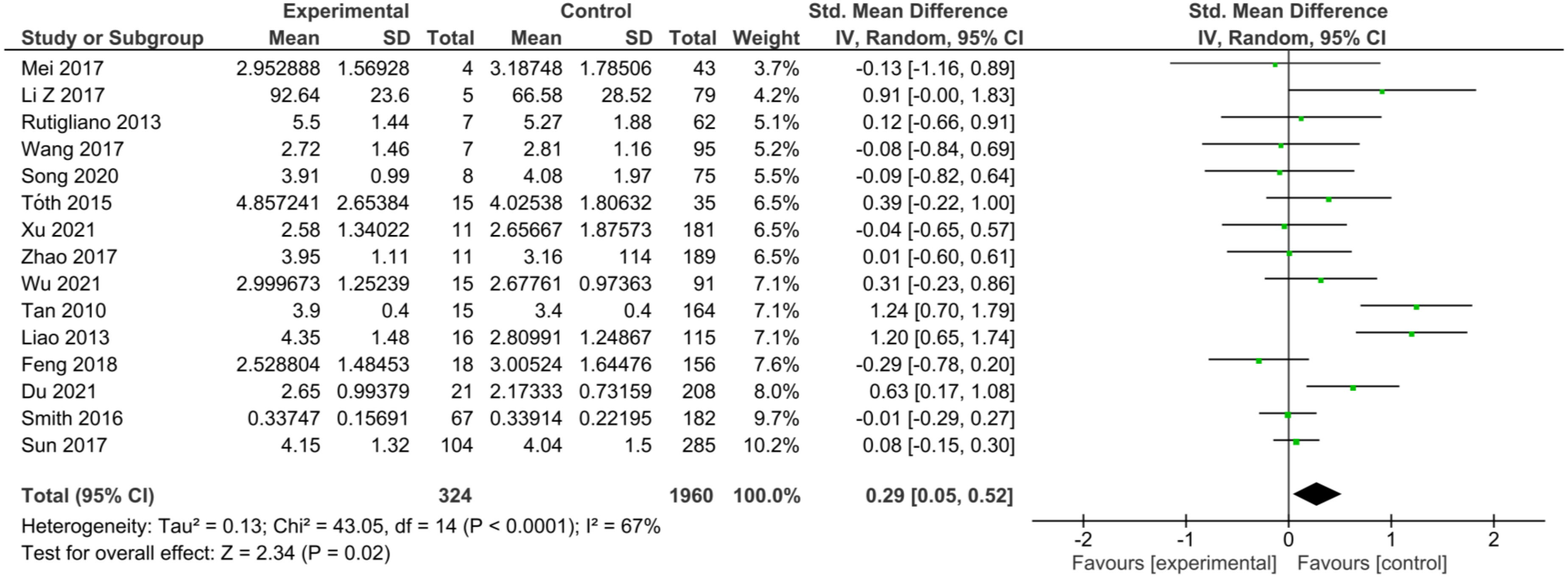

Figure S35. Forest plot (SMD): Valproic acid C/D in CYP2C9 IMs compared to CYP2C9 NMs. Based on the data from k=15 trials and N=2287 patients, CYP2C9 IMs status had a small effect (SMD:0.29 [95%: 0.05, 0.52]; df=14; p=0.021) on valproic acid C/D. Moderate heterogeneity was observed (I^2=68%). *C/D: Plasma concentration-to-dose ratio; IMs: Intermediate metabolizers; NMs: Normal metabolizers (reference group).*

© 2024 Milosavljević F et al. *JAMA Network Open.*

Study or Subgroup	Experimental Mean	Experimental SD	Experimental Total	Control Mean	Control SD	Control Total	Weight	Std. Mean Difference IV, Random, 95% CI
Jogamoto 2017	4.244667	1.307068	15	3.912222	1.453391	9	4.9%	0.24 [-0.59, 1.07]
Mei 2017	3.402961	1.97038	23	2.701398	1.435913	14	6.1%	0.38 [-0.29, 1.05]
Zheng 2023	8.846111	7.745786	18	3.835652	2.176678	23	6.2%	0.92 [0.26, 1.57]
Feng 2016	2.57167	1.267011	28	2.033205	1.10006	18	6.7%	0.44 [-0.16, 1.04]
Song 2020	4.45	1.42	33	3.33	1.78	37	7.9%	0.68 [0.20, 1.17]
Liao 2013	2.76	1.19	56	2.57	1.3	44	8.8%	0.15 [-0.24, 0.55]
Feng 2018	2.953692	1.77463	85	2.921902	1.409261	60	9.4%	0.02 [-0.31, 0.35]
Han 2015	2.71	0.33	77	2.69	0.48	72	9.5%	0.05 [-0.27, 0.37]
Smith 2016	0.34268	0.254981	74	0.344179	0.210333	96	9.7%	-0.01 [-0.31, 0.30]
Du 2021	2.2	0.676337	107	2.296667	0.940256	100	10.0%	-0.12 [-0.39, 0.15]
Wang 2021	131.51	48.15	137	90.96	46.46	118	10.2%	0.85 [0.60, 1.11]
Sun 2017	3.92	1.26	173	4.15	1.3	177	10.6%	-0.18 [-0.39, 0.03]
**Total (95% CI)**			**826**			**768**	**100.0%**	**0.25 [0.01, 0.49]**

Heterogeneity: Tau² = 0.13; Chi² = 55.21, df = 11 (P < 0.00001); I² = 80%
Test for overall effect: Z = 2.04 (P = 0.04)

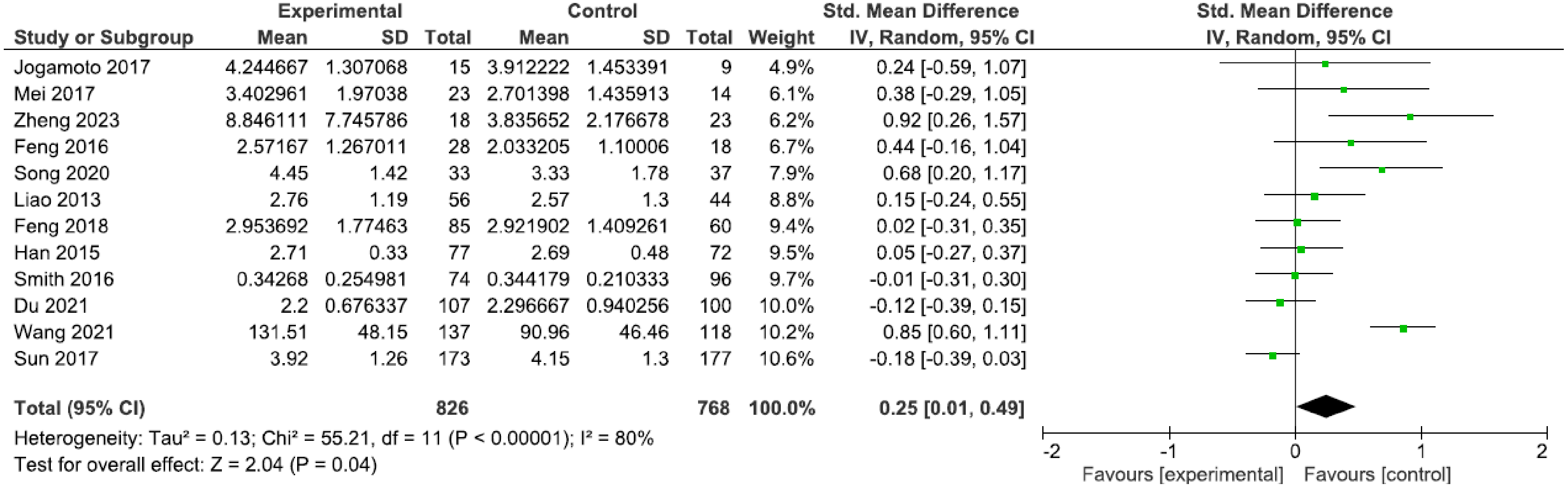

Figure S36: Forest plot (SMD): Valproic acid C/D in CYP2C19 IMs compared to CYP2C19 NMs. Based on the data from k=12 trials and N=1594 patients, CYP2C19 IMs status had a small effect (SMD: 0.25 [95%CI: 0.01, 0.49]; df=11; p=0.041) on valproic acid C/D. Substantial heterogeneity was observed (I^2=80%). *C/D: Plasma concentration-to-dose ratio; IMs: Intermediate metabolizers; NMs: Normal metabolizers (reference group).*

© 2024 Milosavljević F et al. *JAMA Network Open.*

Study or Subgroup	Experimental Mean	Experimental SD	Experimental Total	Control Mean	Control SD	Control Total	Weight	Std. Mean Difference IV, Random, 95% CI
Jogamoto 2017	3.085	0.433321	4	3.912222	1.453391	9	5.7%	-0.61 [-1.82, 0.60]
Zheng 2023	9.768	7.731437	5	3.835652	2.176678	23	6.4%	1.58 [0.52, 2.65]
Mei 2017	3.278553	1.65877	10	2.701398	1.435913	14	7.6%	0.36 [-0.46, 1.18]
Feng 2016	1.952574	0.696243	10	2.033205	1.10006	18	7.8%	-0.08 [-0.85, 0.69]
Song 2020	6.64	1.06	13	3.33	1.78	37	7.9%	2.00 [1.25, 2.75]
Smith 2016	0.31371	0.045029	9	0.344179	0.210333	96	8.3%	-0.15 [-0.83, 0.53]
Liao 2013	3.7	0.95	15	2.57	1.3	44	8.7%	0.91 [0.30, 1.52]
Han 2015	3.78	1.08	28	2.69	0.48	72	9.2%	1.55 [1.06, 2.04]
Du 2021	2.223333	0.530967	22	2.296667	0.940256	100	9.3%	-0.08 [-0.54, 0.38]
Feng 2018	3.101639	1.662868	28	2.921902	1.409261	60	9.4%	0.12 [-0.33, 0.57]
Wang 2021	141.89	65.06	41	90.96	46.46	118	9.7%	0.98 [0.61, 1.35]
Sun 2017	4.03	1.41	51	4.15	1.3	177	9.9%	-0.09 [-0.40, 0.22]
**Total (95% CI)**			**236**			**768**	**100.0%**	**0.54 [0.11, 0.98]**

Heterogeneity: Tau² = 0.46; Chi² = 76.45, df = 11 (P < 0.00001); I² = 86%
Test for overall effect: Z = 2.48 (P = 0.01)

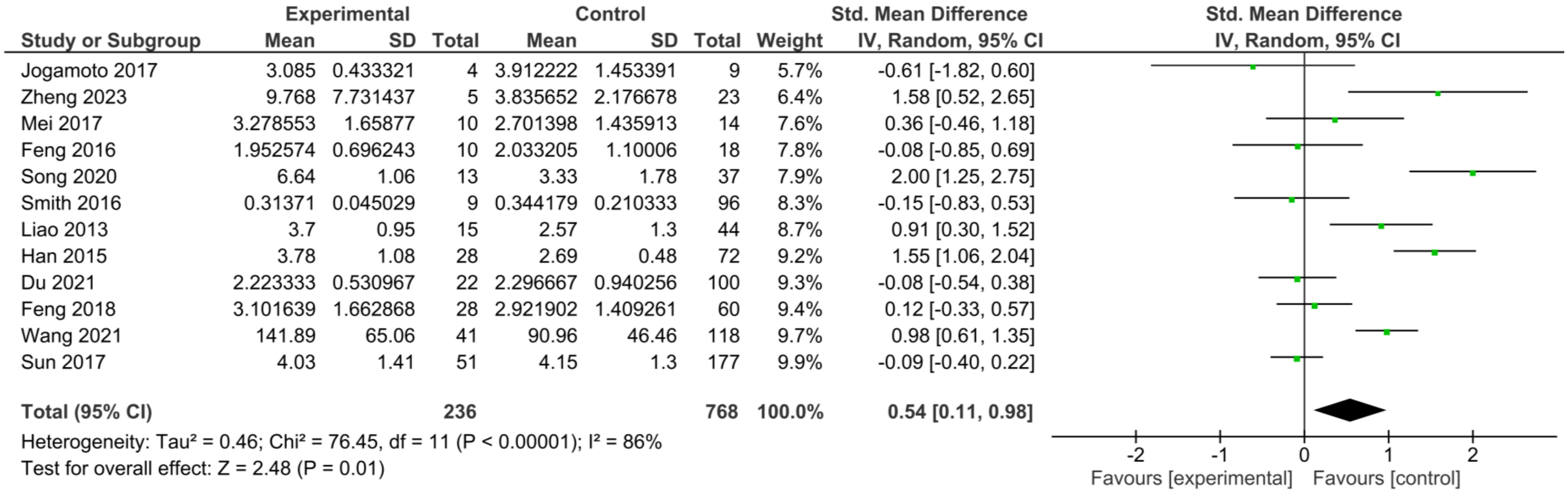

Figure S37. Forest plot (SMD): Valproic acid C/D in CYP2C19 PMs compared to CYP2C19 NMs. Based on the data from k=12 trials and N=1004 patients, CYP2C19 PMs status had moderate effect (SMD: 0.54 [95%CI: 0.11, 0.98]; df=11; p=0.013) on valproic acid C/D. High heterogeneity was observed (I^2=86%). *C/D: Plasma concentration-to-dose ratio; PMs: Poor metabolizers; NMs: Normal metabolizers (reference group).*

© 2024 Milosavljević F et al. *JAMA Network Open.*

Study or Subgroup	Experimental Mean	Experimental SD	Experimental Total	Control Mean	Control SD	Control Total	Weight	Std. Mean Difference IV, Random, 95% CI
Feng 2019	3.39343	1.19372	5	2.44897	1.5406	20	2.5%	0.61 [-0.38, 1.61]
Sun 2007	3.43	0.3	24	4.32	0.21	40	3.0%	-3.56 [-4.37, -2.74]
Zheng 2023	3.68833	1.66736	18	8.6932	7.5042	25	3.5%	-0.84 [-1.48, -0.21]
Aphichartphunkawee 2014	2.98	0.34	24	3.78	0.29	56	3.5%	-2.59 [-3.22, -1.96]
Algharably 2016	3.325	0.98936	16	3.575	1.4	25	3.5%	-0.19 [-0.82, 0.43]
Mei 2017	3.23494	1.55395	19	3.10977	1.9879	24	3.6%	0.07 [-0.53, 0.67]
Feng 2016	2.19002	0.92905	19	2.33549	1.2619	35	3.7%	-0.12 [-0.68, 0.44]
Jain 2015	3.9	3.03103	31	2.76667	1.853	36	3.9%	0.45 [-0.03, 0.94]
Jin 2013	2.21	0.66	28	3.06	0.8	69	4.0%	-1.11 [-1.57, -0.64]
Li Z 2017	63.84	27.62	37	71.72	29.94	45	4.1%	-0.27 [-0.71, 0.17]
Nandith 2021	5.22	2.8	61	6.21	3.9	31	4.1%	-0.31 [-0.74, 0.13]
Guo 2012	4.66	2.34	39	4.25	1.88	49	4.1%	0.19 [-0.23, 0.62]
Shen 2016	3.44	1.67	37	3.86	1.43	55	4.1%	-0.27 [-0.69, 0.15]
Zhao 2020	2.93	1.06	56	3.12	1.13	55	4.3%	-0.17 [-0.55, 0.20]
Kang 2016	2.73	1.34	61	3.18	1.67	55	4.3%	-0.30 [-0.66, 0.07]
Chatzistefanidis 2016	3.72475	1.98507	66	3.59682	1.8737	51	4.3%	0.07 [-0.30, 0.43]
Chu 2012	4.6	1.58	45	4.52	1.38	88	4.3%	0.05 [-0.30, 0.41]
Hung 2011	3.18678	0.9659	50	4.53459	1.8621	101	4.3%	-0.83 [-1.18, -0.47]
Sterjev 2012	0.42891	0.15364	61	0.46	0.19	69	4.3%	-0.18 [-0.52, 0.17]
Wang 2019	3,390.89	4,320	49	3,379.64	2,638	101	4.3%	0.00 [-0.34, 0.34]
Wang 2010	3.59	1.39	65	3.91	1.57	76	4.4%	-0.21 [-0.55, 0.12]
Feng 2018	3.02568	1.56312	67	2.86644	1.6573	99	4.4%	0.10 [-0.21, 0.41]
Xu 2021	2.28	1.34735	70	2.92667	2.1627	113	4.4%	-0.34 [-0.64, -0.04]
Du 2021	2.26333	0.71503	96	2.19667	0.7879	120	4.5%	0.09 [-0.18, 0.36]
Sun 2017	4.25	1.37	156	3.98	1.8	201	4.6%	0.17 [-0.04, 0.38]
**Total (95% CI)**			**1200**			**1639**	**100.0%**	**-0.34 [-0.56, -0.11]**

Heterogeneity: Tau² = 0.27; Chi² = 191.34, df = 24 (P < 0.00001); I² = 87%

Test for overall effect: Z = 2.95 (P = 0.003)

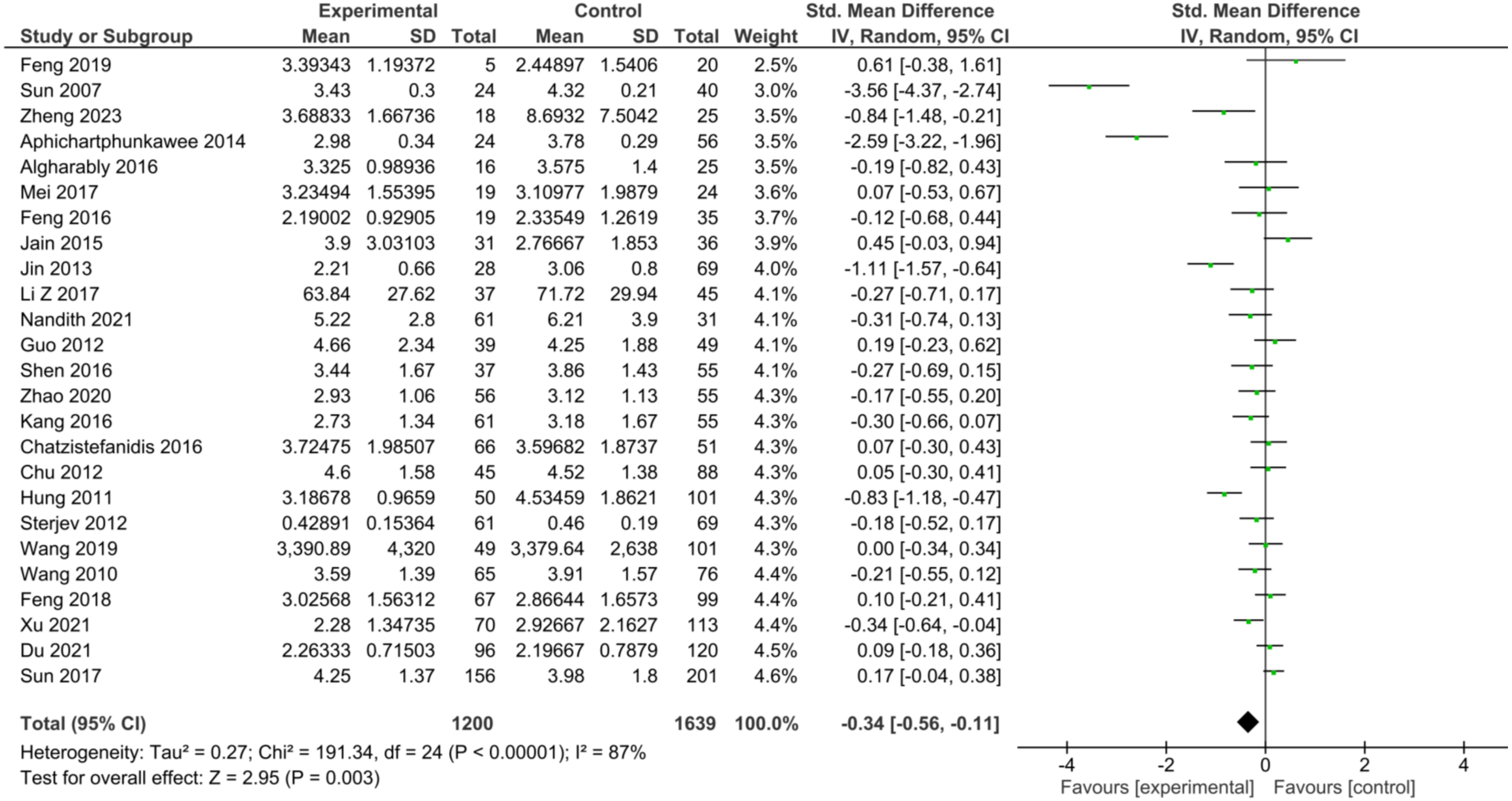

Figure S38. Forest plot: Valproic acid C/D in UGT1A6*2 heterozygous (*2 He) carriers and *2 non carriers (*2 None). Heterozygous carriers were defined as either 19GT, 541AG or 552CA carriers, while *2 non-carriers were defined as either 19TT, 541AA or 552AA carriers. Based on the data from k=25 trials and N=2839 patients, *2 heterozygous status had a small negative effect (SMD: -0.34 [95%CI: -0.56, -0.11]; df=24; p=0.003) on valproic acid C/D. High heterogeneity was observed (I^2=87%). *C/D: Plasma concentration-to-dose ratio.*

© 2024 Milosavljević F et al. *JAMA Network Open.*

Study or Subgroup	Experimental Mean	Experimental SD	Experimental Total	Control Mean	Control SD	Control Total	Weight	Std. Mean Difference IV, Random, 95% CI
Sun 2007	3.16	0.84	3	4.32	0.21	40	2.7%	-4.12 [-5.61, -2.63]
Zheng 2023	3.66	0.15556	2	8.6932	7.5042	25	2.7%	-0.66 [-2.12, 0.79]
Li Z 2017	66.87	26.91	2	71.72	29.94	45	2.8%	-0.16 [-1.58, 1.26]
Aphichartphunkawee 2014	5.54	1.32	4	3.78	0.29	56	3.1%	4.21 [2.94, 5.49]
Feng 2019	4.16195	1.68683	3	2.44897	1.5406	20	3.2%	1.06 [-0.20, 2.32]
Chu 2012	5.92	2.72	3	4.52	1.38	88	3.4%	0.97 [-0.18, 2.13]
Mei 2017	2.38495	1.07477	4	3.10977	1.9879	24	3.6%	-0.37 [-1.43, 0.69]
Feng 2016	2.16608	1.0537	4	2.33549	1.2619	35	3.7%	-0.13 [-1.17, 0.90]
Shen 2016	2.76	1.06	5	3.86	1.43	55	4.0%	-0.77 [-1.70, 0.16]
Algharably 2016	2.15	1.02603	7	3.575	1.4	25	4.1%	-1.04 [-1.92, -0.16]
Guo 2012	2.61	1.7	6	4.25	1.88	49	4.2%	-0.87 [-1.73, -0.00]
Wang 2010	3.73	1.28	6	3.91	1.57	76	4.3%	-0.11 [-0.95, 0.72]
Nandith 2021	5.32	3.3	7	6.21	3.9	31	4.3%	-0.23 [-1.05, 0.59]
Wang 2019	2,633.29	3,937.97	7	3,379.64	2,638	101	4.5%	-0.27 [-1.04, 0.50]
Feng 2018	3.47981	1.95257	8	2.86644	1.6573	99	4.6%	0.36 [-0.36, 1.08]
Zhao 2020	3.32	1.16	11	3.12	1.13	55	4.8%	0.17 [-0.47, 0.82]
Xu 2021	3.07333	2.72621	10	2.92667	2.1627	113	4.8%	0.07 [-0.58, 0.71]
Hung 2011	2.65657	0.98333	11	4.53459	1.8621	101	4.8%	-1.04 [-1.67, -0.40]
Jain 2015	2.26667	2.40861	13	2.76667	1.853	36	4.8%	-0.24 [-0.88, 0.39]
Kang 2016	3.24	1.7	13	3.18	1.67	55	4.9%	0.04 [-0.57, 0.64]
Du 2021	2.28333	0.87208	13	2.19667	0.7879	120	5.0%	0.11 [-0.46, 0.68]
Chatzistefanidis 2016	4.07553	2.17201	17	3.59682	1.8737	51	5.0%	0.24 [-0.31, 0.79]
Sterjev 2012	0.40167	0.15381	23	0.46374	0.1875	69	5.2%	-0.34 [-0.82, 0.13]
Sun 2017	4.17	1.37	38	3.98	1.8	201	5.5%	0.11 [-0.24, 0.46]
**Total (95% CI)**			**220**			**1570**	**100.0%**	**-0.12 [-0.45, 0.21]**

Heterogeneity: Tau² = 0.48; Chi² = 104.45, df = 23 (P < 0.00001); I² = 78%
Test for overall effect: Z = 0.69 (P = 0.49)

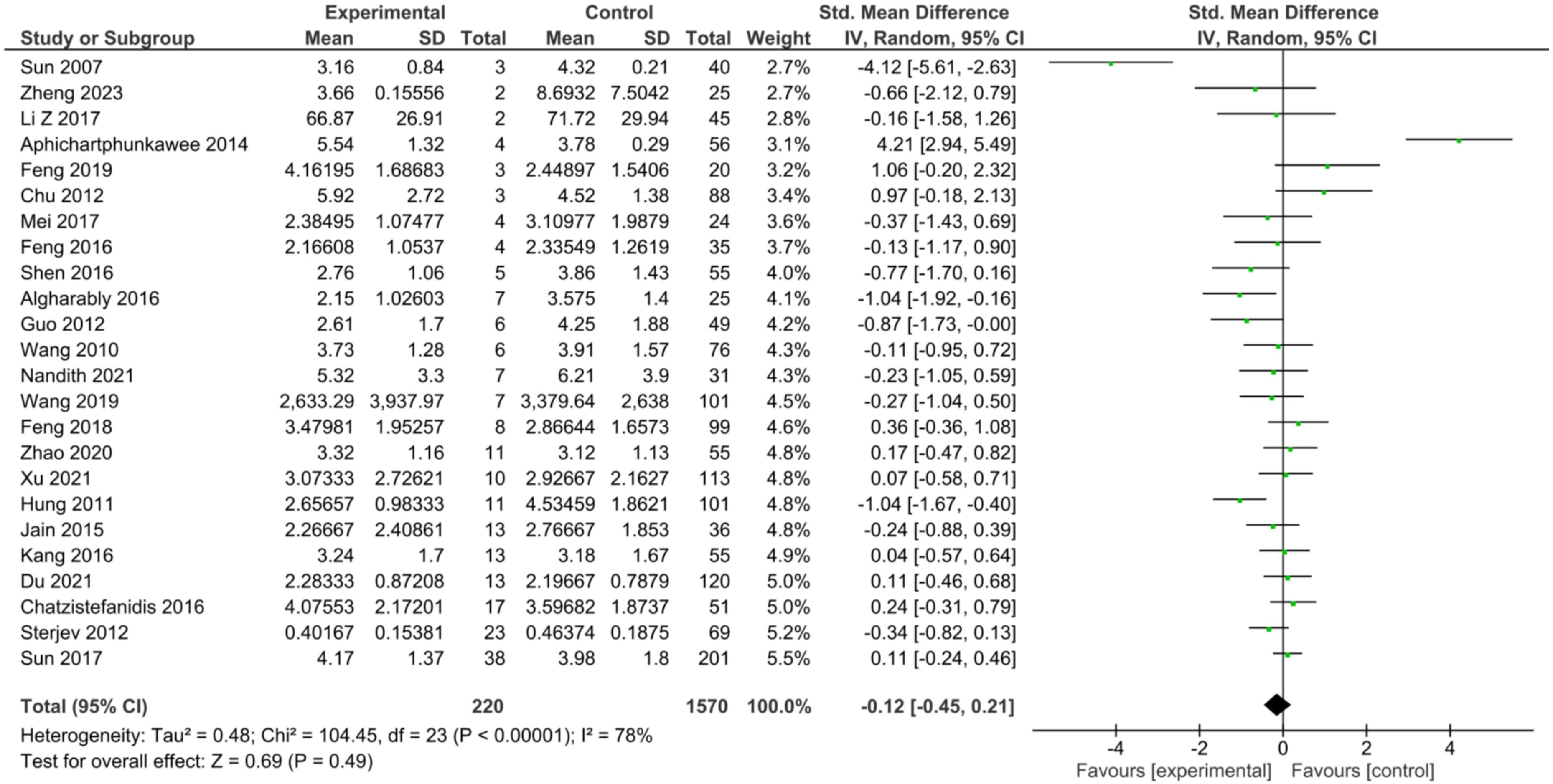

*Figure S39. Forest plot (SMD): Valproic acid C/D in UGT1A6*2 homozygous (*2 Ho) carriers and *2 non carriers (*2 None).* Homozygous carriers were defined as either 19GG, 541GG or 552CC carriers, while *2 non-carriers were defined as either 19TT, 541AA or 552AA carriers. Based on the data from k=24 trials and N=1790 patients, there was no significant effect of *2 homozygous status (SMD: -0.12 [95%CI: -0.45, 0.21]; df=23; p=0.49) on valproic acid C/D. Moderate-to-high heterogeneity was observed (I^2=78%). *C/D: Plasma concentration-to-dose ratio.*

© 2024 Milosavljević F et al. *JAMA Network Open.*

	Experimental			Control				Std. Mean Difference
Study or Subgroup	Mean	SD	Total	Mean	SD	Total	Weight	IV, Random, 95% CI
Chung 2008	2,377.7	565.92	5	1,810.2	599.36	5	2.0%	0.88 [-0.46, 2.21]
Li Z 2017	71.22	26.97	66	53.33	33.09	5	3.0%	0.65 [-0.27, 1.56]
Feng 2019	3.297645	2.199958	13	2.852852	1.825257	11	3.4%	0.21 [-0.59, 1.02]
Du 2016	3.4	1.47	50	5.5	0.33	11	3.7%	-1.54 [-2.25, -0.83]
Zheng 2023	6.7716	6.527656	25	6.528667	6.467406	15	3.9%	0.04 [-0.60, 0.68]
Sun 2015	2.11	1.26	46	3.02	1.32	14	4.0%	-0.71 [-1.32, -0.09]
Mei 2017	2.931663	2.09543	26	3.36778	1.319977	18	4.0%	-0.24 [-0.84, 0.37]
Feng 2016	2.155502	1.046795	27	2.531854	1.275749	23	4.2%	-0.32 [-0.88, 0.24]
Guo 2012	4.49	2.14	31	4.26	2.2	36	4.4%	0.10 [-0.38, 0.59]
Inoue 2014	5.1	1.32	42	4.59	1.58	29	4.4%	0.35 [-0.12, 0.83]
Wen 2016	3.5	0.2	61	3.1	0.1	79	4.5%	2.62 [2.16, 3.08]
Nandith 2021	5.54	3.9	42	5.51	2.8	38	4.6%	0.01 [-0.43, 0.45]
Zhao 2020	3.01	1.15	63	2.79	0.96	34	4.6%	0.20 [-0.22, 0.62]
Zhang 2017	5.819	3.122	47	6.296	3.664	60	4.7%	-0.14 [-0.52, 0.24]
Chu 2012	4.27	1.25	60	4.81	1.58	65	4.8%	-0.37 [-0.73, -0.02]
Chatzistefanidis 2016	3.976691	2.264694	60	3.527008	1.753596	74	4.9%	0.22 [-0.12, 0.57]
Hung 2011	3.84183	1.576108	60	3.957269	1.624757	83	4.9%	-0.07 [-0.40, 0.26]
Wang 2019	3,323.272	5,274.531	71	3,212.057	3,848.824	74	4.9%	0.02 [-0.30, 0.35]
Wang 2016	4.62	2.36	84	4.08	1.66	66	4.9%	0.26 [-0.07, 0.58]
Feng 2018	3.021977	1.75408	73	2.836112	1.588291	74	4.9%	0.11 [-0.21, 0.43]
Xu 2021	2.856667	1.998905	84	2.443333	1.889798	94	5.0%	0.21 [-0.08, 0.51]
Du 2021	2.133333	0.826741	106	2.333333	0.751486	107	5.0%	-0.25 [-0.52, 0.02]
Sun 2017	4.07	1.47	149	4.21	1.56	201	5.2%	-0.09 [-0.30, 0.12]
**Total (95% CI)**			**1291**			**1216**	**100.0%**	**0.08 [-0.16, 0.32]**

Heterogeneity: Tau² = 0.28; Chi² = 172.06, df = 22 (P < 0.00001); I² = 87%
Test for overall effect: Z = 0.67 (P = 0.51)

Std. Mean Difference
IV, Random, 95% CI

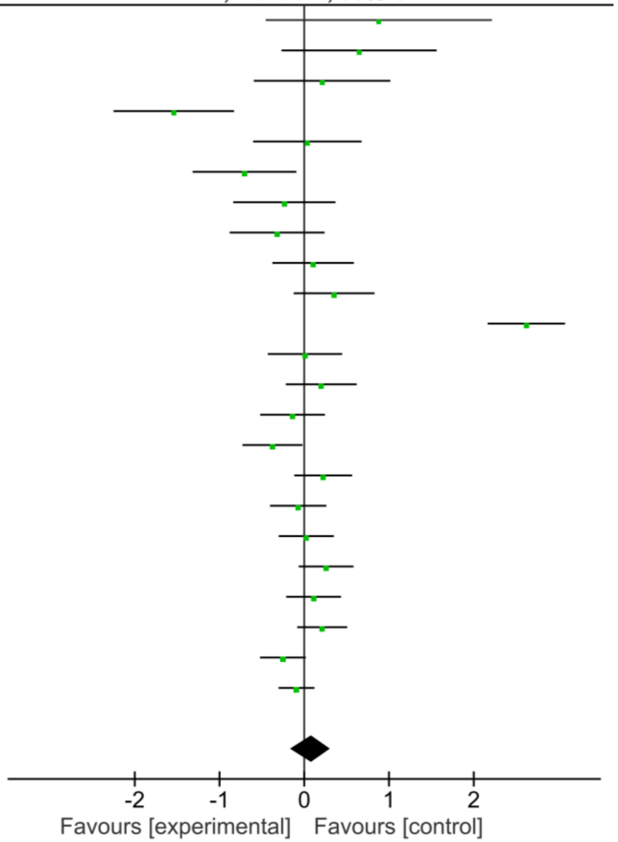

*Figure S40. Forest plot (SMD): Valproic acid C/D in UGT2B7*2 heterozygous (*2 He) carriers and *2 non carriers (*2 None).* Heterozygous carriers were defined as either 802CT or -161CT carriers, while *2 non-carriers were defined as either 802CC or -161CC carriers. Based on the data from k=23 trials and N=2507 patients, there was no significant effect of *2 heterozygous status (SMD: 0.08 [95%CI: -0.16, 0.32]; df=22; p=0.51) on valproic acid C/D. High heterogeneity was observed (I^2=87%). *C/D: Plasma concentration-to-dose ratio.*

© 2024 Milosavljević F et al. *JAMA Network Open.*

Study or Subgroup	Experimental Mean	Experimental SD	Experimental Total	Control Mean	Control SD	Control Total	Weight	Std. Mean Difference IV, Random, 95% CI
Chung 2008	2,689.98	768.87	4	1,810.2	599.36	5	1.7%	1.15 [-0.34, 2.65]
Mei 2017	3.032753	0.655592	3	3.36778	1.319977	18	2.3%	-0.25 [-1.48, 0.97]
Feng 2019	2.534876	0.960632	5	2.852852	1,825,257	11	2.8%	-0.00 [-1.06, 1.06]
Li Z 2017	58.17	34.38	13	53.33	33.09	5	2.9%	0.14 [-0.90, 1.17]
Zheng 2023	4.845	3.240782	6	6.528667	6.467406	15	3.2%	-0.28 [-1.23, 0.67]
Inoue 2014	6.19	2	7	4.59	1.58	29	3.7%	0.94 [0.08, 1.80]
Feng 2016	1.948226	0.946152	8	2.531854	1.275749	23	3.9%	-0.47 [-1.29, 0.34]
Wen 2016	2.4	0.9	9	3.1	0.888819	79	4.5%	-0.78 [-1.48, -0.08]
Zhang 2017	4.155	1.75	10	6.296	3.664	60	4.6%	-0.61 [-1.29, 0.07]
Du 2016	4.98	1.19	41	5.5	0.33	11	4.6%	-0.48 [-1.15, 0.20]
Chu 2012	4.84	1.89	11	4.81	1.58	65	4.8%	0.02 [-0.62, 0.66]
Sun 2015	2.31	1.25	42	3.02	1.32	14	5.0%	-0.55 [-1.17, 0.06]
Wang 2019	4,405.667	4,197.707	12	3,212.057	3,848.824	74	5.0%	0.30 [-0.31, 0.92]
Guo 2012	4.27	2.15	15	4.26	2.2	36	5.1%	0.00 [-0.60, 0.61]
Xu 2021	2.933333	2.009945	13	2.443333	1.889798	94	5.2%	0.26 [-0.33, 0.84]
Wang 2016	5.58	2.33	16	4.08	1.66	66	5.3%	0.82 [0.26, 1.38]
Nandith 2021	5.58	2.5	19	5.51	2.8	38	5.4%	0.03 [-0.53, 0.58]
Zhao 2020	3.51	1.03	25	2.79	0.96	34	5.5%	0.72 [0.18, 1.25]
Du 2021	2.083333	0.853463	16	2.333333	0.751486	107	5.6%	-0.32 [-0.85, 0.20]
Hung 2011	5.3387	2.277153	19	3.957269	1.624757	83	5.7%	0.78 [0.27, 1.29]
Feng 2018	3.147496	1.435993	26	2.836112	1.588291	74	6.1%	0.20 [-0.25, 0.65]
Sun 2017	3.71	1.41	45	4.21	1.56	201	6.9%	-0.32 [-0.65, -0.00]
**Total (95% CI)**			**365**			**1142**	**100.0%**	**0.04 [-0.18, 0.26]**

Heterogeneity: Tau² = 0.15; Chi² = 53.42, df = 21 (P = 0.0001); I² = 61%

Test for overall effect: Z = 0.38 (P = 0.70)

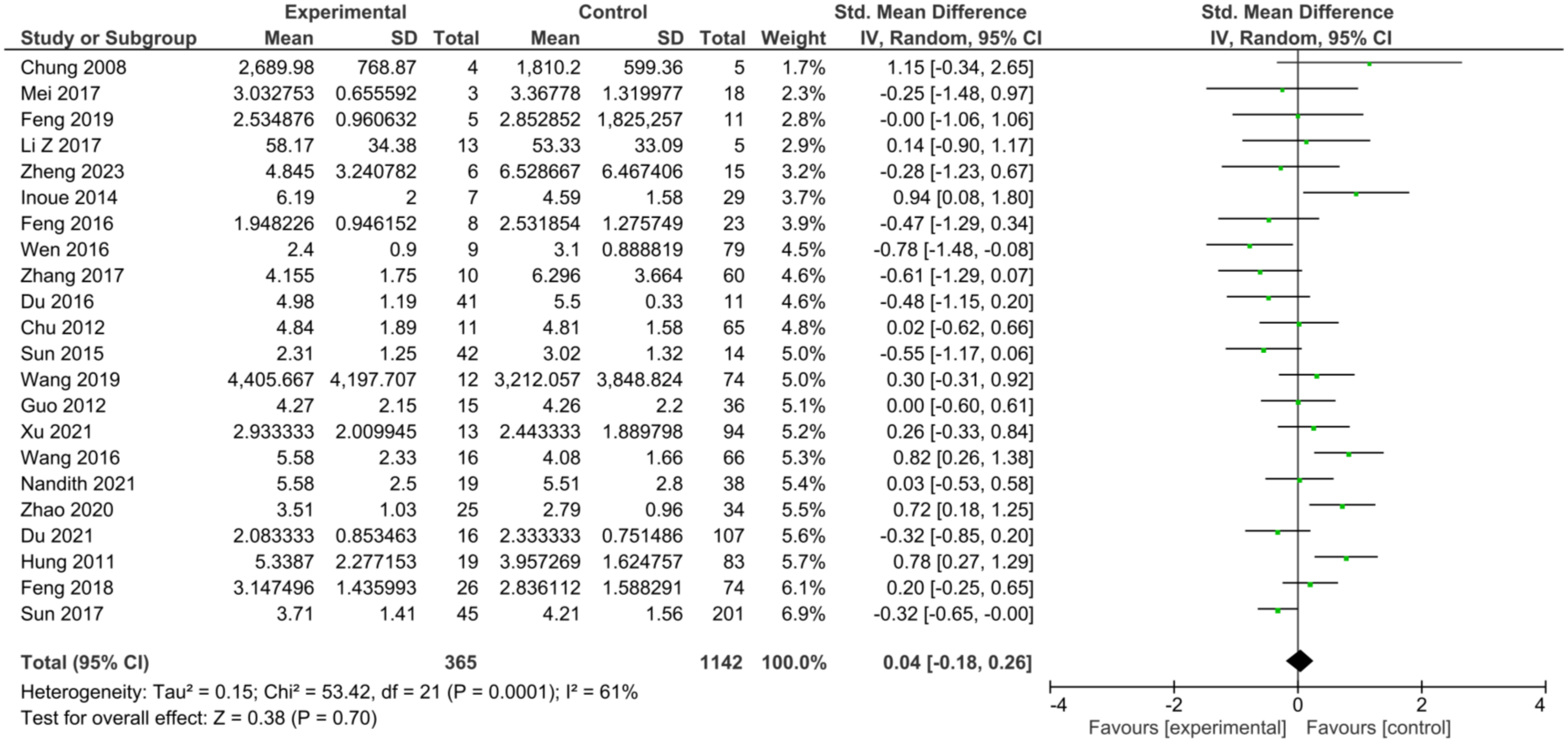

*Figure S41. Forest plot (SMD): Valproic acid C/D in UGT2B7*2 homozygous (*2 Ho) carriers and *2 non carriers (*2 None).* Homozygous carriers were defined as either 802TT or -161TT carriers, while *2 non-carriers were defined as either 802CC or -161CC carriers. Based on the data from k=22 trials and N=1507 patients, there was no significant effect of *2 homozygous status (SMD: 0.04 [95%CI: -0.18, 0.26]; df=21; p=0.70) on valproic acid C/D. Moderate heterogeneity was observed (I^2=61%). *C/D: Plasma concentration-to-dose ratio.*

© 2024 Milosavljević F et al. *JAMA Network Open.*

Study or Subgroup	Experimental Mean	Experimental SD	Experimental Total	Control Mean	Control SD	Control Total	Weight	Std. Mean Difference IV, Random, 95% CI
Feng 2019	2.39412	2.13144	6	3.33219	1.84879	20	1.2%	-0.48 [-1.40, 0.45]
Mei 2017	2.5458	1.67086	9	3.24196	1.86158	37	2.0%	-0.37 [-1.11, 0.36]
Zheng 2023	6.647	6.16893	10	6.50914	6.22155	35	2.2%	0.02 [-0.68, 0.72]
Nandith 2021	6.79	3.4	17	5.28	3.2	80	3.8%	0.46 [-0.06, 0.99]
Shen 2016	3.5	1.73	23	3.73	1.49	70	4.8%	-0.15 [-0.62, 0.32]
Sun 2015	2.303	1.38	23	2.28	1.32	78	4.9%	0.02 [-0.45, 0.48]
Du 2016	4.05	1.88	26	4.39	1.38	74	5.3%	-0.22 [-0.67, 0.23]
Feng 2018	3.10223	1.58979	43	2.92034	1.65057	126	8.9%	0.11 [-0.24, 0.46]
Wang 2016	4.12	2.02	45	4.64	2.19	119	9.0%	-0.24 [-0.59, 0.10]
Du 2021	2.13333	0.61309	44	2.23333	0.67257	181	9.8%	-0.15 [-0.48, 0.18]
Xu 2021	2.37333	1.61154	49	2.72	1.75322	137	10.0%	-0.20 [-0.53, 0.13]
Ma 2013	3.612	2.83656	90	3.799	3.04321	147	15.5%	-0.06 [-0.33, 0.20]
Sun 2017	4.19	1.22	121	4.23	1.43	256	22.7%	-0.03 [-0.25, 0.19]
**Total (95% CI)**			**506**			**1360**	**100.0%**	**-0.08 [-0.18, 0.03]**

Heterogeneity: Tau² = 0.00; Chi² = 9.04, df = 12 (P = 0.70); I² = 0%
Test for overall effect: Z = 1.45 (P = 0.15)

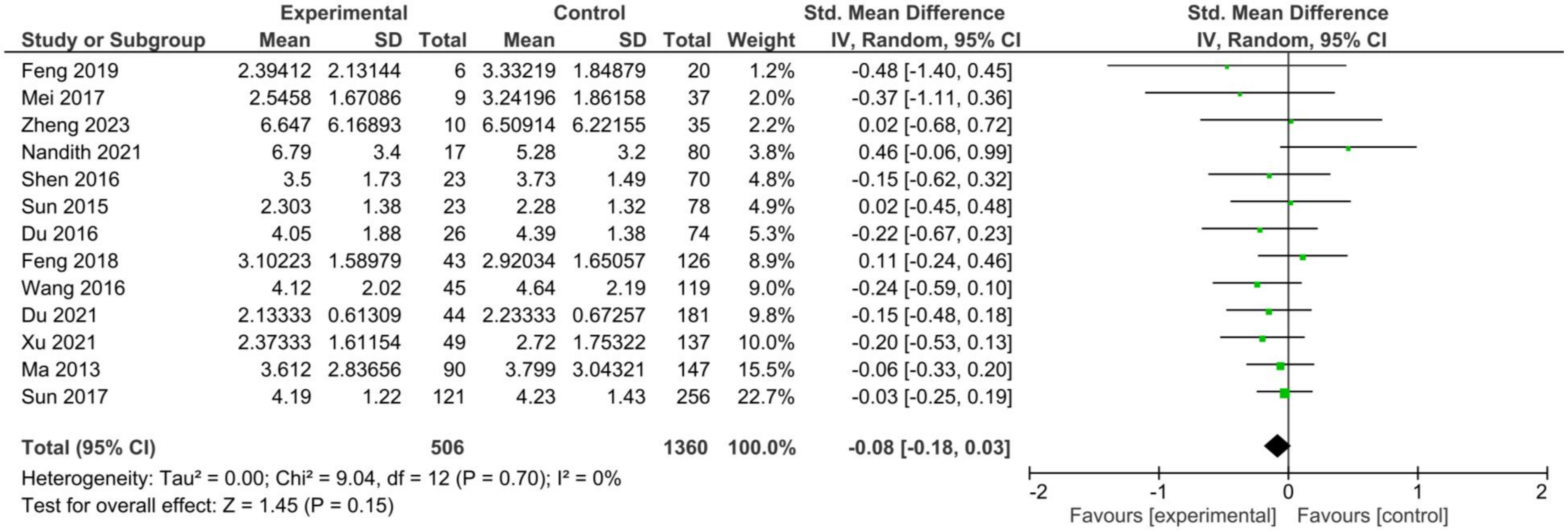

*Figure S42. Forest plot (SMD): Valproic acid C/D in UGT2B7*3 heterozygous (*3 He) carriers and *3 non carriers (*3 None).* Heterozygous carriers were defined as 211GT carriers, while *3 non-carriers were defined as 211GG carriers. Based on the data from k=13 trials and N=1866 patients, there was no significant effect of *3 heterozygous status (SMD: -0.08 [95%CI: -0.18, 0.03]; df=12; p=0.15) on valproic acid C/D. No heterogeneity was observed (I^2=0%). *C/D: Plasma concentration-to-dose ratio.*

© 2024 Milosavljević F et al. *JAMA Network Open.*

Study or Subgroup	Experimental Mean	Experimental SD	Experimental Total	Control Mean	Control SD	Control Total	Weight	Std. Mean Difference IV, Random, 95% CI
Feng 2019	1.972187	1.077036	3	3.332194	1.848787	20	10.0%	-0.73 [-1.97, 0.50]
Feng 2018	3.082904	2.132603	3	2.920338	1.650573	126	11.0%	0.10 [-1.05, 1.24]
Shen 2016	2.63	0.45	4	3.73	1.49	70	12.5%	-0.74 [-1.76, 0.27]
Du 2021	2.946667	2.347921	4	2.233333	0.672571	181	12.8%	0.97 [-0.02, 1.97]
Xu 2021	4.15	3.308524	5	2.72	1.753224	137	14.1%	0.78 [-0.11, 1.68]
Ma 2013	1.759	1.03147	11	3.799	3.043213	147	18.7%	-0.69 [-1.30, -0.07]
Sun 2017	4.13	1.62	17	4.23	1.43	256	20.9%	-0.07 [-0.56, 0.42]
**Total (95% CI)**			**47**			**937**	**100.0%**	**-0.06 [-0.56, 0.43]**

Heterogeneity: Tau² = 0.24; Chi² = 14.32, df = 6 (P = 0.03); I² = 58%
Test for overall effect: Z = 0.25 (P = 0.80)

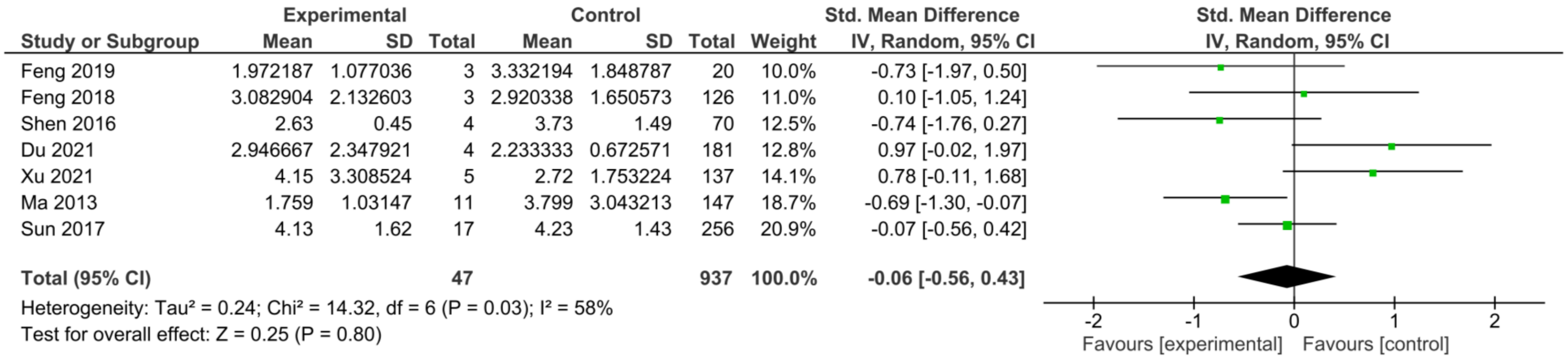

*Figure S43. Forest plot (SMD): Valproic acid C/D in UGT2B7*3 homozygous (*3 Ho) carriers and *3 non carriers (*3 None).* Homozygous carriers were defined as 211TT carriers, while *3 non-carriers were defined as 211GG carriers. Based on the data from k=7 trials and N=984 patients, there was no significant effect of *3 homozygous status (SMD: -0.06 [95%CI: -0.56], 0.43; df=6; p=0.80) on valproic acid C/D. Low-to-moderate heterogeneity was observed (I^2=58%). *C/D: Plasma concentration-to-dose ratio.*

© 2024 Milosavljević F et al. *JAMA Network Open.*

7.3) Lamotrigine related SMD meta-analyses (Figures S44-S46)

	Experimental			Control				Std. Mean Difference
**Study or Subgroup**	**Mean**	**SD**	**Total**	**Mean**	**SD**	**Total**	**Weight**	**IV, Random, 95% CI**
Suzuki 2019b	10.4	4.1	5	18.2	13.1	13	4.5%	-0.64 [-1.70, 0.41]
Petrenaite 2018	0.056	0.037	8	0.057	0.017	25	5.6%	-0.04 [-0.84, 0.75]
Suzuki 2019a	10.7	4.9	14	12	7.5	22	6.3%	-0.19 [-0.86, 0.48]
Liu 2015a	1.2	0.81	22	0.87	0.35	34	6.9%	0.57 [0.02, 1.11]
Ortega-Vázquez 2020	2.1457	1.573093	20	2.371	1.78	58	7.1%	-0.13 [-0.64, 0.38]
Reimers 2016	0.06	0.038069	19	0.07	0.036364	127	7.3%	-0.27 [-0.76, 0.21]
He 2017	1.276564	0.312123	33	1.102789	0.319721	39	7.3%	0.54 [0.07, 1.02]
Chang 2014	4.51	0.66	24	2.33	2.93	82	7.3%	0.83 [0.36, 1.30]
Du 2016	2.163571	0.720811	28	2.77	0.87	74	7.4%	-0.72 [-1.17, -0.28]
Petrenaite 2022	4.11	1.65	38	3.96	1.71	157	7.9%	0.09 [-0.27, 0.44]
Wang 2015	2.9	1.84	46	2.31	1.2	102	7.9%	0.41 [0.06, 0.76]
Liu 2015b	2.75	1.02	49	2.14	0.96	109	7.9%	0.62 [0.28, 0.96]
Smith 2018	0.067845	0.035475	87	0.124325	0.064678	447	8.3%	-0.93 [-1.16, -0.69]
Božina 2023	101.7381	77.32093	106	107.2786	82.60454	365	8.4%	-0.07 [-0.28, 0.15]
**Total (95% CI)**			**499**			**1654**	**100.0%**	**0.02 [-0.30, 0.34]**

Heterogeneity: Tau² = 0.31; Chi² = 109.85, df = 13 (P < 0.00001); I² = 88%
Test for overall effect: Z = 0.12 (P = 0.90)

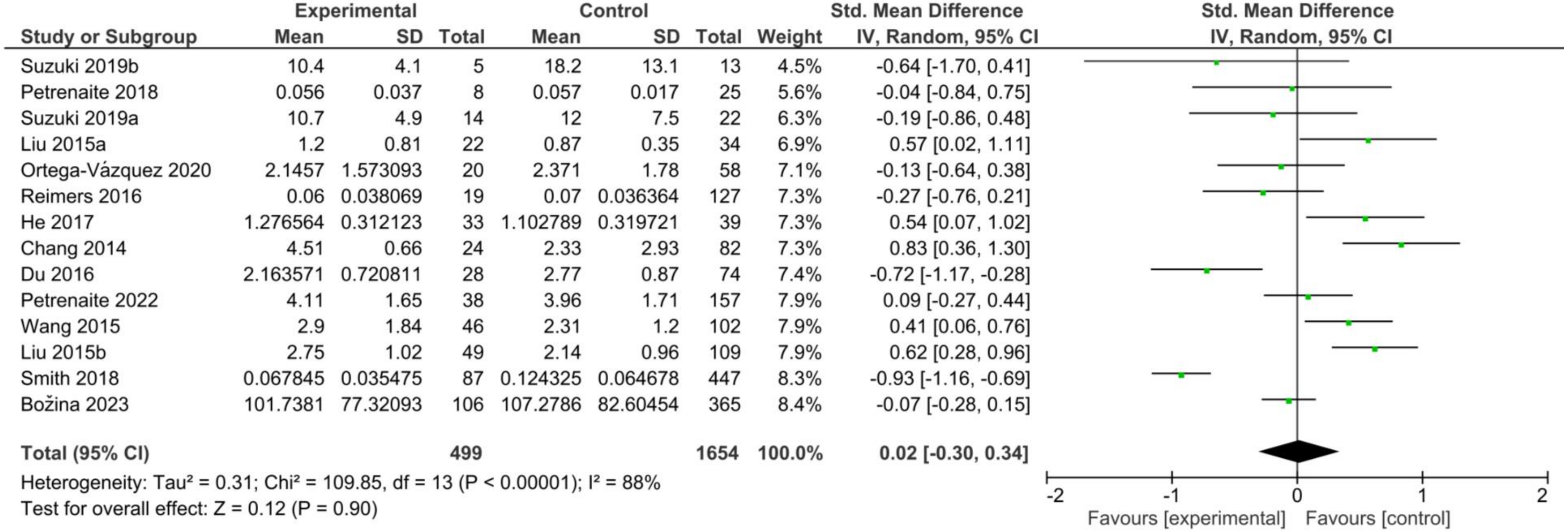

*Figure S44. Forest plot (SMD): Lamotrigine C/D in UGT1A4*3 non-carriers compared to UGT1A4*3 carriers.* Based on the data from 14 cohorts from k=12 trials and N=2153 patients, *3 heterozygous stats had no significant effect (SMD: 0.02 [95%CI: -0.30, 0.34]; df=13; p=0.90) on lamotrigine C/D. High level of heterogeneity was observed (I^2=88%). *C/D: Plasma concentration-to-dose ratio.*

© 2024 Milosavljević F et al. *JAMA Network Open.*

Study or Subgroup	Experimental Mean	Experimental SD	Experimental Total	Control Mean	Control SD	Control Total	Weight	Std. Mean Difference IV, Random, 95% CI
Suzuki 2019b	18.2	13.1	13	10.4	4.1	5	1.6%	0.64 [-0.41, 1.70]
Petrenaite 2018	0.054	0.018	18	0.051	0.017	7	2.3%	0.16 [-0.71, 1.04]
Suzuki 2019a	12	7.5	22	10.7	4.9	14	3.9%	0.19 [-0.48, 0.86]
Ortega-Vázquez 2020	2.08	1.62	35	2.579	1.877	60	10.1%	-0.28 [-0.70, 0.14]
Petrenaite 2022	3.71	1.47	105	3.8	1.93	42	13.8%	-0.06 [-0.41, 0.30]
Zhou 2015	1.296667	0.418699	55	1.196667	0.370195	77	14.7%	0.25 [-0.09, 0.60]
Wang 2016	2.46	1.33	84	2.42	1.04	66	17.0%	0.03 [-0.29, 0.36]
Božina 2023	104.4982	77.32864	237	104.0007	75.9205	119	36.5%	0.01 [-0.21, 0.23]
**Total (95% CI)**			**569**			**390**	**100.0%**	**0.03 [-0.10, 0.16]**

Heterogeneity: Tau² = 0.00; Chi² = 5.53, df = 7 (P = 0.60); I² = 0%

Test for overall effect: Z = 0.46 (P = 0.65)

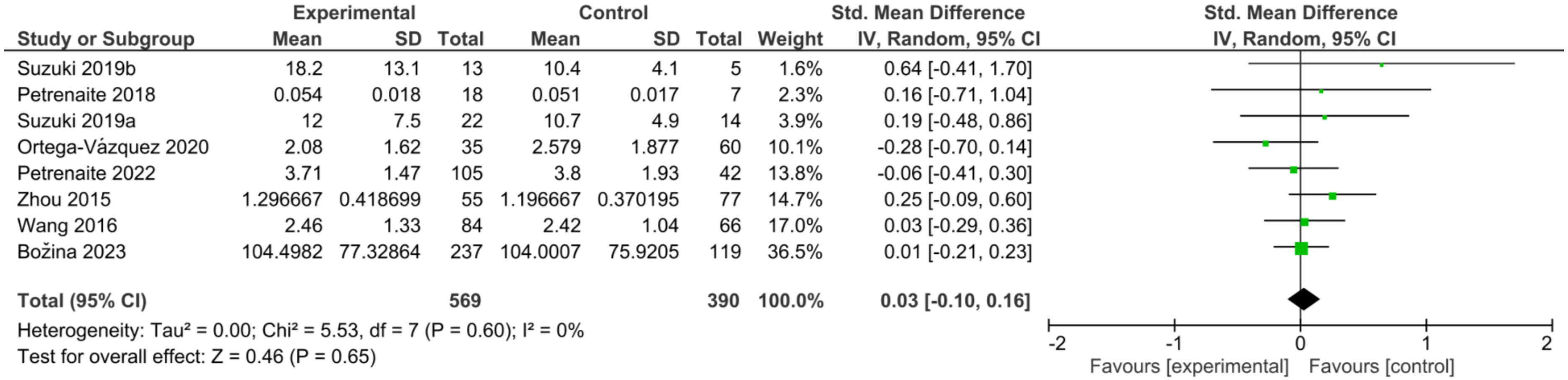

*Figure S45. Forest plot (SMD): Lamotrigine C/D in UGT2B7*2 heterozygous (*2 He) carriers and *2 non carriers (*2 None).* Heterozygous carriers were defined as either 802CT or -161CT carriers, while *2 non-carriers were defined as either 802CC or -161CC carriers. Based on the data from 8 cohorts from k=7 trials and N=959 patients, there was no significant effect of *2 heterozygous status (SMD: 0.03 [95%CI: -0.10, 0.16]; df=7; p=0.65) on lamotrigine C/D. No heterogeneity was observed (I^2=0%). *C/D: Plasma concentration-to-dose ratio.*

© 2024 Milosavljević F et al. *JAMA Network Open.*

Study or Subgroup	Experimental Mean	Experimental SD	Experimental Total	Control Mean	Control SD	Control Total	Weight	Std. Mean Difference IV, Random, 95% CI
Petrenaite 2018	0.07	0.031	8	0.051	0.017	7	10.7%	0.70 [-0.35, 1.76]
Zhou 2015	1.576667	0.804157	8	1.196667	0.370195	77	16.1%	0.89 [0.15, 1.63]
Wang 2016	1.92	0.87	16	2.42	1.04	66	20.6%	-0.49 [-1.04, 0.06]
Petrenaite 2022	4.68	1.81	52	3.8	1.93	42	24.3%	0.47 [0.06, 0.88]
Božina 2023	104.5009	82.55575	115	104.0007	75.9205	119	28.4%	0.01 [-0.25, 0.26]
**Total (95% CI)**			**199**			**311**	**100.0%**	**0.23 [-0.19, 0.66]**

Heterogeneity: Tau² = 0.15; Chi² = 13.48, df = 4 (P = 0.009); I² = 70%
Test for overall effect: Z = 1.07 (P = 0.28)

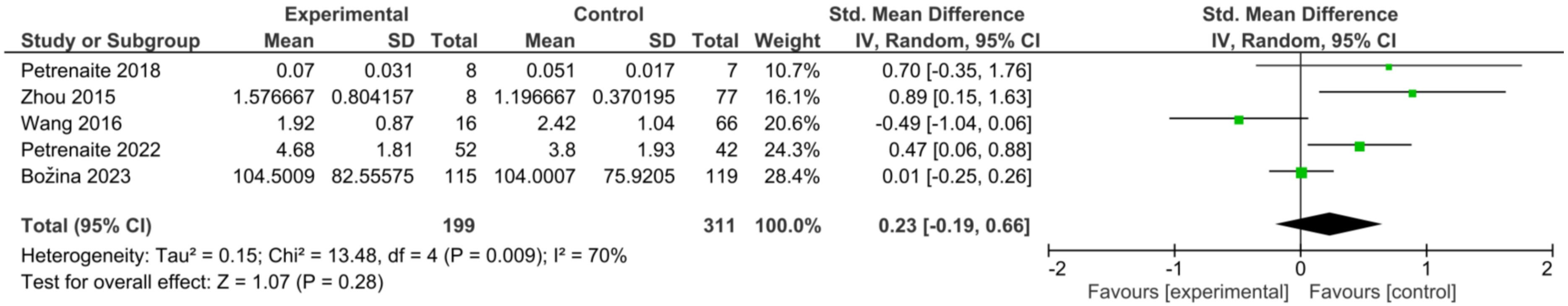

*Figure S46. Forest plot (SMD): Lamotrigine C/D in UGT2B7*2 homozygous (*2 Ho) carriers and *2 non carriers (*2 None).* Homozygous carriers were defined as either 802TT or -161TT carriers, while *2 non-carriers were defined as either 802CC or -161CC carriers. Based on the data from k=5 trials and N=510 patients, there was no significant effect of *2 homozygous status (SMD: 0.23 [95%CI: -0.19, 0.66]; df=4; p=0.28) on lamotrigine C/D. Substantial heterogeneity was observed (I^2=70%). *C/D: Plasma concentration-to-dose ratio.*

© 2024 Milosavljević F et al. *JAMA Network Open.*

7.4) Carbamazepine related SMD meta-analyses (Figures S47-S52)

Study or Subgroup	Experimental Mean	Experimental SD	Experimental Total	Control Mean	Control SD	Control Total	Weight	Std. Mean Difference IV, Random, 95% CI
Pham 2020	9.1	3.7	6	7.9	3.5	32	3.7%	0.33 [-0.54, 1.21]
Park 2009	13.1	4.5	21	9.9	3.4	14	5.1%	0.76 [0.06, 1.46]
Liu 2014	0.3	0.11	13	0.21	0.08	27	5.1%	0.97 [0.28, 1.67]
Panomvana 2013	10.61	3.65	15	11.06	3.92	21	5.4%	-0.12 [-0.78, 0.55]
Lu 2018	10.64339	6.335114	33	10.49875	6.057666	29	7.5%	0.02 [-0.48, 0.52]
Wang 2015	21.23581	10.27222	32	18.4378	5.353287	34	7.6%	0.34 [-0.15, 0.83]
Liu 2016	0.27	0.21	32	0.344	0.262238	35	7.7%	-0.31 [-0.79, 0.18]
Meng 2011	1.19	0.38	52	0.9	0.2	32	8.0%	0.89 [0.43, 1.35]
Ganesapandian 2019	0.59	0.435225	31	0.37	0.393727	69	8.5%	0.54 [0.11, 0.97]
Saruwatari 2014	0.929415	0.149663	58	0.886588	0.252446	46	9.2%	0.21 [-0.18, 0.60]
Seo 2006	0.642445	0.253579	75	0.66451	0.264951	69	10.3%	-0.08 [-0.41, 0.24]
Ma 2015	0.92084	0.245593	80	0.903167	0.25234	86	10.7%	0.07 [-0.23, 0.38]
Zhu 2014	0.83	0.403361	132	0.695513	0.316711	78	11.1%	0.36 [0.08, 0.64]
**Total (95% CI)**			**580**			**572**	**100.0%**	**0.27 [0.08, 0.47]**

Heterogeneity: Tau² = 0.07; Chi² = 28.38, df = 12 (P = 0.005); I² = 58%
Test for overall effect: Z = 2.74 (P = 0.006)

Std. Mean Difference IV, Random, 95% CI

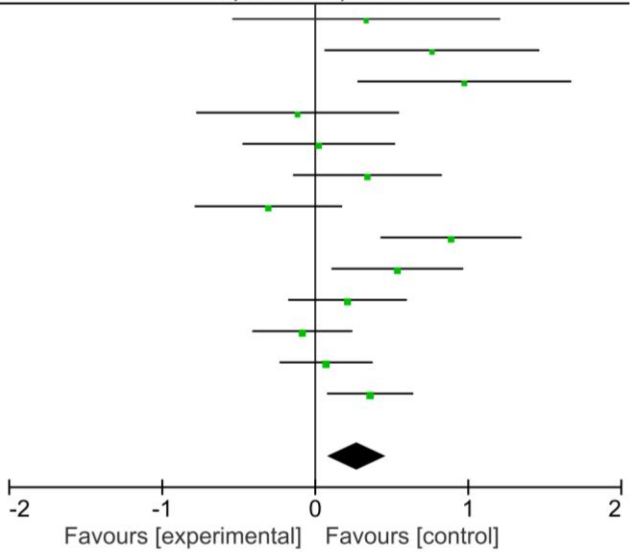

Figures S47. Forest plot (SMD): Carbamazepine active moiety C/D in CYP3A5 non-expressors and expressors. CYP3A5 non-expressors were defined as the carriers of *3/*3 genotype, while every other participant was classified as CYP3A5 expressor. Based on the data from k=13 trials and N=1152 patients, CYP3A5 non-expressor status had small effect (SMD: 0.27 [95%: 0.08, 0.47]; df=12; p=0.006) on carbamazepine active moiety C/D. Moderate heterogeneity was observed (I^2=58%). *C/D: Plasma concentration-to-dose ratio.*

© 2024 Milosavljević F et al. *JAMA Network Open.*

	Experimental			Control				Std. Mean Difference
Study or Subgroup	Mean	SD	Total	Mean	SD	Total	Weight	IV, Random, 95% CI
Yun 2013	1.1	0.38	37	1.15	0.56	19	12.8%	-0.11 [-0.66, 0.44]
Chbili 2016	0.97	0.326037	16	0.96	0.558301	78	12.9%	0.02 [-0.52, 0.56]
Venkatraman 2023	0.538467	0.406052	63	0.432679	0.230165	35	14.2%	0.30 [-0.12, 0.71]
Ma 2015	0.812448	0.248441	81	1.013042	0.265829	51	14.8%	-0.78 [-1.14, -0.42]
Hung 2012	9.575426	2.734538	113	14.76781	6.655323	58	15.0%	-1.16 [-1.50, -0.82]
Daci 2015	1.12	0.677717	72	1.06	0.559732	63	15.0%	0.10 [-0.24, 0.43]
Zhu 2014	0.75	0.371618	115	0.83	0.353836	68	15.3%	-0.22 [-0.52, 0.08]
**Total (95% CI)**			**497**			**372**	**100.0%**	**-0.28 [-0.68, 0.13]**

Heterogeneity: Tau² = 0.26; Chi² = 46.32, df = 6 (P < 0.00001); I² = 87%
Test for overall effect: Z = 1.33 (P = 0.18)

Std. Mean Difference
IV, Random, 95% CI

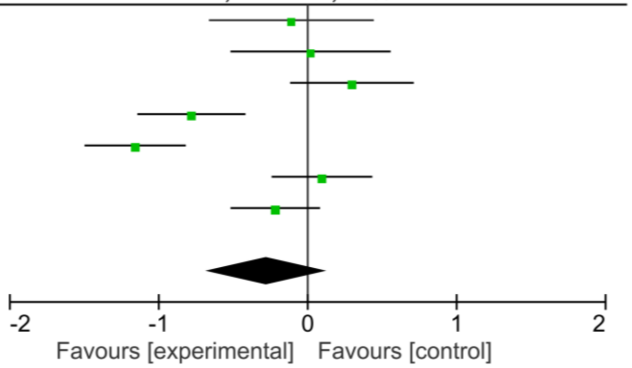

Figure S48. Forest plot (SMD): Carbamazepine active moiety C/D in EPHX1 337CT and 337TT carriers. Carriers of 337CT genotype are heterozygous carriers of mutated allele while 337TT carriers are considered the controls. Based on the data from k=7 trials and N=869 patients, there was no significant effect of 337CT status (SMD: -0.28 [95%CI: -0.68, 0.13]; df=6; p=0.18) on carbamazepine active moiety C/D. High heterogeneity was observed (I^2=87%). *C/D: Plasma concentration-to-dose ratio.*

© 2024 Milosavljević F et al. *JAMA Network Open.*

Study or Subgroup	Experimental Mean	Experimental SD	Experimental Total	Control Mean	Control SD	Control Total	Weight	Std. Mean Difference IV, Random, 95% CI
Daci 2015	0.83	0.180278	10	1.06	0.5597321	63	13.6%	-0.43 [-1.10, 0.24]
Yun 2013	0.97	0.3	27	1.15	0.56	19	14.0%	-0.42 [-1.01, 0.18]
Venkatraman 2023	0.510797	0.624855	17	0.432679	0.2301651	35	14.0%	0.19 [-0.39, 0.77]
Chbili 2016	1.72	0.326037	24	0.96	0.558301	78	14.4%	1.47 [0.97, 1.97]
Zhu 2014	0.81	0.441928	27	0.83	0.3538361	68	14.6%	-0.05 [-0.50, 0.39]
Ma 2015	0.916409	0.231947	34	1.013042	0.2658291	51	14.7%	-0.38 [-0.82, 0.06]
Hung 2012	7.737278	3.136001	63	14.76781	6.6553229	58	14.8%	-1.36 [-1.76, -0.96]
**Total (95% CI)**			**202**			**372**	**100.0%**	**-0.14 [-0.83, 0.54]**

Heterogeneity: Tau² = 0.79; Chi² = 78.97, df = 6 (P < 0.00001); I² = 92%
Test for overall effect: Z = 0.41 (P = 0.68)

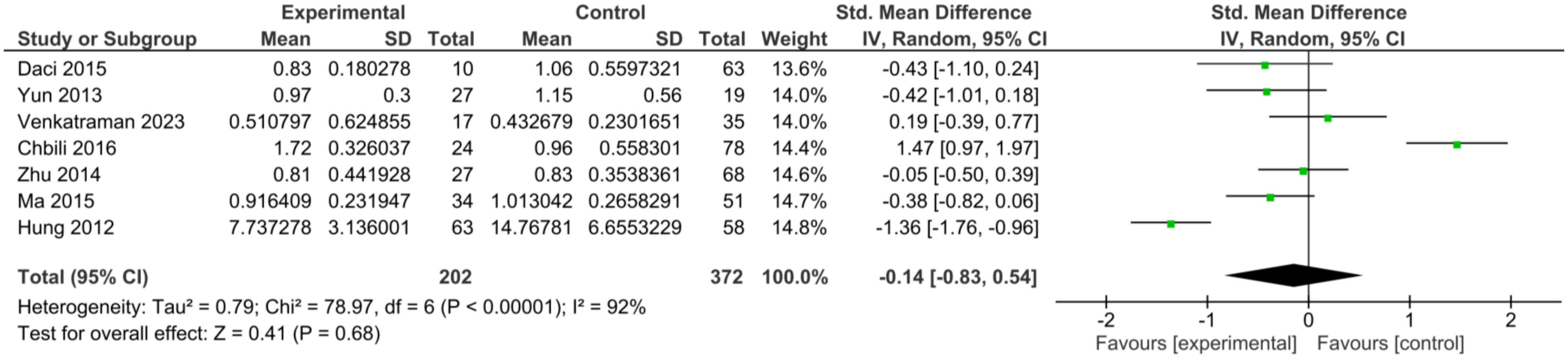

Figure S49. Forest plot (SMD): Carbamazepine active moiety C/D in EPHX1 337CC and 337TT carriers. Carriers of 337CC genotype are homozygous carriers of mutated allele while 337TT carriers are considered the controls. Based on the data from k=7 trials and N=574 patients, there was no significant effect of 337CC status (SMD: -0.14 [95%CI: -0.83, 0.54]; df=6; p=0.68) on carbamazepine active moiety C/D. High heterogeneity was observed (I^2=92%). *C/D: Plasma concentration-to-dose ratio.*

© 2024 Milosavljević F et al. *JAMA Network Open.*

Study or Subgroup	Experimental Mean	Experimental SD	Experimental Total	Control Mean	Control SD	Control Total	Weight	Std. Mean Difference IV, Random, 95% CI
Chbili 2016	0.95	0.193132	13	0.97	0.5026927	98	14.8%	-0.04 [-0.62, 0.54]
Yun 2013	1.34	0.59	17	1	0.32	64	15.6%	0.86 [0.31, 1.42]
Zhu 2014	0.73	0.3228	36	0.78	0.3535534	172	22.2%	-0.14 [-0.50, 0.22]
Daci 2015	1.03	0.556687	55	1.12	0.6878953	80	22.9%	-0.14 [-0.48, 0.20]
Hung 2012	10.71077	5.074466	55	10.03471	4.0671737	176	24.5%	0.16 [-0.15, 0.46]
**Total (95% CI)**			**176**			**590**	**100.0%**	**0.10 [-0.20, 0.41]**

Heterogeneity: Tau² = 0.07; Chi² = 11.17, df = 4 (P = 0.02); I² = 64%
Test for overall effect: Z = 0.67 (P = 0.51)

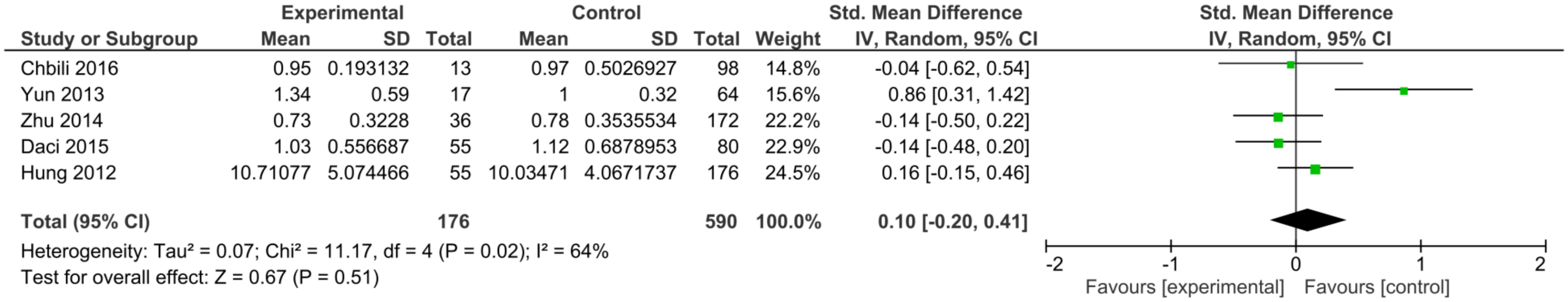

Figure S50. Forest plot (SMD): Carbamazepine active moiety C/D in EPHX1 416AA and 416AG carriers. Carriers of 416AG genotype are heterozygous carriers of mutated allele while 416AA carriers are considered the controls. Based on the data from k=5 trials and N=766 patients, there was no significant effect of 416AG status (SMD: 0.10 [95%CI: -0.20, 0.41]; df=4; p=0.51) on Carbamazepine active moiety C/D. Moderate heterogeneity was observed (I^2=64%). No reliable meta-analysis on 416GG carriers was not possible due to low amount available data. *C/D: Plasma concentration-to-dose ratio.*

Study or Subgroup	Experimental Mean	Experimental SD	Experimental Total	Control Mean	Control SD	Control Total	Weight	Std. Mean Difference IV, Random, 95% CI
Lu 2018	10.40625	4.615728	32	14.00794	7.441845	28	13.1%	-0.58 [-1.10, -0.06]
Zhang 2018	1.465	0.67	30	1.602	0.74	40	14.9%	-0.19 [-0.67, 0.28]
Venkatraman 2023	0.451228	0.364224	47	0.531476	0.432303	49	18.8%	-0.20 [-0.60, 0.20]
Ma 2015	0.92085	0.245179	79	0.919863	0.249797	68	24.3%	0.00 [-0.32, 0.33]
Hung 2012	10.49868	4.973985	84	9.974182	4.154709	133	28.9%	0.12 [-0.16, 0.39]
**Total (95% CI)**			**272**			**318**	**100.0%**	**-0.11 [-0.32, 0.11]**

Heterogeneity: Tau² = 0.02; Chi² = 6.39, df = 4 (P = 0.17); I² = 37%
Test for overall effect: Z = 0.98 (P = 0.33)

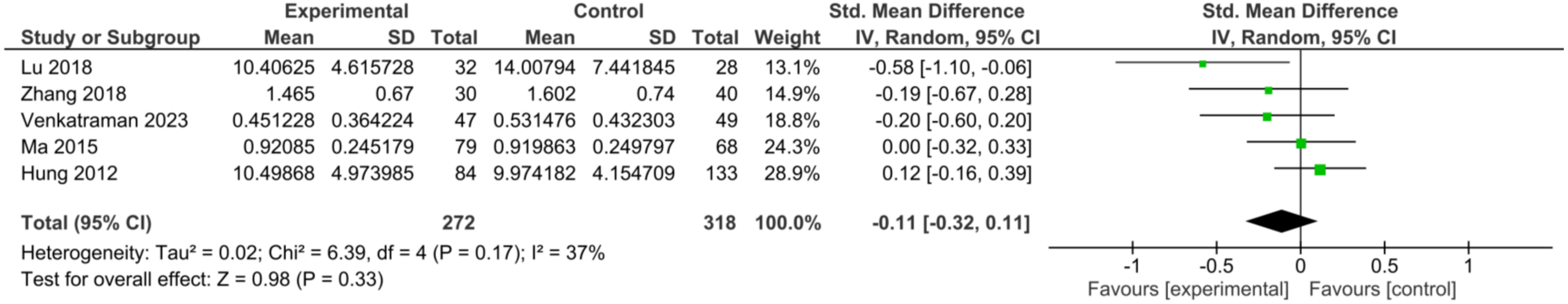

*Figure S51. Forest plot (SMD): Carbamazepine active-moiety C/D in UGT2B7*2 heterozygous (*2 He) carriers and *2 non carriers (*2 None).* Heterozygous carriers were defined as either 802CT or -161CT carriers, while *2 non-carriers were defined as either 802CC or -161CC carriers. Based on the data from k=5 trials and N=590 patients, there was no significant effect of *2 heterozygous status (SMD: -0.11 [CI95%: -0.32, 0.11]; df=4; p=0.33) on carbamazepine active-moiety C/D. Low heterogeneity was observed (I^2=37%). *C/D: Plasma concentration-to-dose ratio.*

© 2024 Milosavljević F et al. *JAMA Network Open.*

Study or Subgroup	Experimental Mean	Experimental SD	Experimental Total	Control Mean	Control SD	Control Total	Weight	Std. Mean Difference IV, Random, 95% CI
Zhang 2018	1.261	0.647	8	1.602	0.74	40	16.0%	-0.46 [-1.23, 0.30]
Venkatraman 2023	0.576219	0.343156	19	0.531476	0.432303	49	27.0%	0.11 [-0.42, 0.64]
Ma 2015	0.847636	0.258108	19	0.919863	0.249797	68	28.4%	-0.28 [-0.79, 0.23]
Hung 2012	11.42781	4.631813	17	9.974182	4.154709	133	28.6%	0.34 [-0.16, 0.85]
**Total (95% CI)**			**63**			**290**	**100.0%**	**-0.03 [-0.37, 0.32]**

Heterogeneity: Tau² = 0.04; Chi² = 4.51, df = 3 (P = 0.21); I² = 34%
Test for overall effect: Z = 0.15 (P = 0.88)

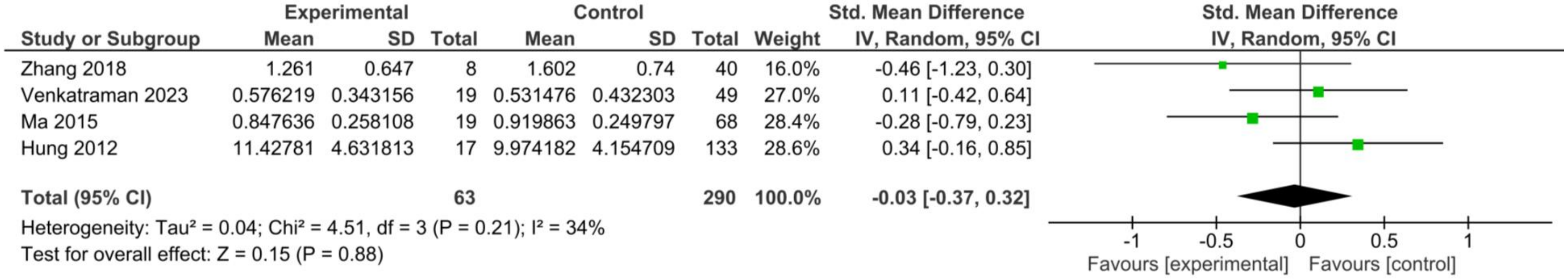

*Figure S52. Forest plot (SMD): Carbamazepine active-moiety C/D in UGT2B7*2 homozygous (*2 Ho) carriers and *2 non carriers (*2 None).* Homozygous carriers were defined as either 802TT or -161TT carriers, while *2 non-carriers were defined as either 802CC or -161CC carriers. Based on the data from k=4 trials and N=353 patients, there was no significant effect of *2 homozygous status (SMD: -0.03 [CI95%: -0.37, 0.32]; df=3; p=0.88) on carbamazepine active-moiety C/D. Low heterogeneity was observed (I^2=34%). *C/D: Plasma concentration-to-dose ratio.*

© 2024 Milosavljević F et al. *JAMA Network Open.*
