# Supplementary material for: Pharmacogenetic Variants and Plasma Concentrations of Antiseizure Drugs: A Systematic Review and Meta-Analysis
Source: JAMA Netw Open. 2024 Aug 8;7(8):e2425593. doi: 10.1001/jamanetworkopen.2024.25593 (PMC11310823; doi:10.1001/jamanetworkopen.2024.25593)
Supplement: Supplement 2. — Data Sharing Statement [file jamanetwopen-e2425593-s002.pdf]

Data Sharing Statement

Milosavljević. Pharmacogenetic Variants and Plasma Concentrations of Antiseizure Drugs. *JAMA Netw Open*. Published August 08, 2024. doi:10.1001/jamanetworkopen.2024.25593

Data
Data available: Yes
Data types: Data (not involving human participants)
How to access data: marin.jukic@ki.se
When available: With publication

Supporting Documents
Document types: None

Additional Information
Who can access the data: Anyone requesting the data
Types of analyses: For any purpose
Mechanisms of data availability: With investigator support
